# Functional Compounds of Cold-Pressed Pomegranate Seed Oil: Fatty Acids and Phytosterols Profile as Quality Biomarkers for Origin Discrimination

**DOI:** 10.3390/foods12132599

**Published:** 2023-07-05

**Authors:** Giuseppe Iriti, Sonia Bonacci, Vincenzo Lopreiato, Marialaura Frisina, Manuela Oliverio, Antonio Procopio

**Affiliations:** 1 Department of Health Science, University Magna Græcia of Catanzaro, 88100 Catanzaro, Italy; giuseppe.iriti@studenti.unicz.it (G.I.); m.frisina@unicz.it (M.F.); m.oliverio@unicz.it (M.O.); procopio@unicz.it (A.P.); 2 Department of Veterinary Sciences, University of Messina, 98122 Messina, Italy; vincenzo.lopreiato@unime.it

**Keywords:** pomegranate seed oil, punicic acid, phytosterols, tocopherols, phenols, principal component analysis

## Abstract

Cold-pressed pomegranate seed oil (PSO) is a product of the extraction of non-edible pomegranate seeds. Its unique chemical composition in terms of both polyunsaturated fatty acids, especially punicic acid (PA), and secondary metabolites, such as phytosterols, tocopherols and phenols, make it an interesting functional ingredient for food enrichment. It is not clear if the biomarkers profile of PSO depends to factors connected to the geographical origin of seeds. This work presents a statistical comparative analysis, concerning biomolecules composition and geographical origin of 32 commercial cold-pressed PSOs, performed by principal component analysis. The study discriminates between Turkish and Italian PSOs, on the base of the fatty acid profile and phytosterols, and not on the tocopherols and phenols. These results confirmed PA as the main characteristic biomarker of oil genuineness and, for the first time, disclosed a statistically relevant variability of phytosterols, which can be proposed as quality biomarkers for discrimination of geographical origins.

## 1. Introduction

**Figure 1:**
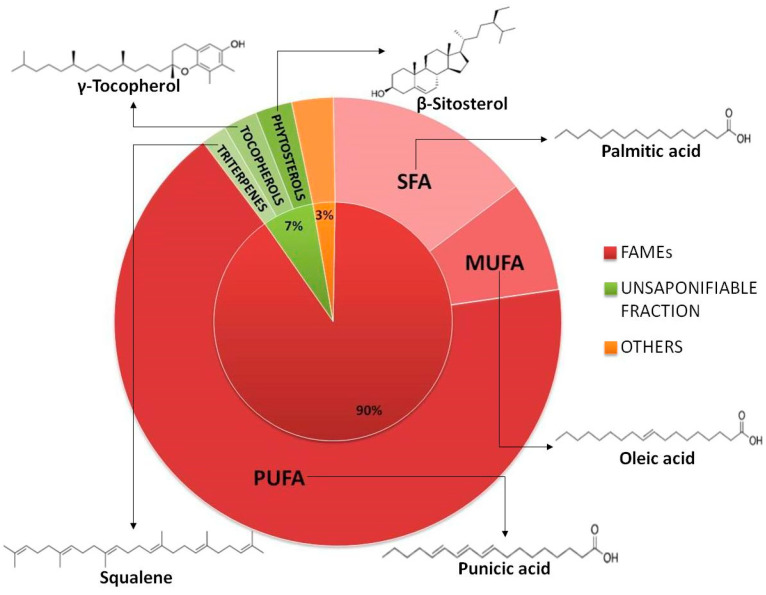
Composition and chemical structures of the most representative biomarkers of PSO.

Pomegranate seed oil (PSO) is the recovery product obtained by the extraction of pomegranate seeds, and its production is, nowadays, the preferred way to valorize the non-edible part of pomegranates [1,2]. As with the pomegranate itself, PSO has recently attracted scientific interest, thanks to its unique composition in terms of both its fatty acid profile and minor bioactive components [3], thus opening the possibility to exploit it in a huge and unexpected range of applications in both human and animal nutrition [4]. In particular, its use as fat substitute in human nutrition, both alone and as ingredient in meat products, has been deeply discussed; additionally, its application as animal feed has been proposed in order to modulate the fat composition in products like eggs, milk and meat. Moreover, the antimicrobial activity of the minor components of PSO has been recently exploited by employing it as a component of active food packaging; finally, the presence of minor secondary metabolites that possess beneficial effects for human health has promoted PSO application as pharmaceutical ingredient in food supplement formulations [5] and references cited therein. PSO represents 10% to 25% of the seed’s total weight. Chemically, PSO composition can be classified in the saponifiable part, mainly represented by polyunsaturated fatty acids (PUFA) and triacylglycerols, where punicic acid (PA) alone counts for 31–86% of the total, and the unsaponifiable part, constituted by minor metabolites, mainly belongings to the families of tocopherols, phytosterols and phenols [1,5]. Figure 1 shows the composition and the chemical structures of the most representative biomarkers of PSO.

Compared to the majority of other commercial edible oils, PSO possess a particularly high proportion of PUFAs, mainly present in the form of triacylglycerol (TAG) [6]. Punicic acid, an ω-5 long chain polyunsaturated fatty acid, is the principal component, thus making PSO a unique edible oil, both in terms of nutritional aspects and beneficial effects on human health. Indeed, it has been deeply demonstrated that PUFAs in general [7], and PA in particular, possess the ability to reduce type II diabetes risk and insulin resistance [8,9], to reduce glucose intake [10], to suppress colon inflammation and carcinogenesis [11,12], to reduce intestinal damage [13] and to enhance the immune response in obesity [14].

Other than PA, the beneficial effects of PSOs are also due to the minor components presents in the unsaponifiable fraction. A synergic role with PUFA and PA, in terms of the modulation of metabolic disorders, is played by phytosterols, particularly β-sitosterol [12,15]. It has been suggested that the level of phytosterols can be an index of PSO genuineness and a marker of the fruit’s variety [1]. Additionally, PSO shows exceptionally high levels of tocopherols, in particular γ-tocopherol, which is, together with PA, a molecule characterizing PSO more than other natural oils [6,16]. It is known that tocopherols play a role as antioxidants both protecting oils from degradation and, after consumption, humans from oxidative stress-related mechanisms [17]. Moreover, PSO exhibits considerably high levels of triterpenes, in particular squalene, which is a dietary supplement able to reduce cholesterol and triglycerides levels. It is primarily extracted from fishes and, in a minor amount, from oleaginous fruits and grains, such as olives, where it is present in an amount comprised between 200 and 12,000 mg/Kg of oil. Finding plants rich in squalene is of importance in vegetarian nutrition, as it allows one to replace animal sources [18]. Finally, pomegranate seeds are also rich of phenols, especially gallic acid [19]. Unfortunately, the high polarity of such molecules gives them high affinity with the juice and hydroalcoholic macerates of pomegranate fruits [20], which results in their being less concentrated in oil.

The quantities of bioactive molecules, both in saponifiable and in unsaponifiable fractions of PSO, depends on several factors, such as the plants cultivar and variety [6,15,21,22,23,24], the period of harvesting [23,25] and the method of extraction [2,26,27]. In particular, cold pressing and supercritical CO_2_ extraction (CO_2_ SFE) are the best extraction methods to preserve thermolabile biomolecules [26]; despite being the most efficient, CO_2_ SFE is still not scalable to an industrial level in terms of safety and costs, so cold pressing is today the method of election to obtain biomarker-enriched PSOs [1,6,26]. The study of the correlation between the active biomolecules of PSO and factors influencing the levels found in oil, has pivotal importance to assess PSO quality in terms of its potential application as functional ingredient. Despite the huge amounts of papers characterizing the different biomarkers present in PSO, only a few comparative studies have been performed to correlate them each other and/or with the endogenous and exogenous factors influencing their relative amounts [6,22,23,24,28]; between them, to the best of our knowledge, just two works, mainly addressed towards detecting oil adulterations, used sufficiently populated groups of samples to infer statistically significant correlations [24,28].

The aim of the present work was to compare 32 commercial cold-pressed PSOs, with different geographical origin, by principal component analysis (PCA) using the PRINCOMP procedure in SAS, to determine components that accounted for most of the total variation from selected metabolites isolated from PSO. Indeed, PCA is an unsupervised clustering technique that can be used to examine the intrinsic variation in a data set and to reduce the dimensionality or complexity of the data [29]. This allowed us to infer statistically significant conclusions with respect to the existence of quality biomarkers, such as the fatty acid profile, useful to assess oil genuineness, and phytosterols, which allows for discrimination between different geographical origins of PSOs.

## 2. Materials and Methods

### 2.1. Sample Collection

Analyses were carried out on 32 samples of commercial cold-pressed pomegranate seed oils from different origins: Italy (8 samples), Europe (2 samples), Turkey (11 samples), South Africa (2 samples), Kenya (3 samples), Iran (2 samples) and India (1 sample). The origins of a few samples could not be established; they were indicated as Unknown (3 samples). All samples were stored in the dark and at room temperature (20–22 °C) until analysis, to prevent any possible matrix alteration.

### 2.2. Chemicals and Reagents

All chemicals and reagents, namely methanol, n-hexane, iso-octane, potassium hydroxide, sodium methoxide, chloroform, ethyl ether, *N*,*O*-bistrimethylsylil-trifluoroacetamide (BSTFA) reagent, sodium carbonate, sodium sulphate and Folin–Ciocalteu reagent, were purchased from Sigma-Aldrich (Milan, Italy). A 0.5 M sodium methoxide solution in methanol and 2.2 N potassium hydroxide solution in ethanol-water (8:2, *v*/*v*) were prepared. Purified water was obtained through a Milli-Q Integral 5 system (Millipore, Merck KGaA, Darmstadt, Germany).

Analytical standards of caffeic acid, cholestanol, tocopherol, (α, γ, and δ) and fatty acid methyl esters mixture (37 FAME mix) were purchased from Sigma-Aldrich Chemical Co. (Milan, Italy). Punicic acid methyl ester analytical standard was purchased by Larodan AB (Solna, Sweden). Working solutions were prepared daily.

### 2.3. Chemical Analysis

#### 2.3.1. Fatty Acid Analysis

Fatty acid methylation was carried out by a base-catalyzed transesterification procedure to avoid isomerization, as reported by Sassano et al. [30]. Briefly, 20 mg of PSO were added into a 12 mL vial with a PTFE cap with 1 mL of 0.5 M sodium methoxide solution. The vial was placed into a water bath and held at 70 °C for 10 min in order to allow complete methylation. At the end of reaction, the vial was cooled, and 4 mL of iso-octane were added under stirring to extract the fatty acid methyl esters (FAMEs). A total of 6 mL of deionized water were added, and the iso-octane layer was collected and dried with Na_2_SO_4_. After centrifugation (4000 rpm, 5 min, T = 24 °C), the organic phase was collected and analyzed. FAMEs were analyzed by gas chromatography coupled with a flame ionization detector (GC-FID, Agilent 6890, Santa Clara, CA, USA) and equipped with a split/splitless injector. FAMEs were separated with an Agilent DB-23 fused silica capillary column (60 m × 0.250 mm × 0.25 μm). Helium was used as carrier gas at flow of 0.9 mL min^−1^. The temperature of injector and detector were 270 °C and 280 °C, respectively; the split ratio was 50:1. The oven temperature program was as follows: initial temperature of 130 °C, held for 1 min, increased from 130 °C to 170 °C at 6.50 °C min^−1^, raised to 215 °C at 2.75 °C min^−1^, held for 12 min, and finally ramped up to 230 °C at 40 °C min^−1^ and held for 8 min. The sample injection volume was 1 μL. The peaks were identified with their retention time using the standard FAME 37 components and an individual standard (punicic acid methyl ester) for the major fatty acid PA. The fatty acids composition was reported as a relative percentage of the total peak area.

#### 2.3.2. Analysis of the Unsaponifiable Fraction by GC-MS Analysis

The unsaponifiable fraction was obtained following the procedure described by Caligiani et al. [18], with some modifications. A sample of 5 g of PSO was dissolved in 100 mL of a 2.2 N potassium hydroxide solution. In order to allow saponification, the sample was boiled and stirred for 1 h at 109 °C by refluxing with the Liebig condenser. The sample was cooled, and 100 mL of distilled water were added. The solution was poured into a separating funnel, and the unsaponifiable fraction was extracted by adding 50 mL of ethyl ether (×4). The organic phase was washed with water until a neutral pH, dried with Na_2_SO_4_, and filtered. The unsaponifiable fraction was obtained by drying with a rotary evaporator at 30 °C. Afterwards, the fraction was dissolved in hexane, and centrifugated (4000 rpm, 5 min, T = 24 °C) for further purification. Supernatant was recovered and stored at −20 °C until analysis.

The total composition of unsaponifiable fraction was analyzed after a silylation reaction, in order to make the compounds more volatile, thermally stable and directly injectable into the GC-MS. Silylation was performed as reported by Caligiani et al. [18] with some adjustments. The unsaponifiable fraction was dissolved in hexane, and silylated with BSTFA for 1.30 h at 65 °C, obtaining a final concentration of 10 mg mL^−1^. Cholestanol standard solution in chloroform was separately silylated with BSTFA for 2.30 h at 65 °C, and it was added as an external standard to the silylated unsaponifiable fraction at a final concentration of 0.2 mg mL^−1^. The composition of the fraction was determined by GC-MS analysis.

GC/MS analyses were performed using a gas chromatograph (Focus GC, Thermo Fisher Scientific, Rodano, Italy) equipped with a Varian VF-5m capillary column (30 m × 0.25 mm × 0.25 μm), coupled to a single quadrupole mass spectrometer (DSQII, Thermo Fisher Scientific). Helium was used as the carrier gas at flow of 0.8 mL min^−1^. The temperature of the injector and transfer-line were 250 °C. The split mode was used for injection with a split ratio of 40:1. The oven temperature program was as follows: initial temperature of 80 °C, held for 2 min, increased from 80 °C to 280 °C at 15 °C min^−1^, held for 20 min, finally raised to 290 °C at 5 °C min^−1^, and held for 5 min. The volume injected was 1 μL. The operating conditions of the MS were the following: ionization potential 70 eV, source temperature 250 °C, solvent delay 16.5 min, mass range 50–500 *m*/*z*. The products were identified by comparison with the NIST database, and quantified with an external standard.

#### 2.3.3. Analysis of Tocopherols by HPLC

Previous works reported that the preliminary extraction methods, used to obtain the unsaponifiable fraction, cause significant loss in the amount of tocopherols [31]. Therefore, tocopherols were separated and quantified by HPLC analysis. Tocopherol extraction was performed following the procedure proposed by Gimeno et al. [31]. A total of 50 mg of PSO sample were dissolved in 950 μL of *n*-hexane and centrifuged (10,000× *g*, 5 min, T = 24 °C). The supernatant was then filtered through a PTFE syringe filter (0.45 μm). Chromatographic separation was performed using Dionex Ultimate 3000 RS (Thermo Fisher Scientific), equipped with an Acclaim Phenyl-1 column (120 Å, 3.0 × 150 mm, 3 μm particle size, Thermo Scientific), with isocratic elution (10% ultrapure water: 90% methanol) at a flow rate of 500 μL min^−1^. The UV/VIS detector was set at 210, 220, 254 and 280 nm. The volume of sample injected was 20 μL. The fluorescence detector was operated at 290 nm (excitation) and 330 nm (emission). According to the regression curve parameters (R^2^) obtained with both detection methods (see Supplementary Material Table S2), tocopherols were quantified using the UV/VIS detection mode. The total run time, including column wash and equilibration, was 30 min. The HPLC system control and data acquisition were performed by Chromeleon software version 7.2 (Thermo Scientific). The tocopherols were identified and quantified using calibration curves in a range between 25 and 500 μg mL^−1^, built from pure reference standards (α-, γ-, and δ-tocopherols) (Table S2).

#### 2.3.4. Total Phenolic Content

Total polyphenols were quantified with Folin–Ciocalteu spectrophotometric assay [32]. Briefly, 5 g of PSO were dissolved in 5 mL of hexane; total polyphenol extraction was performed in a separating funnel with 10 mL of methanol/water (60:40, *v*/*v*) three times. The hydroalcoholic phase was washed with 5 mL of hexane in order to remove any oil residue, then collected in a flask, dried with Na_2_SO_4_, and filtered. The hydroalcoholic phase was evaporated to dryness under vacuum at a maximum temperature of 30 °C with a rotary evaporator. The dry extract was recovered with 5 mL of methanol. A total of 1 mL of extract solution was combined with 5 mL of Folin–Ciocalteu reagent (diluted 1:10) in a 50 mL graduated flask. After 4 min, 4 mL of Na_2_CO_3_ solution 7.5% *w*/*v* was added, mixed vigorously, and left to react for 30 min in the dark. The absorbance at 765 nm was recorded using a UV-visible spectrophotometer (UV/VIS Spectrometer Lambda 35, PerkinElmer, Waltham, MA, USA). A calibration curve of caffeic acid ranging from 25 to 150 μg was used (y = 0.001219x − 0.019881, R^2^ = 0.998). The total phenolic content was determined as mg of caffeic acid equivalents per kg (mg CAE kg^−1^) of the sample.

### 2.4. Data Analysis

PCA using the PRINCOMP procedure in SAS (version 9.4, SAS Institute Inc., Cary, NC, USA) was used to determine components that accounted for most of the total variation from selected biomarkers isolated from commercial cold-pressed pomegranate seed oil. The PCA is an unsupervised clustering technique that can be used to examine the intrinsic variation in a data set, and to reduce the dimensionality or complexity of the data. Score plots were used to highlight similarities and differences among the data sets, and loading vectors revealed which variables (biomarkers) were most responsible for the variation within the principal components (PC) of the data set. After calculation of eigenvalues (the amount of the total variance explained), three scatterplots according to the country of origin in the first three PC scores were created.

Therefore, samples from Italy and Turkey were subjected to an ANOVA using the PROC GLM of SAS. The model included the category of total phytosterol content (High vs. Low) as the fixed effect, whereas the sample was considered as the random effect. Pairwise comparisons were carried out using the LSD test of SAS. Comparisons were discussed at *p* ≤ 0.10, where *p* ≤ 0.05 was considered as significant, whereas 0.10 ≥ *p* ≥ 0.05 was considered as tendency.

## 3. Results

### 3.1. Determination of the Chemical Composition of PSOs

PSO samples analyzed showed similar fatty acid profiles, mostly composed of unsaturated fatty acids. The chemical composition of fatty acids was entirely determined according to the FAME 37 reference standard, but the discussion was limited to the most representative for clarity. FAME analyses demonstrated that PA is the main unsaturated fatty acid observed in PSO. Its concentration in the majority of samples ranged between 48.91 and 82.81%, in line with the results found in the literature, where the reported average amount of PA was 55–86% [6,22,33,34]. Only in five samples (PSO-01, PSO-15, PSO-21, PSO-24 and PSO-31) was PA found at a lower percentage (16.09–38.74%), most of them coming from Italy. In these cases, the entire FAME profile was different from most of the samples, which are detailed in Table 1. Finally, one sample (PSO-11), coming from Turkey, showed a surprisingly low amount of PA, correlated to an unusually high amount of oleic acid, thus originating doubts about its authenticity. Other major fatty acids detected in our PSO samples include palmitic acid, stearic acid, oleic acid, linoleic acid and arachidic acid, whose concentration ranges are in line with several other studies [6,22]. By comparing our GC-FID chromatograms with the literature [35], the peak observed at a retention time of 32.898 min (Figure S1) can be identified as an isomer of punicic acid, as also confirmed by other authors [36]. Table 1 shows the composition of fatty acids reported as relative percentage of peaks area.

The analysis of the unsaponifiable fraction was performed by GC-MS, revealing the presence of three classes of compounds, namely tocopherols, phytosterols and triterpenes. The qualitative profile of the unsaponifiable fraction was similar in all samples. Table 2 reports the quantification of all significant biomarkers identified in the PSO unsaponifiable fractions. Regarding tocopherols observed in GC-MS, their identification and quantification was confirmed by HPLC. The analysis of tocopherol compositions allowed us to identify three isoforms, α-, γ-, and δ-tocopherols, as previously reported in the literature [6,22]. In accordance with previous studies, γ-tocopherol represents the major isoform observed in PSO [6,22,37], ranging between 5.5 and 4073.98 mg kg^−1^ (average 1709.2 mg kg^−1^); the δ-tocopherol concentration ranged between 5.6 and 2361.17 mg kg^−1^ of oil (average 353.09 mg kg^−1^), while only trace amounts of α-tocopherol were detected.

The analysis of the phytosterol fraction revealed that the main phytosterol identified was β-sitosterol, found at concentrations of 444.5–3593.56 mg kg^−1^ (average 1839.69 mg kg^−1^). Campesterol and stigmasterol were identified at lower concentrations, in a range of 53.72–386.92 mg kg^−1^ and 34.43–267.94 mg kg^−1^, respectively, in accordance with Caligiani et al. and Kola et al. [18,38]. As shown in Table S3, squalene and lupeol were the main representative compounds among triterpenes. Total phenol content in cold-pressed PSO was assessed by a spectrophotometric assay (Folin–Ciocalteu) showing a concentration range of 27.13–55.25 mg kg^−1^ (average 36.08 mg kg^−1^). As expected, the analysis demonstrated low values of phenols with a minimal variability among the samples. The results obtained in this study were considerably lower than those observed in other works [39]. These discrepancies could be due to several factors, including the harvesting time, genotypes, cultivation areas, climatic conditions, or different oil extraction methods [4,40].

### 3.2. PCA Analysis

**Figure 2:**
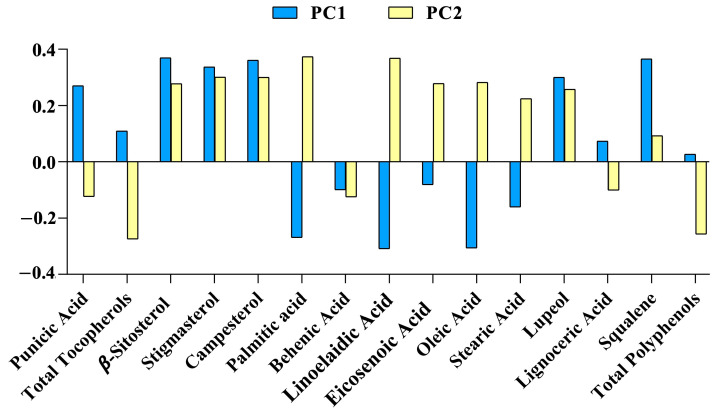
Loading vectors of chemical biomarkers submitted to principal component analysis for principal components (PC) 1 and 2.

Multivariate data analysis was used to achieve data reduction, identifying the major variables contributing to the data variation in the dataset matrix by PCA. PCA allowed for the selection of the bioactive compounds that mostly contributed to samples’ variation. In the PC1, β-sitosterol, stigmasterol, campesterol, squalene, punicic acid and lupeol were positive vectors, whereas palmitic, linoleic and oleic acid were the most negatively associated vectors. In the PC2, β-sitosterol, stigmasterol, campesterol, palmitic acid, linoelaidic acid, eicosenoic acid, oleic acid, stearic acid and lupeol were the most positively associated vectors, whereas total tocopherols and total polyphenols were the most negatively associated vectors (Figure 2).

**Figure 3:**
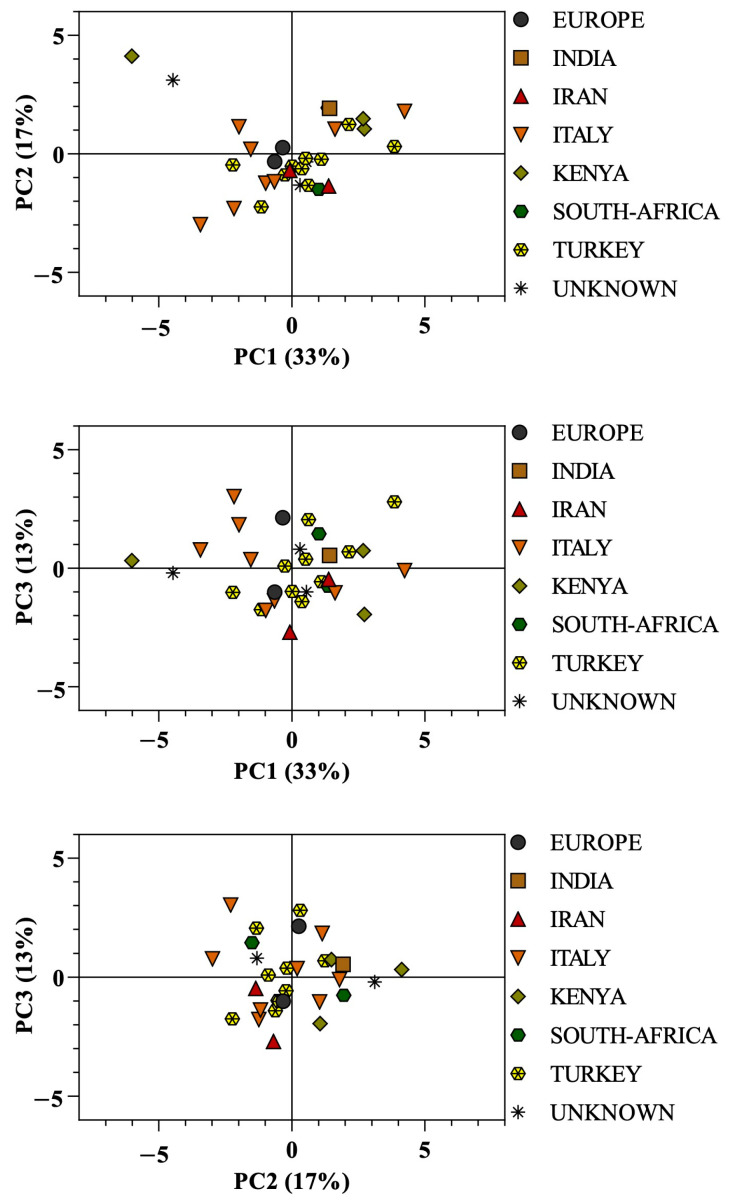
Principal component (PC) analyses in the first three principal components (PC1 vs. PC2, PC1 vs. PC3 and PC2 vs. PC3) on chemical composition of 32 samples of commercial cold-pressed pomegranate seed oils, categorized according to the country of origin: Italy (8 samples), Europe (2 samples), Turkey (11 samples), South Africa (2 samples), Kenya (3 samples), Iran (2 samples), India (1 sample), and Unknown (3 samples). Three scatterplots according to the country of origin in the first three PC scores were created.

After data reduction, PCA resulted in the first three PCs in the data matrix, which explained 63% of total variance, even though the total variance explained by the first three PCs was not high. However, this analysis did not allow a preliminary discrimination of PSOs by geographical origin (Figure 3), considering all samples collected.

**Figure 4:**
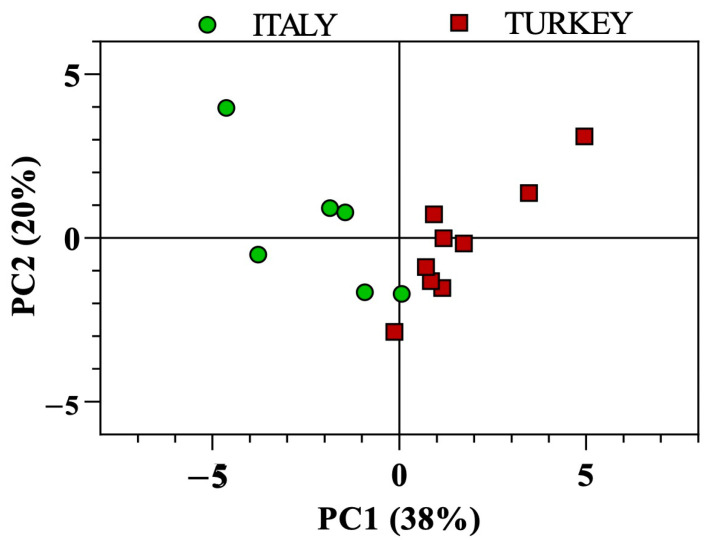
Principal component (PC) analyses in the first two principal components on chemical composition of 15 samples of commercial cold-pressed pomegranate seed oils, categorized according to the country of origin: Italy (6 samples) and Turkey (9 samples).

When the dataset was reduced only to samples from Italy and Turkey (several samples for each country), the PCA analysis was able to well discriminate samples according to origin country. The PC1 explained the 38% of the variance, whereas the PC2 was able to explain 20% of the total variance (Figure 4). Even in this case, the percentage reached with the first 2 PC was only relatively high.

**Figure 5:**
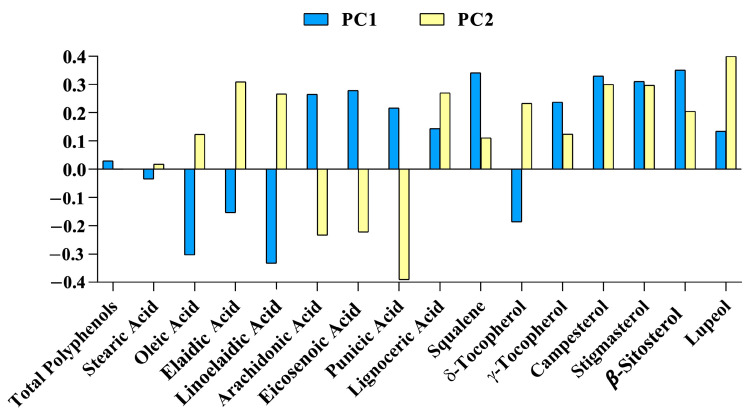
Loading vectors of chemical biomarkers submitted to principal component analysis, with samples from only Italy and Turkey for principal components (PC) 1 and 2, respectively.

From the multivariate analysis, the PC1 was the best component to cluster samples from Italy versus samples from Turkey. Evaluating the loading vectors of PC1 to discriminate samples from Italy and Turkey, arachidonic acid, eicosenoic acid, punicic acid, γ-tocopherol, campesterol, stigmasterol and β-sitosterol were the most positively associated vectors, whereas oleic acid, linoleic acid and elaidic acid were the most negatively associated vectors (Figure 5).

In fact, samples from Italy were characterized by a greater content of palmitic acid, oleic acid, linoelaidic acid, and δ-tocopherol; however, a lower content of punicic acid (tendency), campesterol, squalene, and β-sitosterol (*p* < 0.05; Table 3) was observed.

## 4. Discussion

The chemical composition of PSO is responsible for its unique nutraceutical profile. According to the literature, the fatty acid composition of PSOs is represented by almost 80% of PUFAs, with 70% constituted by PA, 15% by monounsaturated fatty acids (MUFAs), essentially oleic acid, and 5% by saturated fatty acids (SFAs). Less clear is the influence exerted on the fatty acid profile by plant variety and cultivar, factors connected to geographical origin of the seeds [6,15,21,22,23,24,33,34,38].

The fatty acid TAG composition is responsible for the PSO’s ability to positively modulate glucose intake [10], to control inflammatory diseases connected to obesity [12,14], to modulate insulin resistance [9], to exert anti nociceptive functions [41], to repair intestinal damage [13] and to control colon carcinogenesis [11].

Concerning the composition of minor metabolites present in the unsaponifiable fraction, the literature reports unusual levels of tocopherols, polyphenols and phytosterols in respect to other natural oils [6]. The dependence of the levels of such metabolites from the extraction procedure is clear: they are better preserved using low temperature extracting methods, such as cold pressing and CO_2_ SFE [1,6,26], while it is controversial whether the dependence is on the plant variety or the geographical origin.

Our statistical comparative study was conducted on 32 commercial cold-pressed PSOs, thus eliminating the variability connected to the extraction method, characterized by the different geographical origins of the seeds.

Firstly, the main chemical components of the complete set of 32 samples, determined both from saponifiable and unsaponifiable fractions, were compared by PCA analysis, regardless of the geographical origin of the seeds. The aim was to discover which biomolecules most influenced the sample discrimination. As is shown in Figure 2, PA and phytosterols positively influenced the PCs; on the contrary, the other major fatty acids, such as oleic, palmitic and linoelaidic acid, influenced them negatively. This tendency is in accordance with previous studies on the fatty acids profile of PSOs from different countries and seed varieties [21,25,33]. In particular, Zaouay et al. observed that, regardless of the variety and the origin of seeds, fatty acid profiles changed during maturation; in particular, a decrease in linoelaidic acid amount corresponds to an increase in palmitic and linoleic acids. Moreover, the amount of oleic acids increase with ripening, due to the oleate desaturase activation, which is able to transform linoelaidic acid into oleic acid.

In our study, after reduction to three PCs, the data belonging to the 32 PSO samples were associated with their geographical origin. PCA was not able to discriminate samples on the base of their origin (Figure 3). This result was in accordance with the study of Elfalleh et al. [21], who compared the fatty acid profile of Tunisian and Chinese PSOs by PCA analysis, without obtaining a significative discrimination between the two geographical sets of samples.

Nevertheless, it is worth noting that our sample set was non-homogeneous in terms of origin representativity, being composed of both well-represented countries, such as Italy and Turkey, and scarcely-represented countries such as South Africa, Kenya and Iran.

For this reason, we decided to limit the statistical analyses to the two most represented countries, by selecting a data subset of 15 samples, 9 from Turkey and 6 from Italy. As reported in Figure 4, the PCA on the reduced subset of samples was able to discriminate the samples in two well-differentiated groups, especially on the base of the PC1; the most important compounds with positive values were phytosterols, punicic acid to a lesser extent and, newly, γ-tocopherols, as reported in Figure 5. On the contrary, PC1 was negatively influenced by the oleic and linoleic acid content, which had the most negative values (Figure 5). These results, confirming the importance of having a significative and homogeneous data set to infer a statistical correlation with the origin, supported the hypothesis that phytosterol, besides fatty acid profiles, can be fundamental for discrimination.

An ANOVA analysis, to understand which biomarkers were specifically responsible for the differentiation between the two PSO groups, was then performed. Results reported in Table 3 clarified that the profile of minor fatty acids, such as oleic and linoleic acid, significatively discriminates the two groups of samples, while the amount of PA had only a statistic tendence to differ. Moreover, it has been assessed that the oils with a higher content of PA, namely Turkish oils, showed a lower content of oleic, linoleic and palmitic acids. As it has been inferred, the inverse correlation between PA and oleic, palmitic and linoleic acids can be due to the maturation stage of the fruits more than the varietal origin [25]; thus, the differentiation between Turkish and Italian PSOs can be ascribed to different pedo-climatic conditions in fruit cultivation and/or harvesting between the two countries, with Italian seeds being processed in a more advanced maturation stage relative to Turkish ones.

Interestingly, Turkish PSOs were also characterized by significatively higher levels of phytosterols relative to the Italian PSOs.

Phytosterols, especially β-sitosterol, which is the most present among the family, are the secondary metabolites that, according to the literature, exert beneficial effects similar to PA. In particular some studies attributed the ability of phytosterols to lower cholesterol, triglycerides and low-density lipoproteins, both in vitro and in vivo models of hypercholesterolemia [15]; in both clinical and animal studies on obesity-related chronic inflammation, negative correlations between the serum sitosterol level, the serum IL-6 and the TNF-α levels in both diabetic subjects and non-diabetic subjects has been found [14]. FDA and EFSA have approved the use of plant sterols in functional foods with an intake of 3 g/day, for two or three weeks for the lowering effect on cholesterol levels [42]. Moreover, it has been suggested that the augmented efficiency of PSO to inhibit TNF α-induced neutrophil hyperactivation, and to protect from colon inflammation in in vivo experiments, relative to pure PA, could be explained by inferring an additive and/or synergistic effect with other minor components, such as phytosterols [12].

Our results confirmed the preponderance of β-sitosterol among the phytosterol family, composed also of stigmasterol, campesterol, and lupeol. Additionally, data confirmed that phytosterol content can statistically discriminate PSOs of different origins. Indeed, results reported in Table S3 clarified that phytosterols, and their precursor squalene, significatively allowed distinguishing between the PSOs country of origin, thus definitively assessing the importance of the phytosterols as quality biomarkers of PSOs.

On the contrary, tocopherols variability inside the groups was too high to make their variability significant between the two groups. Consequently, the amount of γ-tocopherol, the most represented among the family, was not useful to discriminate either the quality, or the origin of the oils. It is worth noting that the *p* value < 0.05 for δ-tocopherol can be an artefact, because it was a minor biomarker, often determined on the detection limit of the HPLC analysis, and its limited variability inside the groups should be due to a limit of the technique. The same can be concluded about the statistical variance in terms of phenols.

## 5. Conclusions

In this study, the chemical composition of 32 commercial cold-pressed PSOs was statistically compared to the geographical origins of the seeds using PCA. The analysis was significative only when using a reduced subset of samples where the geographical origin was well represented, namely composed of nine samples from Turkey and six samples from Italy. Such a subset of samples was further subjected to variance analysis by an ANOVA test. Regardless of the varietal difference between seeds, PSOs coming from Turkey and Italy displayed a significative difference in terms of both fatty acid profile and phytosterol content, while they did not differ by the amounts of tocopherols and polyphenols. In particular, the Turkish PSOs were found to be of higher quality relative to the Italian ones.

Taking into account the synergistic beneficial effects of PUFAs and sterols, our results appear of fundamental importance, as they support the proposition of total phytosterols, in particular β-sitosterol, as discriminating quality markers in a population of PSOs having different geographical origins, together with their fatty acid profile.

## Figures and Tables

**Table 1:** Composition of fatty acids (FAMEs) * reported as relative percentages of peaks area (%).

Sample	Origin	Palmitic Acid	Heptadecanoic Acid	Stearic Acid	Oleic Acid	Elaidic Acid	Unknown	Linoleic Acid	Linoelaidic Acid	Arachidic Acid	Eicosenoic Acid	Punicic Acid	Behenic Acid	Erucic Acid	Punicic Acid Isomer	Lignoceric Acid
PSO-01	Italy	3.20 ± 0.05	0.06 ± .00	1.59 ± 0.15	6.69 ± 0.43	0.47 ± 0.03	0.00 ± 0.00	6.03 ± 0.22	0.04 ± 0.01	0.44 ± 0.01	0.48 ± 0.19	34.43 ± 1.87	16.66 ± 0.83	16.85 ± 0.37	11.15 ± 0.39	1.40 ± 0.98
PSO-02	Italy	2.74 ± 0.06	0.23 ± 0.29	2.02 ± 0.04	4.93 ± 0.21	0.15 ± 0.25	0.00 ± 0.00	4.65 ± 0.11	0.01 ± 0.02	0.48 ± 0.02	0.42 ± 0.15	79.69 ± 0.49	0.00 ± 0.00	3.30 ± 0.27	0.99 ± 0.05	0.39 ± 0.12
PSO-03	Turkey	2.82 ± 0.12	0.02 ± 0.03	2.03 ± 0.08	4.45 ± 0.24	0.00 ± 0.00	0.00 ± 0.00	4.59 ± 0.13	0.00 ± 0.00	0.50 ± 0.03	0.81 ± 0.01	77.23 ± 0.51	0.00 ± 0.00	5.73 ± 0.19	0.91 ± 0.07	0.58 ± 0.05
PSO-04	South Africa	3.48 ± 0.02	0.04 ± 0.04	1.55 ± 0.29	5.98 ± 0.48	0.00 ± 0.00	0.55 ± 0.03	6.41 ± 0.05	0.03 ± 0.03	4.19 ± 3.14	2.77 ± 0.66	74.57 ± 0.63	0.00 ± 0.00	3.65 ± 0.02	0.59 ± 0.01	0.84 ± 0.07
PSO-05	Turkey	2.75 ± 0.13	0.00 ± 0.00	1.86 ± 0.11	4.46 ± 0.23	0.00 ± 0.00	0.31 ± 0.00	4.72 ± 0.25	0.00 ± 0.00	0.55 ± 0.13	0.74 ± 0.14	56.79 ± 1.69	10.61 ± 0.52	10.97 ± 0.47	2.61 ± 0.06	3.63 ± 1.10
PSO-06	India	3.22 ± 0.11	0.03 ± 0.01	2.22 ± 0.46	6.25 ± 0.25	0.00 ± 0.00	0.00 ± 0.00	6.06 ± 0.17	0.02 ± 0.01	0.52 ± 0.02	0.83 ± 0.05	48.91 ± 0.79	12.86 ± 0.45	12.62 ± 0.31	6.39 ± 0.23	0.06 ± 0.01
PSO-07	Kenya	2.71 ± 0.10	0.04 ± 0.02	1.93 ± 0.06	4.24 ± 0.01	0.23 ± 0.03	0.00 ± 0.00	4.35 ± 0.10	0.01 ± 0.01	0.49 ± 0.01	0.83 ± 0.03	81.57 ± 0.78	0.00 ± 0.00	2.73 ± 0.20	0.72 ± 0.07	0.12 ± 0.06
PSO-08	Unknown	4.42 ± 0.12	0.05 ± 0.00	2.61 ± 0.02	16.82 ± 0.25	0.00 ± 0.00	0.00 ± 0.00	26.22 ± 0.28	0.02 ± 0.01	0.39 ± 0.01	0.35 ± 0.06	38.74 ± 0.53	4.41 ± 0.15	4.52 ± 0.42	1.22 ± 0.03	0.13 ± 0.02
PSO-09	Turkey	2.86 ± 0.10	0.09 ± 0.02	1.97 ± 0.06	4.73 ± 0.03	0.00 ± 0.00	0.00 ± 0.00	4.64 ± 0.10	0.01 ± 0.01	0.47 ± 0.02	0.92 ± 0.03	67.66 ± 0.94	7.25 ± 0.66	6.22 ± 0.05	3.03 ± 0.08	0.15 ± 0.01
PSO-10	Turkey	2.74 ± 0.25	0.02 ± 0.01	1.98 ± 0.03	4.58 ± 0.15	0.00 ± 0.00	0.00 ± 0.00	4.98 ± 0.13	0.00 ± 0.00	0.48 ± 0.01	0.77 ± 0.01	74.24 ± 5.46	2.28 ± 0.94	5.39 ± 0.13	1.22 ± 0.30	0.06 ± 0.02
PSO-11	Turkey	7.10 ± 0.19	0.00 ± 0.00	3.10 ± 0.07	48.00 ± 0.54	0.00 ± 0.00	0.00 ± 0.00	21.80 ± 0.30	0.30 ± 0.18	0.60 ± 0.01	11.90 ± 0.27	0.50 ± 0.02	0.90 ± 0.01	2.44 ± 0.15	0.00 ± 0.00	0.20 ± 0.02
PSO-12	Turkey	2.78 ± 0.07	0.02 ± 0.01	2.40 ± 0.89	4.57 ± 0.13	0.00 ± 0.00	0.00 ± 0.00	4.66 ± 0.08	0.00 ± 0.00	0.46 ± 0.02	0.73 ± 0.03	53.73 ± 1.34	12.95 ± 0.43	13.33 ± 0.37	4.26 ± 0.19	0.05 ± 0.00
PSO-13	Iran	2.67 ± 0.13	0.03 ± 0.01	2.08 ± 0.16	4.86 ± 0.12	0.00 ± 0.00	0.00 ± 0.00	4.80 ± 0.10	0.01 ± 0.01	0.48 ± 0.01	0.73 ± 0.02	82.81 ± 0.93	0.00 ± 0.00	1.00 ± 0.03	0.36 ± 0.07	0.04 ± 0.02
PSO-14	Italy	2.87 ± 0.14	0.05 ± 0.00	2.07 ± 0.08	5.83 ± 0.34	0.00 ± 0.00	0.00 ± 0.00	5.65 ± 0.18	0.01 ± 0.01	0.45 ± 0.00	0.62 ± 0.07	78.30 ± 0.74	0.00 ± 0.00	3.83 ± 0.36	0.40 ± 0.07	0.08 ± 0.03
PSO-15	Italy	3.12 ± 0.08	0.10 ± 0.02	2.30 ± 0.08	6.60 ± 0.58	0.49 ± 0.02	0.00 ± 0.00	6.22 ± 0.09	0.00 ± 0.00	0.50 ± 0.05	0.81 ± 0.07	25.80 ± 0.69	19.54 ± 0.82	19.03 ± 0.66	14.27 ± 0.45	1.10 ± 0.85
PSO-16	Italy	2.72 ± 0.12	0.04 ± 0.02	2.08 ± 0.25	5.03 ± 0.21	0.00 ± 0.00	0.00 ± 0.00	5.27 ± 0.24	0.02 ± 0.01	0.52 ± 0.09	0.75 ± 0.03	69.66 ± 4.90	3.97 ± 0.94	8.33 ± 0.09	1.01 ± 0.07	0.59 ± 0.08
PSO-17	Turkey	2.68 ± 0.06	0.02 ± 0.01	1.97 ± 0.08	4.23 ± 0.11	0.45 ± 0.02	0.00 ± 0.00	4.48 ± 0.12	0.00 ± 0.00	0.51 ± 0.02	0.81 ± 0.02	70.78 ± 0.67	6.57 ± 0.27	6.03 ± 0.13	0.81 ± 0.01	0.42 ± 0.01
PSO-18	South Africa	3.34 ± 0.05	0.06 ± 0.01	0.65 ± 0.05	5.46 ± 0.14	0.30 ± 0.26	0.45 ± 0.04	6.48 ± 0.16	0.05 ± 0.02	0.55 ± 0.03	0.66 ± 0.01	70.41 ± 4.13	3.89 ± 0.05	4.80 ± 0.16	1.25 ± 0.04	0.27 ± 0.14
PSO-19	Unknown	2.79 ± 0.08	0.04 ± 0.01	1.94 ± 0.07	5.00 ± 0.39	0.46 ± 0.02	0.13 ± 0.02	4.79 ± 0.06	1.61 ± 0.79	0.50 ± 0.03	0.77 ± 0.02	72.70 ± 0.31	5.71 ± 0.64	3.44 ± 0.4	0.35 ± 0.16	0.45 ± 0.04
PSO-20	Turkey	2.74 ± 0.13	0.02 ± 0.01	2.66 ± 0.67	6.06 ± 0.44	0.46 ± 0.04	0.26 ± 0.01	5.04 ± 0.16	0.00 ± 0.00	0.53 ± 0.03	0.69 ± 0.02	62.07 ± 3.38	7.73 ± 0.30	8.22 ± 0.23	1.27 ± 0.03	0.77 ± 0.07
PSO-21	Kenya	4.35 ± 0.27	0.02 ± 0.01	2.82 ± 0.08	19.95 ± 0.65	0.00 ± 0.00	0.15 ± 0.05	30.89 ± 1.01	0.00 ± 0.00	0.41 ± 0.04	2.94 ± 0.26	19.96 ± 1.40	8.18 ± 0.08	7.10 ± 0.12	3.24 ± 0.13	0.74 ± 0.10
PSO-22	Europe	3.01 ± 0.04	0.00 ± 0.00	2.21 ± 0.21	6.44 ± 0.25	0.30 ± 0.06	0.27 ± 0.02	6.45 ± 0.05	0.00 ± 0.00	0.47 ± 0.02	0.73 ± 0.02	71.8 ± 1.46	4.50 ± 0.37	2.37 ± 0.07	0.48 ± 0.09	0.82 ± 0.08
PSO-23	Kenya	2.92 ± 0.07	0.24 ± 0.01	2.36 ± 0.41	6.01 ± 0.11	0.29 ± 0.05	0.45 ± 0.12	6.33 ± 0.10	0.48 ± 0.02	0.50 ± 0.08	0.28 ± 0.02	73.23 ± 1.17	3.63 ± 0.31	2.10 ± 0.08	0.42 ± 0.08	0.41 ± 0.07
PSO-24	Italy	4.19 ± 0.08	0.09 ± 0.01	1.91 ± 0.35	8.72 ± 0.27	0.55 ± 0.02	0.52 ± 0.01	9.16 ± 0.08	0.23 ± 0.01	0.13 ± 0.00	0.37 ± 0.03	16.09 ± 2.91	14.76 ± 2.50	18.01 ± 0.38	21.85 ± 0.51	0.21 ± 0.01
PSO-25	Turkey	2.99 ± 0.07	0.06 ± 0.00	3.34 ± 0.16	5.6 ± 0.23	0.39 ± 0.02	0.31 ± 0.03	6.49 ± 0.19	0.30 ± 0.07	0.43 ± 0.01	0.45 ± 0.06	54.20 ± 2.80	8.76 ± 0.65	8.98 ± 0.34	5.54 ± 0.39	0.80 ± 0.06
PSO-26	Turkey	2.62 ± 0.89	0.00 ± 0.00	1.83 ± 0.02	5.02 ± 1.36	0.09 ± 0.01	0.38 ± 0.08	4.64 ± 0.72	0.08 ± 0.01	0.41 ± 0.08	0.67 ± 0.01	57.65 ± 3.98	22.71 ± 2.49	3.99 ± 0.15	0.00 ± 0.00	0.17 ± 0.01
PSO-27	Iran	2.13 ± 0.99	0.24 ± 0.01	1.82 ± 0.16	6.63 ± 0.71	0.48 ± 0.05	0.27 ± 0.02	4.28 ± 0.41	0.10 ± 0.09	0.55 ± 0.02	0.18 ± 0.07	64.47 ± 8.07	4.18 ± 0.09	7.12 ± 3.5	0.52 ± 0.15	7.1 ± 1.89
PSO-28	Italy	3.47 ± 0.29	0.04 ± 0.01	2.73 ± 0.04	8.42 ± 1.52	0.36 ± 0.02	0.00 ± 0.00	6.70 ± 0.22	0.02 ± 0.01	0.48 ± 0.02	0.66 ± 0.01	51.21 ± 1.54	10.58 ± 1.46	11.40 ± 1.13	3.52 ± 0.29	0.83 ± 0.01
PSO-29	Turkey	3.18 ± 0.23	0.00 ± 0.00	2.44 ± 0.38	0.00 ± 0.00	0.56 ± 0.01	0.00 ± 0.00	3.93 ± 0.93	0.08 ± 0.02	0.52 ± 0.07	0.77 ± 0.04	51.54 ± 0.52	13.61 ± 0.88	14.93 ± 0.14	5.45 ± 0.05	0.90 ± 0.08
PSO-30	Unknown	2.72 ± 0.05	0.05 ± 0.01	1.86 ± 0.03	5.13 ± 0.11	0.44 ± 0.01	0.25 ± 0.01	5.17 ± 0.09	0.66 ± 0.08	0.57 ± 0.07	0.84 ± 0.08	57.37 ± 0.89	11.55 ± 1.02	10.59 ± 0.71	2.30 ± 0.10	0.21 ± 0.09
PSO-31	Europe	3.67 ± 0.13	0.00 ± 0.00	2.48 ± 0.29	0.00 ± 0.00	0.73 ± 0.05	0.35 ± 0.00	6.70 ± 0.78	0.00 ± 0.00	0.51 ± 0.02	0.80 ± 0.04	26.38 ± 3.28	20.27 ± 1.89	19.40 ± 0.09	16.22 ± 0.08	0.73 ± 0.07
PSO-32	Italy	3.33 ± 0.15	0.00 ± 0.00	2.35 ± 0.40	0.56 ± 0.08	5.03 ± 0.84	0.30 ± 0.00	0.34 ± 0.06	0.14 ± 0.04	0.90 ± 0.14	1.89 ± 0.39	64.61 ± 5.80	0.34 ± 0.06	10.01 ± 0.00	8.53 ± 0.71	0.57 ± 0.09

* Data expressed as means and standards deviations (SD) of three independent observations.

**Table 2:** Composition of unsaponifiable fraction and total phenol content reported as mg kg^−1^.

Sample	Origin	Unsaponifiable Fraction (mg kg^−1^) *								Total Phenols Content (mg kg^−1^) *
		Squalene	δ-Tocopherol	γ-Tocopherol	Total Tocopherols	Campesterol	Stigmasterol	β-Sitosterol	Lupeol	
PSO-01	Italy	393.09 ± 13.19	1203.11 ± 16.69	1252.21 ± 12.60	2455.32 ± 28.88	53.72 ± 3.74	34.43 ± 2.80	444.50 ± 27.04	60.17 ± 5.34	40.24 ± 1.36
PSO-02	Italy	2335.28 ± 125.74	5.60 ± 0.04	2724.59 ± 16.06	2730.19 ± 16.08	362.39 ± 26.77	211.22 ± 11.32	3593.56 ± 133.63	570.85 ± 11.14	32.12 ± 1.10
PSO-03	Turkey	1317.17 ± 83.15	138.31 ± 1.59	1037.86 ± 13.77	1176.16 ± 12.46	102.29 ± 9.32	47.31 ± 3.91	1024.42 ± 36.18	145.32 ± 8.76	46.93 ± 1.79
PSO-04	South Africa	1706.28 ± 113.58	5.60 ± 0.02	2059.59 ± 17.30	2065.19 ± 17.28	326.79 ± 3.79	180.08 ± 1.77	2492.22 ± 40.20	263.12 ± 10.07	33.67 ± 3.66
PSO-05	Turkey	2307.33 ± 97.53	706.19 ± 4.37	4073.98 ± 18.01	4780.17 ± 17.18	386.92 ± 22.97	212.69 ± 7.19	3387.80 ± 127.56	431.45 ± 21.84	44.99 ± 2.17
PSO-06	India	1876.21 ± 68.73	5.60 ± 0.10	1401.02 ± 8.97	1406.62 ± 9.00	302.25 ± 12.84	215.52 ± 6.48	2987.92 ± 104.18	301.61 ± 9.32	29.03 ± 1.06
PSO-07	Kenya	3100.19 ± 114.88	553.60 ± 13.89	259.66 ± 4.15	813.25 ± 17.90	284.23 ± 21.30	187.12 ± 9.12	2447.84 ± 33.52	302.97 ± 0.58	28.51 ± 2.37
PSO-08	Unknown	0.00 ± 0.00	397.77 ± 4.88	5.50 ± 0.04	403.27 ± 4.88	196.42 ± 14.79	130.05 ± 4.31	1449.37 ± 7.05	154.90 ± 9.39	29.39 ± 0.94
PSO-09	Turkey	1387.66 ± 53.5	5.60 ± 0.04	1623.98 ± 8.76	1629.58 ± 8.79	185.74 ± 2.32	119.43 ± 5.70	1710.55 ± 87.97	240.70 ± 18.08	32.34 ± 1.39
PSO-10	Turkey	1457.68 ± 24.07	373.94 ± 5.90	1272.86 ± 2.76	1646.80 ± 8.46	190.70 ± 5.05	133.39 ± 3.54	1767.26 ± 26.11	214.19 ± 6.06	34.57 ± 1.41
PSO-11	Turkey	6012.66 ± 103.55	5.60 ± 0.10	5.50 ± 0.02	11.10 ± 0.09	673.51 ± 29.30	168.17 ± 17.03	1830.86 ± 25.79	0.00 ± 0.00	43.28 ± 2.38
PSO-12	Turkey	1652.92 ± 45.76	415.77 ± 2.97	2440.52 ± 10.52	2856.29 ± 13.42	190.99 ± 4.56	124.83 ± 1.57	1620.30 ± 31.32	164.64 ± 5.60	32.87 ± 1.11
PSO-13	Iran	1471.22 ± 12.98	1103.59 ± 15.84	5.50 ± 0.06	1109.09 ± 15.84	171.59 ± 2.00	110.82 ± 5.61	1616.48 ± 33.39	129.84 ± 1.79	27.21 ± 2.23
PSO-14	Italy	430.49 ± 34.83	395.63 ± 11.46	1798.55 ± 7.08	2194.19 ± 18.18	163.47 ± 20.60	83.63 ± 9.21	1464.50 ± 46.69	162.96 ± 18.92	33.07 ± 1.08
PSO-15	Italy	530.42 ± 18.34	2361.17 ± 20.42	3277.82 ± 25.50	5638.99 ± 45.21	142.92 ± 1.79	107.27 ± 1.83	903.20 ± 14.93	134.08 ± 12.61	45.00 ± 2.65
PSO-16	Italy	1091.34 ± 32.65	5.60 ± 0.08	1288.62 ± 4.19	1294.22 ± 4.15	143.97 ± 17.90	99.51 ± 1.08	1240.41 ± 75.45	226.18 ± 4.81	36.39 ± 2.49
PSO-17	Turkey	1406.10 ± 15.56	5.60 ± 0.09	1755.98 ± 7.83	1761.58 ± 7.91	240.10 ± 5.79	156.78 ± 2.98	2068.43 ± 46.74	299.12 ± 13.77	35.42 ± 1.82
PSO-18	South Africa	987.06 ± 43.90	5.60 ± 0.13	3730.73 ± 48.02	3736.33 ± 47.97	269.02 ± 16.14	150.32 ± 8.43	1954.02 ± 23.28	256.35 ± 13.80	55.25 ± 2.41
PSO-19	Unknown	1217.13 ± 62.33	474.79 ± 6.11	646.92 ± 10.92	1121.70 ± 16.63	244.31 ± 13.14	121.05 ± 15.84	2205.95 ± 100.48	224.23 ± 29.15	36.83 ± 1.18
PSO-20	Turkey	1043.83 ± 22.71	466.24 ± 2.28	2491.26 ± 4.40	2957.49 ± 6.59	257.94 ± 4.94	141.74 ± 0.96	2207.17 ± 15.05	288.80 ± 7.30	39.32 ± 1.16
PSO-21	Kenya	567.80 ± 26.71	138.54 ± 1.32	485.09 ± 4.96	623.63 ± 6.13	137.38 ± 7.23	96.26 ± 4.47	1098.91 ± 23.56	134.62 ± 6.63	33.81 ± 1.85
PSO-22	Europe	657.50 ± 15.28	5.60 ± 0.05	1987.06 ± 14.17	1992.66 ± 14.12	167.18 ± 2.63	142.85 ± 1.88	1303.23 ± 91.29	268.91 ± 11.37	30.61 ± 0.30
PSO-23	Kenya	1253.30 ± 39.80	5.60 ± 0.10	1687.32 ± 17.09	1692.92 ± 17.04	326.83 ± 20.23	267.94 ± 5.63	2665.55 ± 97.89	511.02 ± 36.49	45.05 ± 0.57
PSO-24	Italy	961.61 ± 27.74	1122.21 ± 1.25	5.50 ± 0.08	1127.71 ± 1.21	174.45 ± 6.85	114.23 ± 0.57	1090.96 ± 24.28	497.25 ± 29.04	34.57 ± 1.20
PSO-25	Turkey	421.40 ± 17.81	5.60 ± 0.11	774.92 ± 3.51	780.52 ± 3.57	142.48 ± 1.41	100.52 ± 8.62	1082.06 ± 14.01	165.75 ± 10.92	33.77 ± 1.52
PSO-26	Turkey	1847.25 ± 19.08	5.60 ± 0.02	2138.98 ± 6.68	2144.58 ± 6.67	207.03 ± 8.67	167.31 ± 2.79	1722.72 ± 36.86	277.08 ± 10.09	47.36 ± 3.86
PSO-27	Iran	1648.06 ± 56.88	5.60 ± 0.09	2377.52 ± 7.96	2383.12 ± 7.98	211.72 ± 9.02	172.91 ± 8.47	1558.15 ± 21.08	246.65 ± 9.00	27.13 ± 0.85
PSO-28	Italy	610.02 ± 11.43	699.48 ± 4.10	2135.13 ± 28.40	2834.61 ± 32.43	170.24 ± 7.69	111.79 ± 9.60	1574.36 ± 50.78	289.59 ± 19.19	30.42 ± 2.31
PSO-29	Turkey	2198.97 ± 34.28	5.60 ± 0.12	1692.20 ± 7.96	1697.80 ± 7.96	311.87 ± 4.86	228.33 ± 10.36	2137.97 ± 30.92	384.86 ± 6.21	28.90 ± 1.31
PSO-30	Unknown	1634.28 ± 42.28	5.60 ± 0.04	3755.44 ± 12.94	3761.04 ± 12.93	172.69 ± 6.96	142.51 ± 7.78	1698.60 ± 25.69	238.13 ± 8.69	39.46 ± 1.00
PSO-31	Europe	1272.72 ± 40.68	5.60 ± 0.03	1953.52 ± 33.14	1959.12 ± 33.12	205.73 ± 14.85	177.88 ± 9.53	2165.21 ± 47.21	237.76 ± 10.44	39.27 ± 1.20
PSO-32	Italy	2091.87 ± 26.60	311.36 ± 4.28	845.64 ± 18.00	1156.99 ± 21.26	236.49 ± 19.69	175.39 ± 6.88	2346.67 ± 79.89	323.61 ± 20.75	35.03 ± 2.81

* Data expressed as means and standards deviations (SD) of three independent observations.

**Table 3:** Chemical compositions of 15 samples of commercial cold-pressed pomegranate seed oil, categorized according to the country of origin: Italy (6 samples) and Turkey (9 samples.).

Item	Italy	Turkey	SEM ^1^	*p*-Value
Palmitic Acid (%)	3.26	2.81	0.14	<0.05
Heptadecanoic Acid (%)	0.08	0.03	0.01	0.12
Stearic Acid (%)	2.11	2.25	0.12	0.56
Oleic Acid (%)	6.88	4.37	0.93	<0.05
Elaidic Acid (%)	0.31	0.20	0.09	0.37
Unknown 1 (%)	0.26	0.16	0.03	0.23
Linoleic Acid (%)	6.50	4.82	0.40	<0.05
Linoelaidic Acid (%)	0.05	0.05	0.01	0.13
Arachidic Acid (%)	0.42	0.49	0.03	0.19
Eicosenoic Acid (%)	0.61	0.73	0.05	0.10
Punicic Acid (%)	45.91	62.60	4.86	0.07
Behenic Acid (%)	10.91	9.74	2.40	0.77
Erucic Acid (%)	12.91	8.38	1.35	0.46
Punicic acid isomer (%)	8.73	2.51	0.98	0.78
Lignoceric Acid (%)	0.70	0.75	0.36	0.91
Total Polyphenols (mg kg^−1^)	36.62	37.65	2.45	0.75
δ-Tocopherols (mg kg^−1^)	964.53	212.84	130.55	<0.05
Stigmasterol (mg kg^−1^)	91.81	143.23	10.30	0.70
Campesterol (mg kg^−1^)	141.46	221.61	29.57	<0.05
Lupeol (mg kg^−1^)	228.37	261.19	37.76	0.60
Squalene (mg kg^−1^)	669.50	1504.03	144.77	<0.05
γ-Tocopherol (mg kg^−1^)	1626.31	1930.25	406.14	0.56
β-Sitosterol (mg kg^−1^)	1119.65	1872.87	239.82	<0.05

^1^ Greatest standard error of the mean.

## Data Availability

The data presented in this study are available on request from the corresponding author.

## Supplementary Materials

The following supporting information can be downloaded at: https://www.mdpi.com/article/10.3390/foods12132599/s1. Figure S1: GC chromatogram of FAMEs in PSO sample with a miniature of punicic and behenic acids; Figure S2: Chromatogram and linear regression curve of δ-tocopherol, γ-tocopherol and α-tocopherol obtained by UV detector; Figure S3: Chromatogram and linear regression curve of δ-tocopherol, γ-tocopherol and α-tocopherol obtained by fluorescence detector; Figure S4: HPLC-UV chromatogram of PSO sample; Figure S5: HPLC-UV chromatogram of PSO sample; Figure S6: GC-MS chromatogram of unsaponifiable fraction of PSO sample; Figure S7: Mass spectra of squalene; Figure S8: Mass spectra of γ-tocopherol; Figure S9: Mass spectra of campesterol; Figure S10: Mass spectra of stigmasterol; Figure S11: Mass spectra of β-Sitosterol; Figure S12: Mass spectra of lupeol; Figure S13: Calibration curve of caffeic acid ranging from 25 to 150 μg for the determination of total phenolic content by Folin–Ciocalteu spectrophotometric assay using a UV-visible spectrophotometer; Figures S14–S45: GC-FID chromatogram of FAMEs in each PSO sample; Table S1: Composition of fatty acids expressed as range, mean and SD; Table S2: Retention time (min.), coefficient of determination (R^2^) and linear regression model of external standards used for tocopherols calibration, obtained by UV detector (220 nm) and fluorescence detector; Table S3: Composition of unsaponifiable fraction expressed as range, mean and SD.
