# Supplementary material for: Functional Compounds of Cold-Pressed Pomegranate Seed Oil: Fatty Acids and Phytosterols Profile as Quality Biomarkers for Origin Discrimination
Source: Foods. 2023 Jul 5;12(13):2599. doi: 10.3390/foods12132599 (PMC10341364; doi:10.3390/foods12132599)
Supplement: Supplementary file 1 [file foods-12-02599-s001.zip › foods-2426540-supplementary.pdf]

Supplementary material

Functional compounds of cold-pressed pomegranate seed oil: fatty acids and phytosterols profile as quality biomarkers for origin discrimination.

Giuseppe Iriti [1], Sonia Bonacci [1]*, Vincenzo Lopreiato [2], Marialaura Frisina [1], Manuela Oliverio [1] and Antonio Procopio [1].

[1] Dipartimento di Scienze della Salute, Università "Magna Græcia" di Catanzaro, Viale Europa – Campus Universitario "S. Venuta" – Loc. Germaneto - 88100 (CZ), Italy.
[2]Dipartimento di Scienze Veterinarie, Università di Messina, piazza Pugliatti, 1 – 98122, Messina, Italy.

Figure. S1. GC chromatogram of FAMEs in PSO sample **with a miniature of punicic and behenic acids**. pag. 3
Table S1. Composition of fatty acids expressed as range, means and SD pag.
Table S2. Retention time (min.), coefficient of determination (R^2) and linear regression model of external standards used for tocopherols calibration, obtained by UV detector (220nm) (a) and fluorescence detector (b) pag.
Figure S2. Chromatogram (a) and linear regression curve of δ-tocopherol (b), γ-tocopherol (c) and α-tocopherol (d) obtained by UV detector pag. 5
Figure S3. Chromatogram (a) and linear regression curve of δ-tocopherol (b), γ-tocopherol (c) and α-tocopherol (d)obtained by fluorescence detector pag. 6
Figure S4. HPLC-UV chromatogram of PSO sample pag 7
Figure S5. HPLC-UV chromatogram of PSO sample pag. 7
Figure S6. GC-MS chromatogram of unsaponifiable fraction of PSO sample. pag. 8
Figure S7. Mass spectra of squalene pag. 9
Figure S8. Mass spectra of γ-tocopherol pag. 10
Figure S9. Mass spectra of campesterol pag. 11
Figure S10. Mass spectra of stigmasterol pag. 12
Figure S11. Mass spectra of β-Sitosterol pag. 13
Figure S12. Mass spectra of lupeol pag. 14
Figure S13. Calibration curve of caffeic acid ranging from 25 to 150 µg for the determination of total phenolic content by Folin Ciocalteau spectrophotometric assay using a UV-Visible spectrophotometer pag. 15
Table S3. Composition of unsaposifiable fraction expressed as range, means and SD pag. 16
Figure S14. GC-FID chromatogram of FAMEs in PSO-01 sample. pag. 17
Figure S15. GC-FID chromatogram of FAMEs in PSO-02 sample. pag. 18
Figure S16. GC-FID chromatogram of FAMEs in PSO-03 sample. pag. 19
Figure S17. GC-FID chromatogram of FAMEs in PSO-04 sample. pag. 20
Figure S18. GC-FID chromatogram of FAMEs in PSO-05 sample. pag. 21
Figure S19. GC-FID chromatogram of FAMEs in PSO-06 sample. pag. 22
Figure S20. GC-FID chromatogram of FAMEs in PSO-07 sample. pag. 23
Figure S21. GC-FID chromatogram of FAMEs in PSO-08 sample. pag. 24
Figure S22. GC-FID chromatogram of FAMEs in PSO-09 sample. pag. 25
Figure S23. GC-FID chromatogram of FAMEs in PSO-10 sample. pag. 26
Figure S24. GC-FID chromatogram of FAMEs in PSO-11 sample. pag. 27
Figure S25. GC-FID chromatogram of FAMEs in PSO-12 sample. pag. 28
Figure S26. GC-FID chromatogram of FAMEs in PSO-13 sample. pag. 29
Figure S27. GC-FID chromatogram of FAMEs in PSO-14 sample. pag. 30
Figure S28. GC-FID chromatogram of FAMEs in PSO-15 sample. pag. 31
Figure S29. GC-FID chromatogram of FAMEs in PSO-16 sample. pag. 32

Figure S30. GC-FID chromatogram of FAMEs in PSO-17 sample. pag. 33
Figure S31. GC-FID chromatogram of FAMEs in PSO-18 sample. pag. 34
Figure S32. GC-FID chromatogram of FAMEs in PSO-19 sample. pag. 35
Figure S33. GC-FID chromatogram of FAMEs in PSO-20 sample. pag. 36
Figure S34. GC-FID chromatogram of FAMEs in PSO-21 sample. pag. 37
Figure S35. GC-FID chromatogram of FAMEs in PSO-22 sample. pag. 38
Figure S36. GC-FID chromatogram of FAMEs in PSO-23 sample. pag. 39
Figure S37. GC-FID chromatogram of FAMEs in PSO-24 sample. pag. 40
Figure S38. GC-FID chromatogram of FAMEs in PSO-25 sample. pag. 41
Figure S39. GC-FID chromatogram of FAMEs in PSO-26 sample. pag. 42
Figure S40. GC-FID chromatogram of FAMEs in PSO-27 sample. pag. 43
Figure S41. GC-FID chromatogram of FAMEs in PSO-28 sample. pag. 44
Figure S42. GC-FID chromatogram of FAMEs in PSO-29 sample. pag. 45
Figure S43. GC-FID chromatogram of FAMEs in PSO-30 sample. pag. 46
Figure S44. GC-FID chromatogram of FAMEs in PSO-31 sample. pag. 47
Figure S45. GC-FID chromatogram of FAMEs in PSO-32 sample. pag. 48

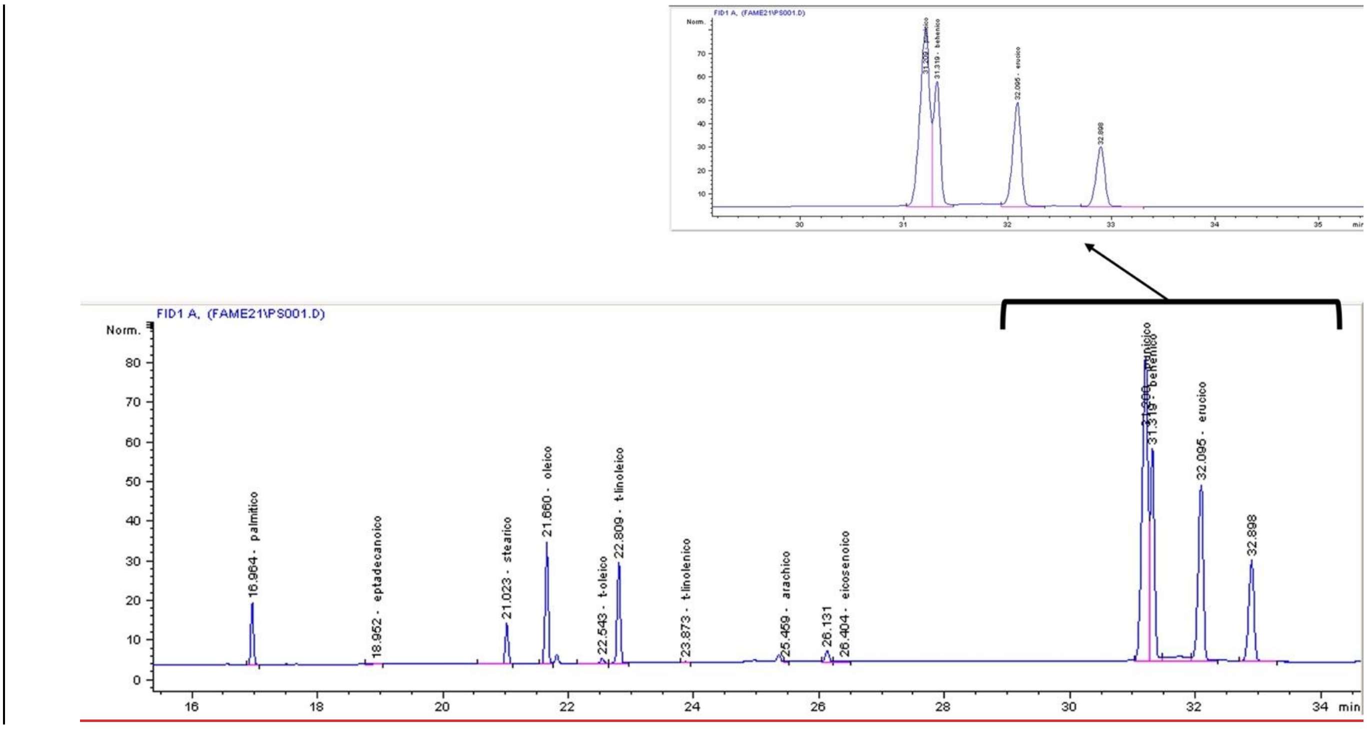

Figure S1. GC chromatogram of FAMEs in PSO sample with a miniature of punicic and behenic acids.

Table S1. Composition of fatty acids reported as relative percentage of peaks area.

FAMEs (%)	Range	Mean*	SD*
Palmitic Acid	2.13-4.42	3.07	0.52
Heptadecanoic Acid	0.02-0.24	0.05	0.07
Stearic Acid	0.65-3.34	2.13	0.47
Oleic Acid	0.56-19.95	5.91	3.88
ElaidicAcid	0.09-5.03	0.39	0.89
Unknown 1	0.00-0.55	0.15	0.19

Linoleic Acid	0.34-30.89	6.81	6.0
Linoelaidic Acid	0.01-1.61	0.13	0.31
Arachidic Acid	0.13-4.19	0.61	0.67
Eicosenoic Acid	0.18-2.94	0.84	0.61
Punicic Acid	16.09-82.81	58.99	18.79
Behenic Acid	0.34-27.71	7.82	7.01
Erucic Acid	1.00-19.4	8.08	3.78
Unknown 2	0.00-21.85	3.78	5.27
Lignoceric Acid	0.04-7.1	0.79	1.35

*Data are expressed as means and standard deviations (SD) of three independent observations.

Table S2. Retention time (min.), coefficient of determination (R^2) and linear regression model of external standards used for tocopherols calibration, obtained by UV detector (220nm) (a) and fluorescence detector (b).

**Tocopherols**	**Rt (min.)**	**R^2**	**Linear regression**
δ-tocopherol [a]	8.5	0.9932	y=0.5883x+0.6729
γ-tocopherol [a]	10.7	0.9900	y=0.8085x+1.6717
α-tocopherol [a]	12.9	0.9905	y=0.8694x+1.6009
δ-tocopherol [b]	8.5	0.9687	y=117304.1685x+6168285.0058
γ-tocopherol [b]	10.7	0.9944	y=172639.3020x+3278101.7020
α-tocopherol [b]	12.9	0.9825	y=98113.3141x-1208544.1429

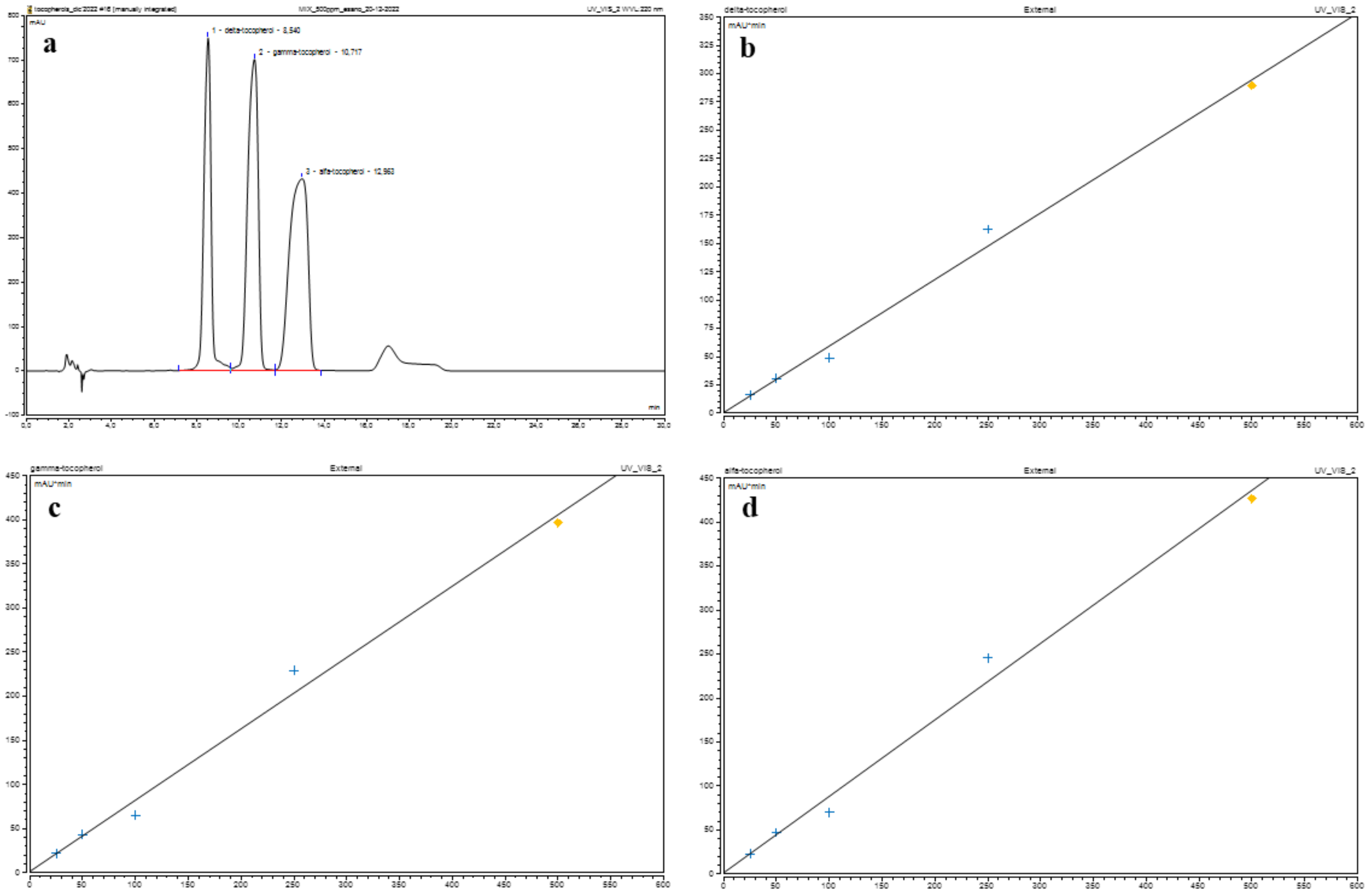

Figure S2. Chromatogram (a) and linear regression curve of δ-tocopherol (b), γ-tocopherol (c) and α-tocopherol (d) obtained by UV detector.

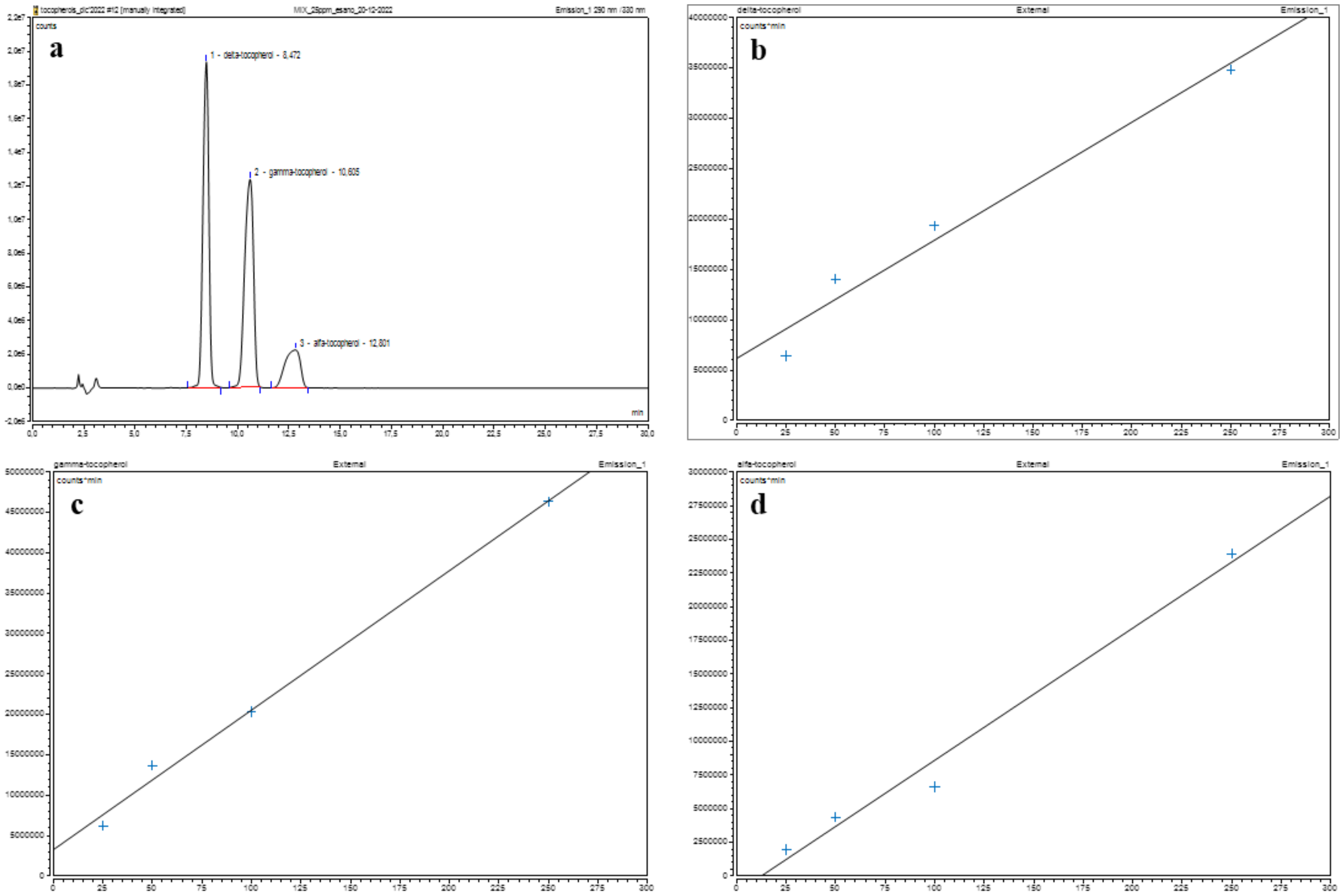

Figure S3. Chromatogram (a) and linear regression curve of δ-tocopherol (b), γ-tocopherol (c) and α-tocopherol (d) obtained by fluorescence detector.

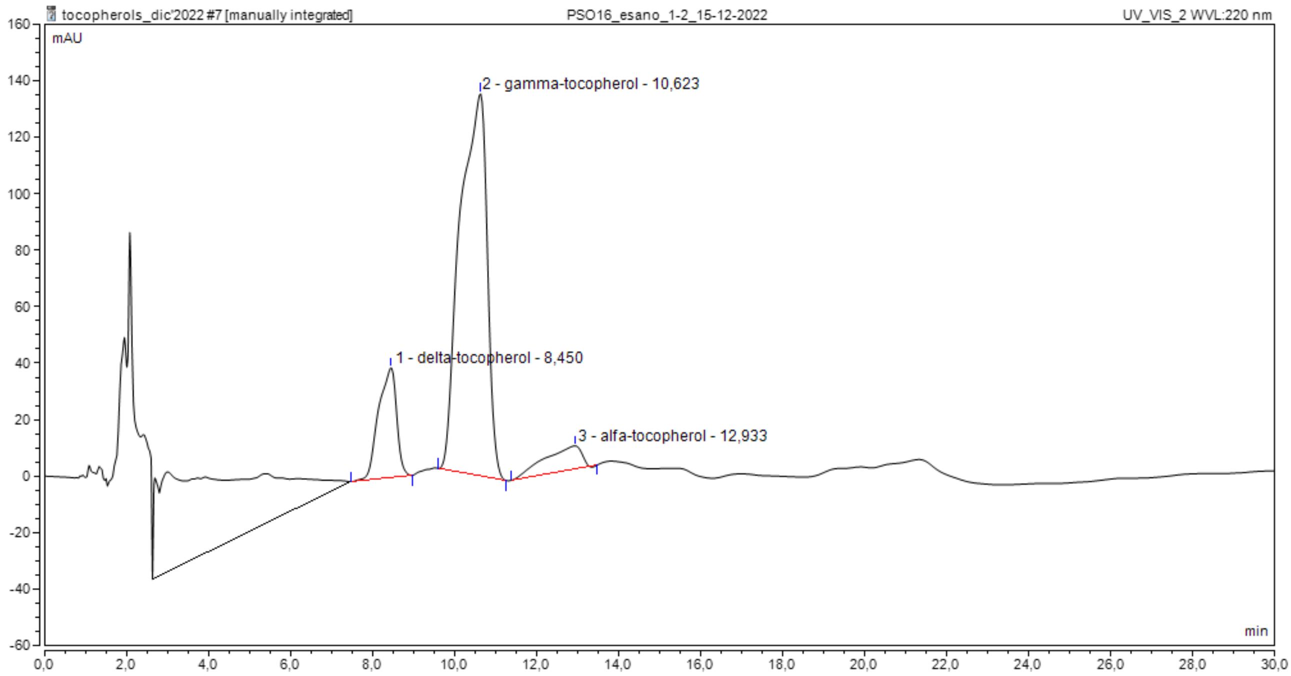

Figure S4. HPLC-UV chromatogram of PSO sample.

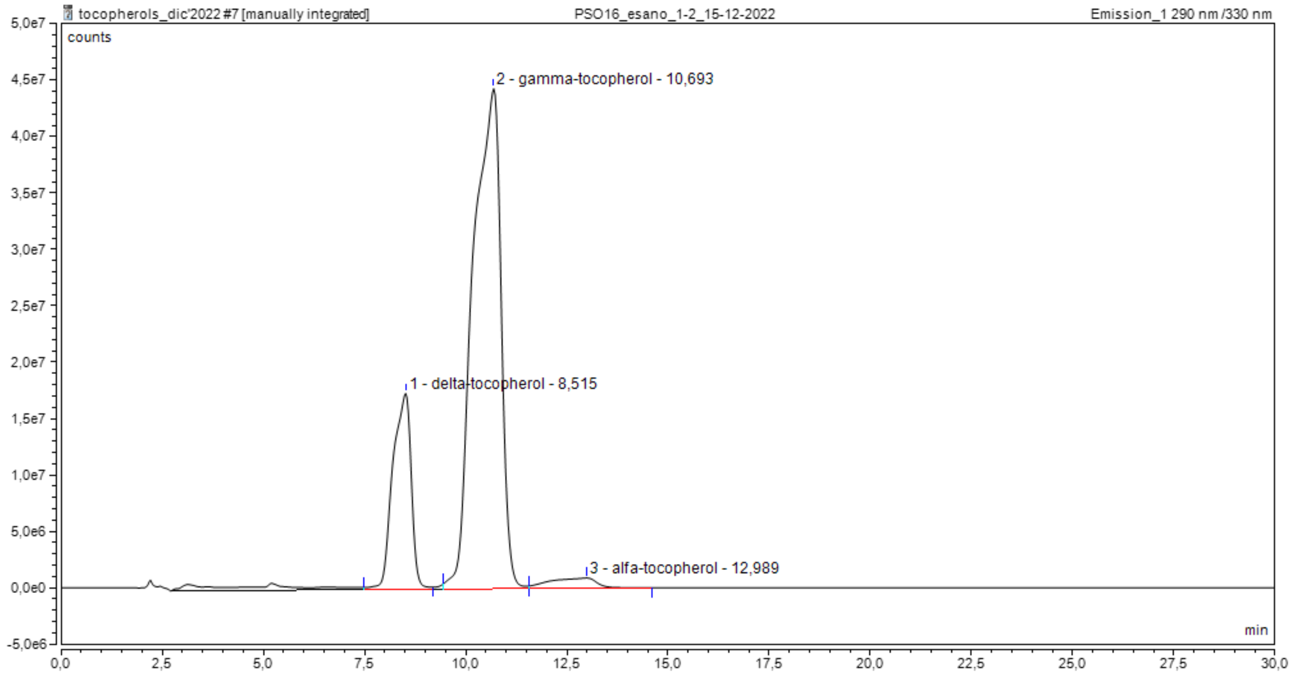

Figure S5. HPLC-UV chromatogram of PSO sample.

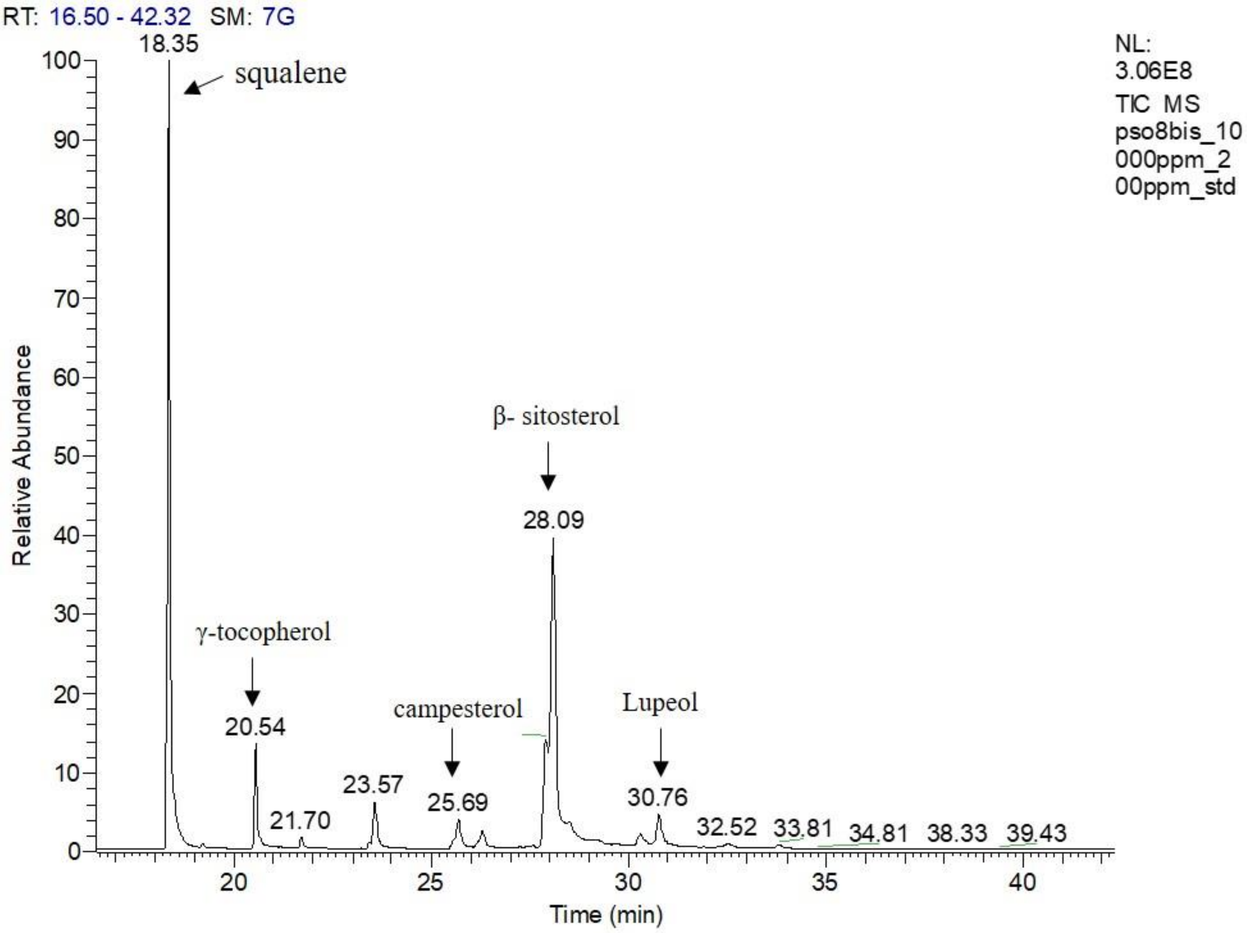

Figure S6. GC-MS chromatogram of unsaponifiable fraction of PSO sample.

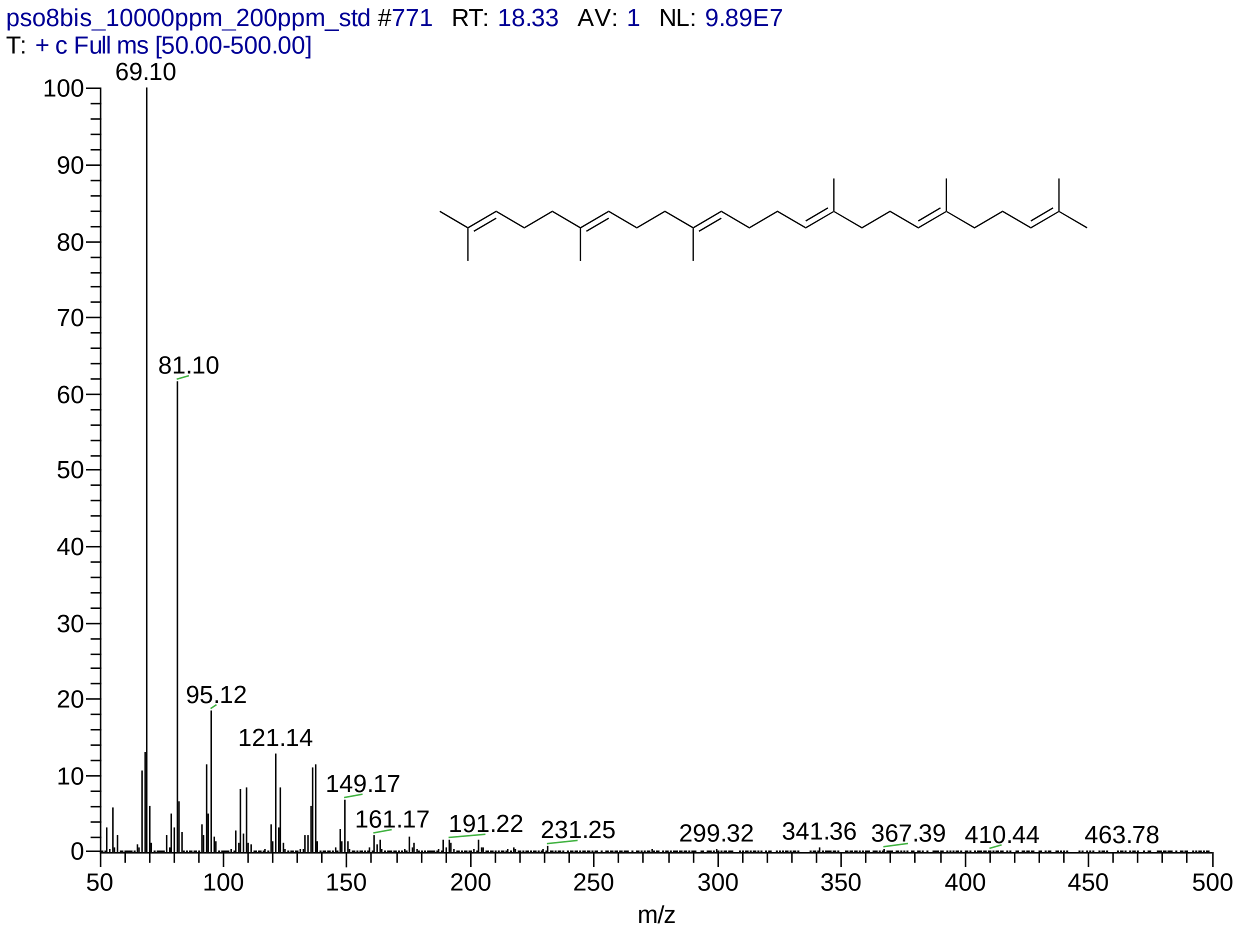

Figure S7. Mass spectra of Squalene (RT 18.33).

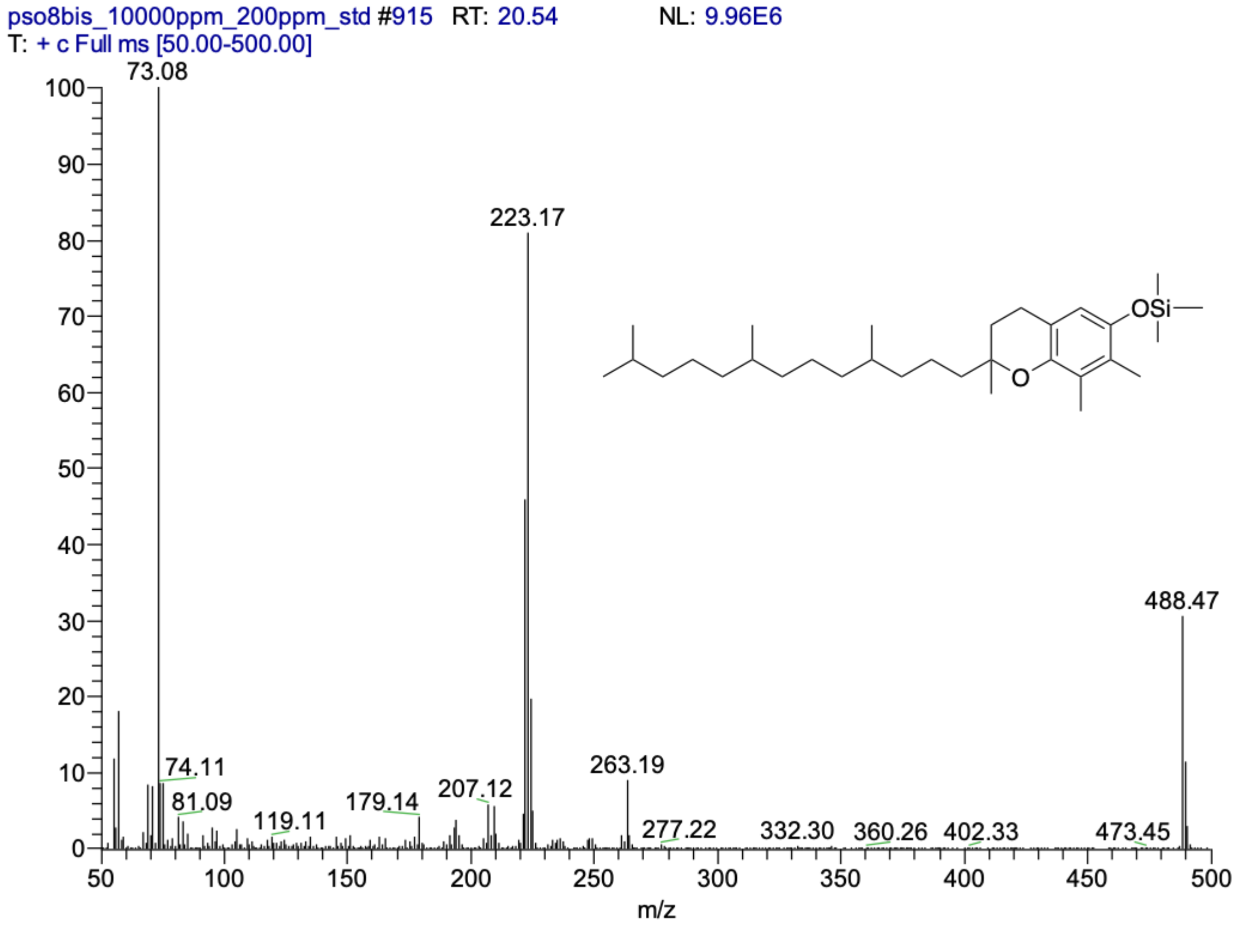

Figure S8. Mass spectra of γ-Tocopherol (RT 20.54).

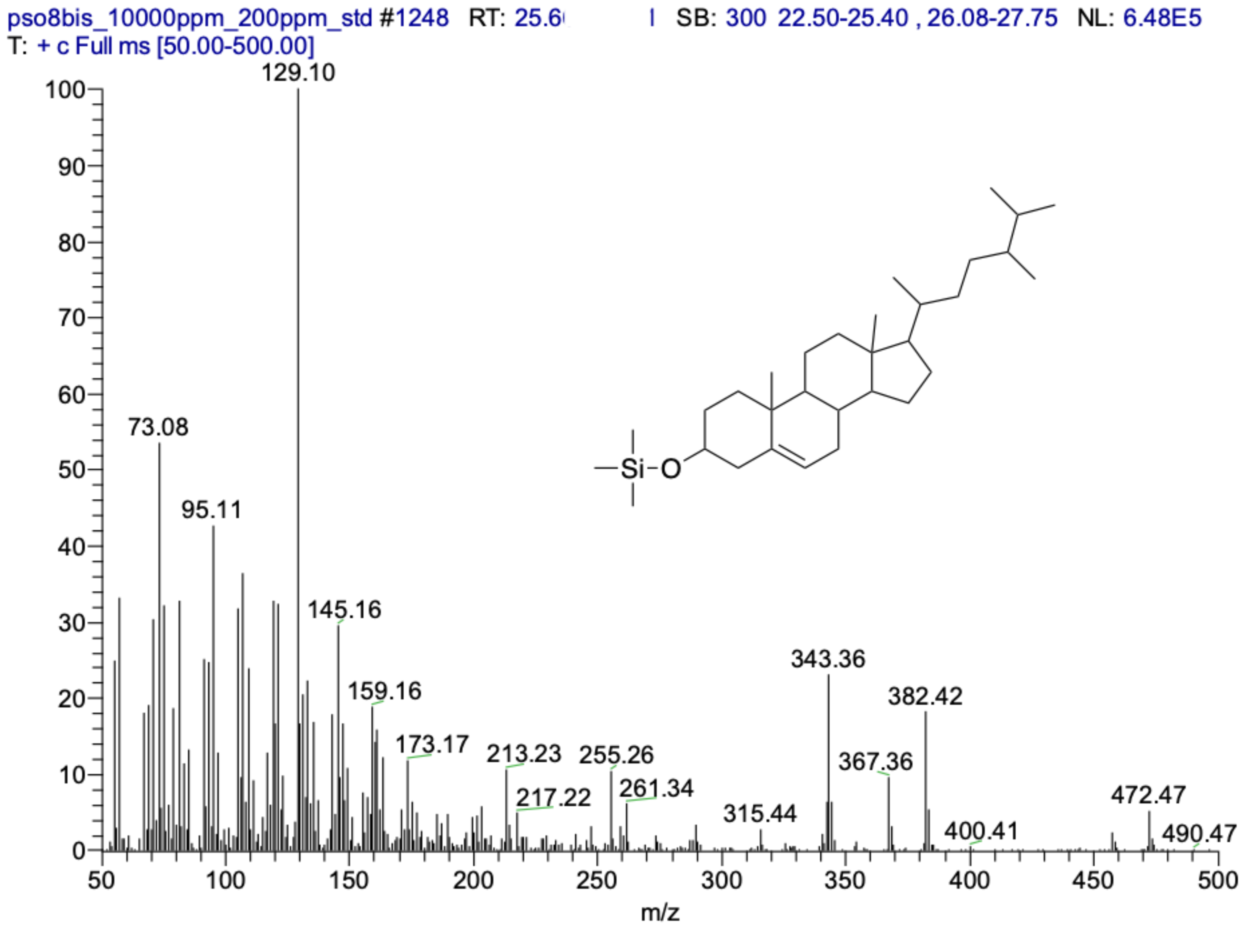

Figure S9. Mass spectra of Campesterol (RT 25.69).

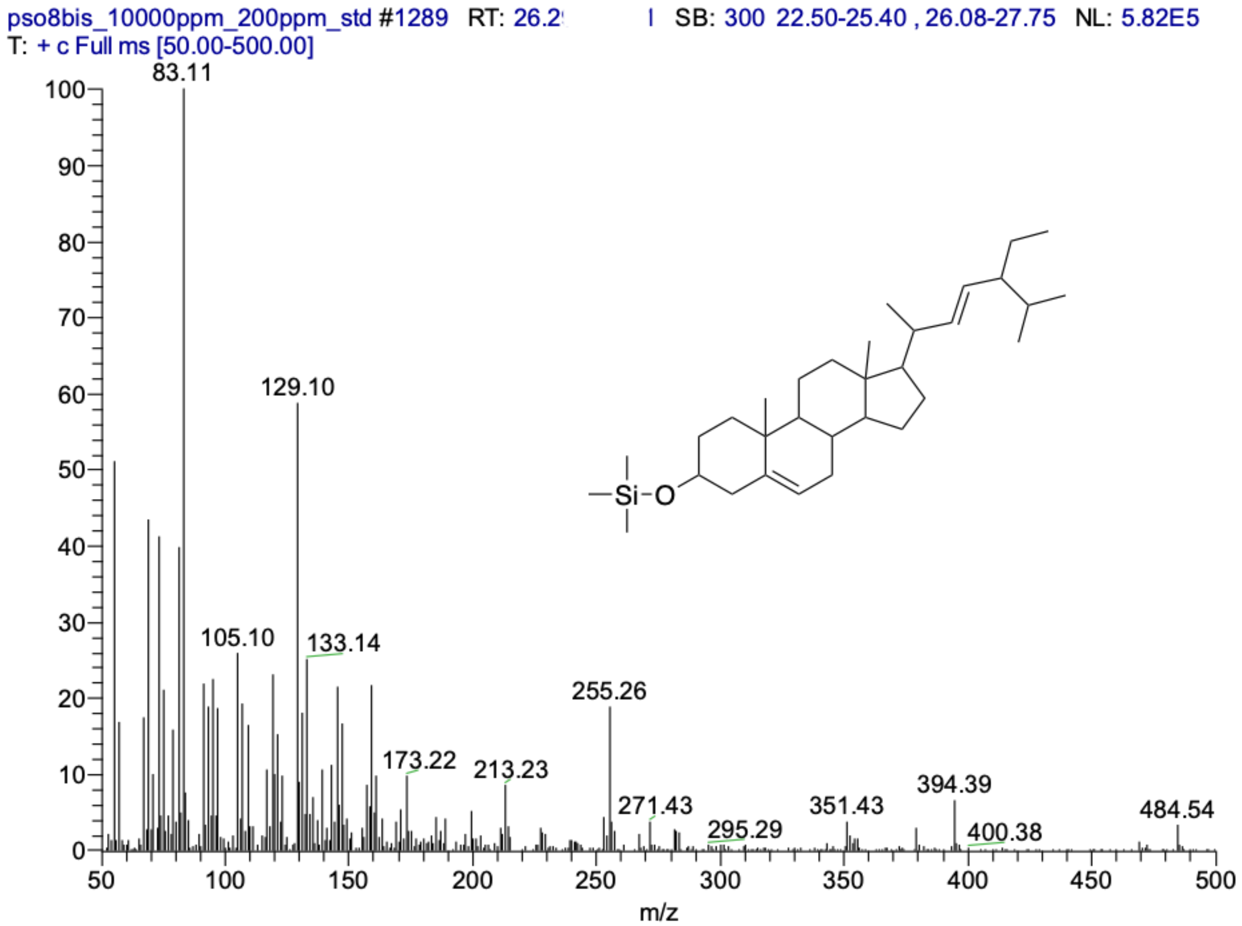

Figure S10. Mass spectra of Stigmasterol (RT 26.29).

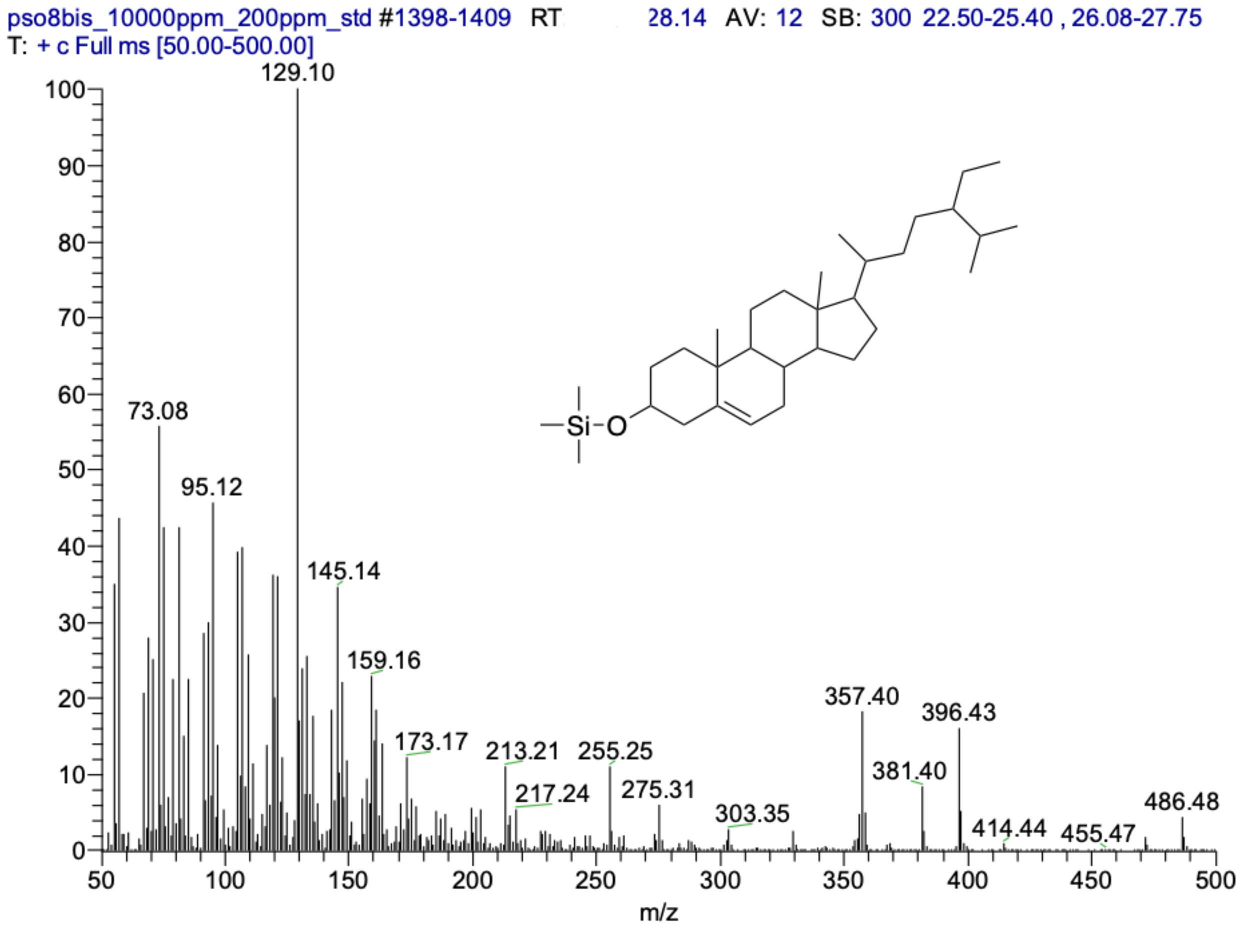

Figure S11. Mass spectra of β-Sitosterol (RT 28.14).

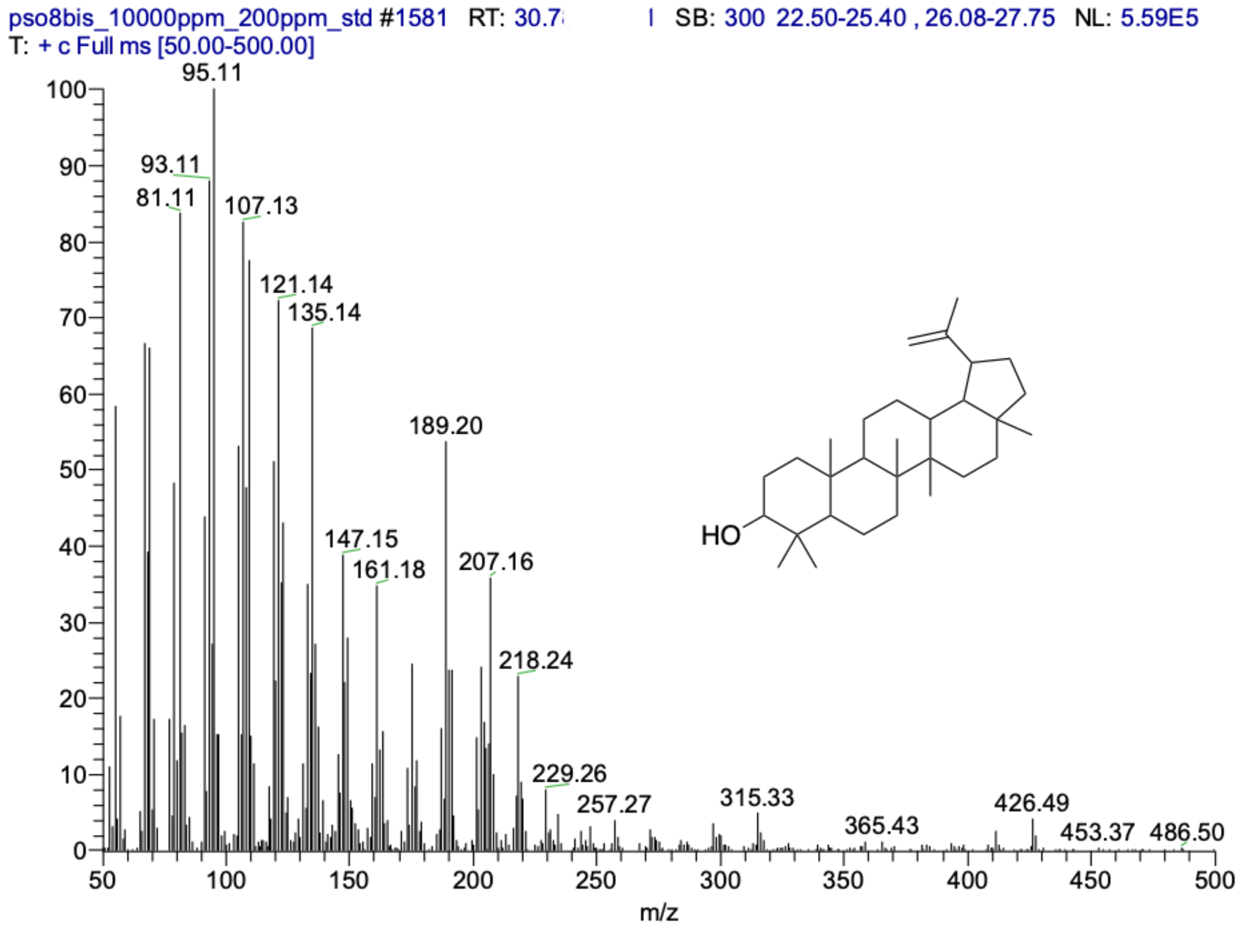

Figure S12. Mass spectra of Lupeol (RT 30.76).

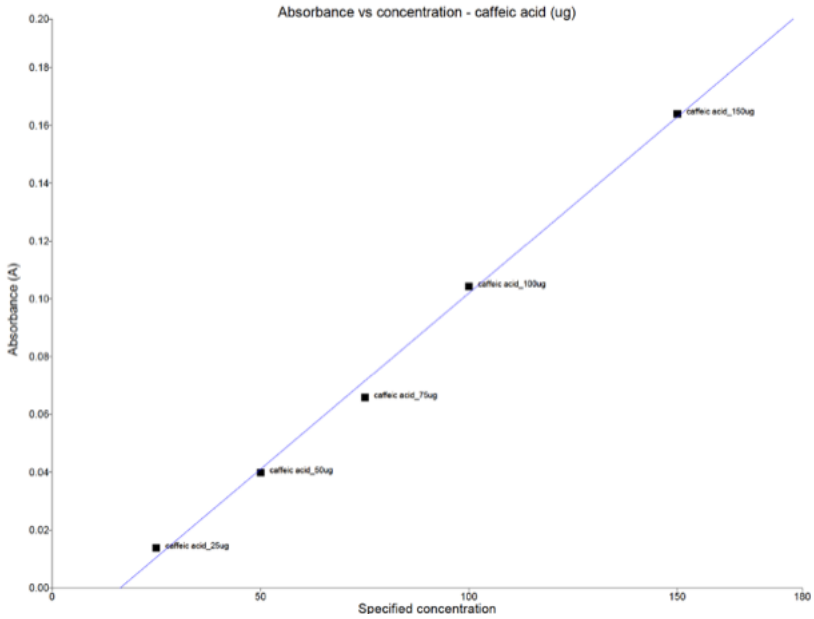

Figure S13. Calibration curve of caffeic acid ranging from 25 to 150 µg for the determination of total phenolic content by Folin Ciocalteau spectrophotometric assay using a UV-visible spectrophotometer.

Table S3. Composition of unsaponifiable fraction reported as mg kg^{-1}.

Unsaponifiable fraction (mg kg^{-1})	Range	Mean*	SD*
Squalene	393.09-3100.19	1318.59	692.06
δ-Tocopherol	5.6-2361.17	353.09	520.75
γ-Tocopherol	5.5-4073.98	1709.21	1091.95
Total tocopherols	11.1-6435.15	2062.30	1563.36
Campesterol	53.72-386.92	215.48	77.42
Stigmasterol	34.43-267.94	144.04	51.97
β-Sitosterol	444.5-3593.56	1839.69	718.10
Lupeol	60.17-570.85	262.80	118.04

*Data expressed as means and standard deviations (SD) of three independent observations.

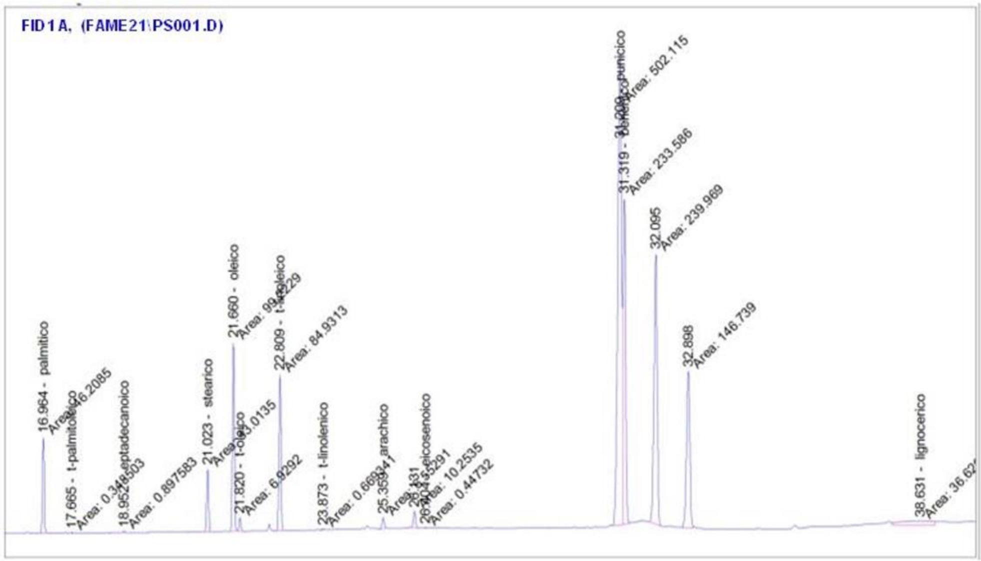

Signal 1: FID1 A,

Peak #	RetTime [min]	Type	Width [min]	Area [pA*s]	Area %	Name
1	13.454		0.0000	0.00000	0.00000	miristico
2	16.964	MM	0.0490	46.20851	3.19008	palmitico
3	17.665	MM	0.0484	3.48503e-1	0.02406	t-palmitoleico
4	17.931		0.0000	0.00000	0.00000	palmitoleico
5	18.952	MM	0.0529	8.97583e-1	0.06197	eptadecanoico
6	19.781		0.0000	0.00000	0.00000	eptadecenoico
7	21.023	MM	0.0535	33.01353	2.27914	stearico
8	21.660	MM	0.0536	99.22294	6.85002	oleico
9	21.820	MM	0.0524	6.92920	0.47837	t-oleico
10	22.183		0.0000	0.00000	0.00000	t-oleico
11	22.809	MM	0.0556	84.93127	5.86337	t-linoleico
12	23.059		0.0000	0.00000	0.00000	linoleico
13	23.873	MM	0.0618	6.69341e-1	0.04621	t-linolenico
14	24.443		0.0000	0.00000	0.00000	linolenico
15	25.359	MM	0.0619	6.55291	0.45239	arachico
16	26.131	MM	0.0634	10.25350	0.70787	?
17	26.404	MM	0.0682	4.47320e-1	0.03088	eicosenoico
18	31.209	MF	0.1092	502.11542	34.66438	punicico
19	31.319	FM	0.0732	233.58627	16.12602	behenico
20	32.095	MM	0.0907	239.96936	16.56669	?
21	32.898	MM	0.0958	146.73863	10.13035	?
22	38.631	MM	0.9263	36.62093	2.52819	lignocerico

Figure S14. GC-FID chromatogram of FAMEs in PSO-01 sample.

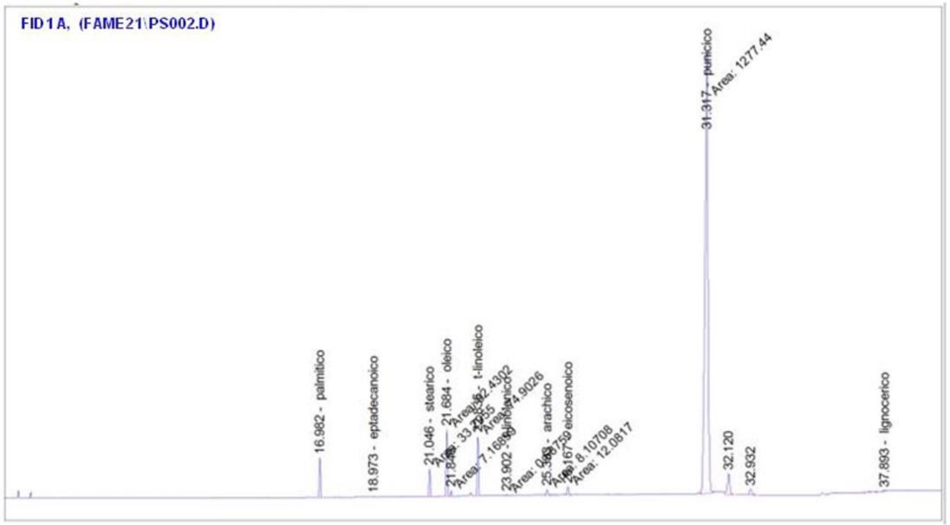

Signal 1: FID1 A,

Peak #	RetTime [min]	Type	Width [min]	Area [pA*s]	Area %	Name
1	13.454		0.0000	0.00000	0.00000	miristico
2	16.982	VB	0.0470	45.18716	2.80900	palmitico
3	17.520		0.0000	0.00000	0.00000	t-palmitoleico
4	17.931		0.0000	0.00000	0.00000	palmitoleico
5	18.973	VP	0.0497	9.82363e-1	0.06107	eptadecanoico
6	19.781		0.0000	0.00000	0.00000	eptadecenoico
7	21.046	MM	0.0523	33.29555	2.06977	stearico
8	21.500		0.0000	0.00000	0.00000	t-oleico
9	21.684	MM	0.0537	82.43022	5.12416	oleico
10	21.846	MM	0.0535	7.16899	0.44565	?
11	22.183		0.0000	0.00000	0.00000	t-oleico
12	22.835	MM	0.0549	74.90265	4.65622	t-linoleico
13	23.059		0.0000	0.00000	0.00000	linoleico
14	23.902	MM	0.0568	6.87590e-1	0.04274	t-linolenico
15	24.443		0.0000	0.00000	0.00000	linolenico
16	25.393	MM	0.0646	8.10708	0.50396	arachico
17	26.167	MM	0.0659	12.08170	0.75104	eicosenoico
18	31.226		0.0000	0.00000	0.00000	behenico
19	31.317	MM	0.1259	1277.44458	79.41052	punicico
20	32.120	VB	0.0923	48.26136	3.00010	?
21	32.932	BP	0.0948	13.69370	0.85125	?
22	37.893	VV	0.1929	4.41626	0.27453	lignocerico

Figure S15. GC-FID chromatogram of FAMEs in PSO-02 sample.

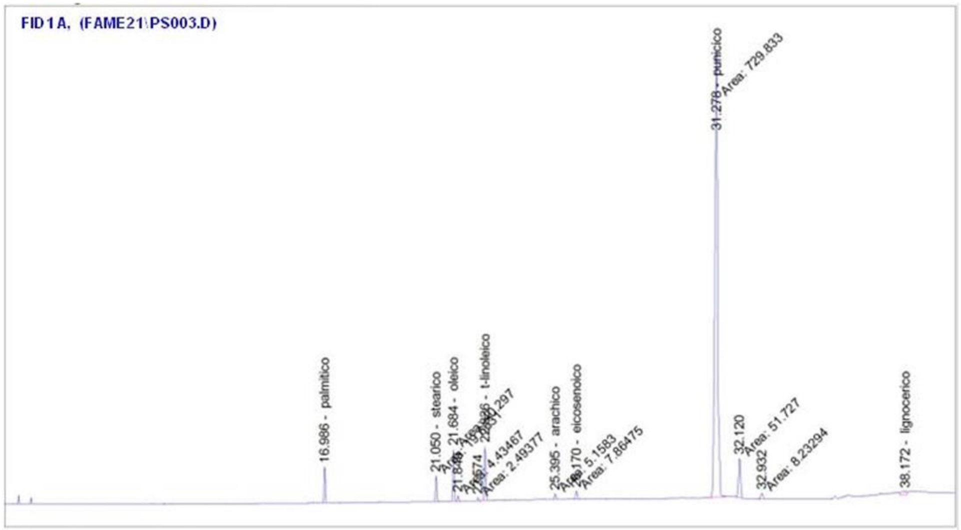

Signal 1: FID1 A,

Peak #	RetTime [min]	Type	Width [min]	Area [pA*s]	Area %	Name
1	13.454		0.0000	0.00000	0.00000	miristico
2	16.986	PB	0.0473	25.96648	2.73499	palmitico
3	17.520		0.0000	0.00000	0.00000	t-palmitoleico
4	17.931		0.0000	0.00000	0.00000	palmitoleico
5	19.107		0.0000	0.00000	0.00000	eptadecanoico
6	19.781		0.0000	0.00000	0.00000	eptadecenoico
7	21.050	MM	0.0528	19.66308	2.07107	stearico
8	21.500		0.0000	0.00000	0.00000	t-oleico
9	21.684	MM	0.0541	40.29704	4.24440	oleico
10	21.849	MM	0.0559	4.43467	0.46709	?
11	22.183		0.0000	0.00000	0.00000	t-oleico
12	22.574	MM	0.0563	2.49377	0.26266	?
13	22.836	BB	0.0505	42.20690	4.44556	t-linoleico
14	23.059		0.0000	0.00000	0.00000	linoleico
15	24.060		0.0000	0.00000	0.00000	t-linolenico
16	24.443		0.0000	0.00000	0.00000	linolenico
17	25.395	MM	0.0645	5.15830	0.54331	arachico
18	26.170	MM	0.0673	7.86475	0.82838	eicosenoico
19	31.226		0.0000	0.00000	0.00000	behenico
20	31.278	MM	0.1138	729.83264	76.87162	punicico
21	32.120	MM	0.0911	51.72702	5.44829	?
22	32.932	MM	0.0996	8.23294	0.86716	?
23	38.172	VV	0.1996	11.53990	1.21547	lignocerico

Figure S16. GC-FID chromatogram of FAMEs in PSO-03 sample.

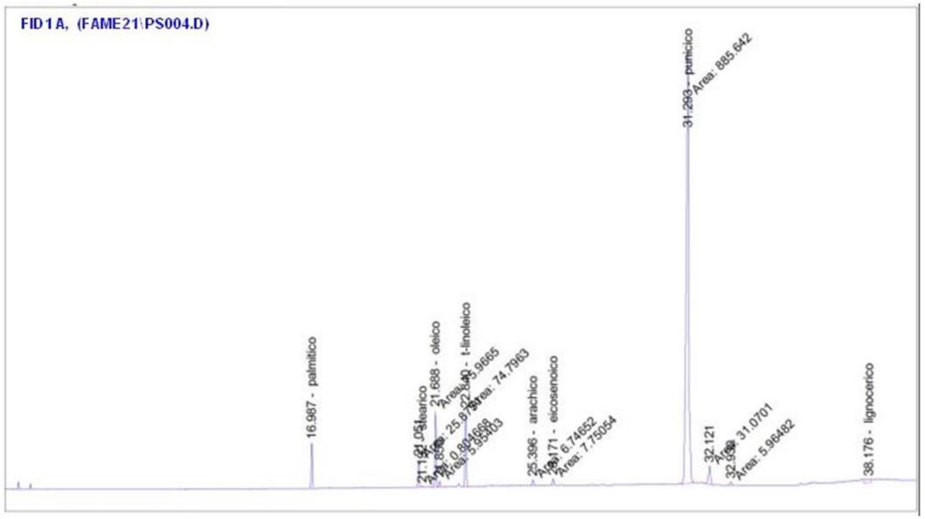

Signal 1: FID1 A,

Peak #	RetTime [min]	Type	Width [min]	Area [pA*s]	Area %	Name
1	13.454		0.0000	0.00000	0.00000	miristico
2	16.987	VB	0.0445	40.97486	3.48047	palmitico
3	17.520		0.0000	0.00000	0.00000	t-palmitoleico
4	17.931		0.0000	0.00000	0.00000	palmitoleico
5	19.107		0.0000	0.00000	0.00000	eptadecanoico
6	19.781		0.0000	0.00000	0.00000	eptadecenoico
7	21.051	MM	0.0525	25.87514	2.19788	?
8	21.192	MM	0.0545	8.04668e-1	0.06835	stearico
9	21.500		0.0000	0.00000	0.00000	t-oleico
10	21.688	MM	0.0547	75.96647	6.45271	oleico
11	21.850	MM	0.0541	5.95403	0.50574	?
12	22.183		0.0000	0.00000	0.00000	t-oleico
13	22.840	MM	0.0552	74.79631	6.35332	t-linoleico
14	23.059		0.0000	0.00000	0.00000	linoleico
15	24.060		0.0000	0.00000	0.00000	t-linolenico
16	24.443		0.0000	0.00000	0.00000	linolenico
17	25.396	MM	0.0661	6.74652	0.57306	arachico
18	26.171	MM	0.0651	7.75054	0.65834	eicosenoico
19	31.226		0.0000	0.00000	0.00000	behenico
20	31.293	MM	0.1130	885.64154	75.22781	punicico
21	32.121	MM	0.0937	31.07008	2.63914	?
22	32.930	MM	0.1046	5.96482	0.50666	?
23	38.176	VV	0.2075	15.73449	1.33651	lignocerico

Figure S17. GC-FID chromatogram of FAMEs in PSO-04 sample.

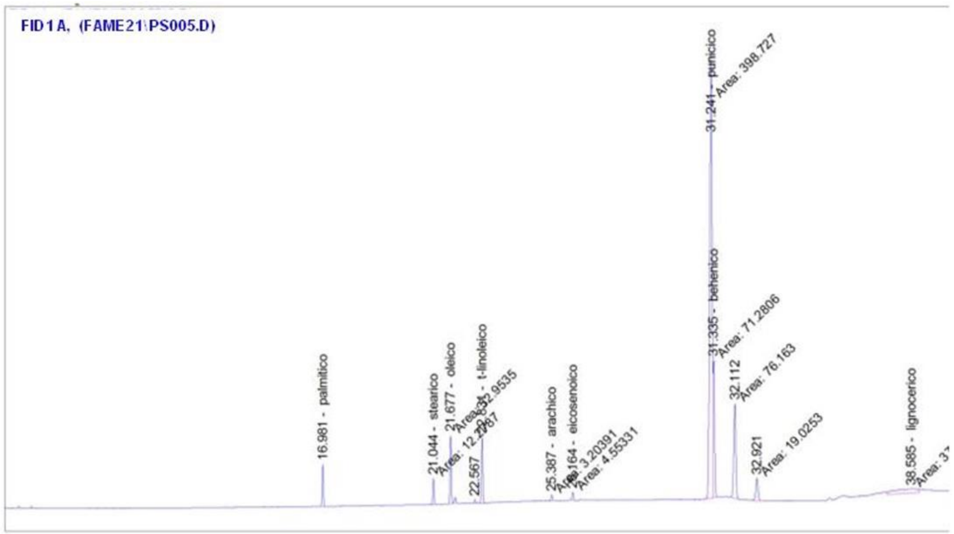

Signal 1: FID1 A,

Peak #	RetTime [min]	Type	Width [min]	Area [pA*s]	Area %	Name
1	13.454		0.0000	0.00000	0.00000	miristico
2	16.981	PV	0.0469	18.34941	2.59015	palmitico
3	17.520		0.0000	0.00000	0.00000	t-palmitoleico
4	17.931		0.0000	0.00000	0.00000	palmitoleico
5	19.107		0.0000	0.00000	0.00000	eptadecanoico
6	19.781		0.0000	0.00000	0.00000	eptadecenoico
7	21.044	MM	0.0526	12.27873	1.73323	stearico
8	21.500		0.0000	0.00000	0.00000	t-oleico
9	21.677	MM	0.0547	32.95350	4.65162	oleico
10	22.183		0.0000	0.00000	0.00000	t-oleico
11	22.567	BV	0.0602	2.06351	0.29128	?
12	22.831	VB	0.0512	31.97880	4.51403	t-linoleico
13	23.059		0.0000	0.00000	0.00000	linoleico
14	24.060		0.0000	0.00000	0.00000	t-linolenico
15	24.443		0.0000	0.00000	0.00000	linolenico
16	25.387	MM	0.0623	3.20391	0.45225	arachico
17	26.164	MM	0.0641	4.55331	0.64273	eicosenoico
18	31.241	MF	0.1014	398.72745	56.28321	punicico
19	31.335	FM	0.0590	71.28056	10.06176	behenico
20	32.112	MM	0.0917	76.16296	10.75094	?
21	32.921	MM	0.0965	19.02531	2.68556	?
22	38.585	MM	1.0498	37.85308	5.34323	lignocerico

Figure S18. GC-FID chromatogram of FAMEs in PSO-05 sample.

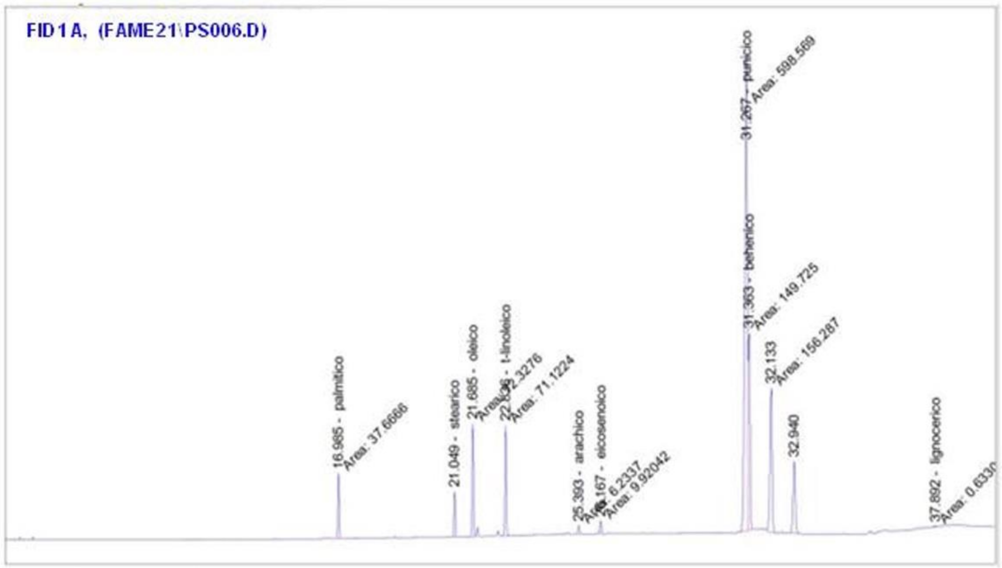

Signal 1: FID1 A,

Peak #	RetTime [min]	Type	Width [min]	Area [pA*s]	Area %	Name
1	13.454		0.0000	0.00000	0.00000	miristico
2	16.985	MM	0.0491	37.66658	3.10945	palmitico
3	17.520		0.0000	0.00000	0.00000	t-palmitoleico
4	17.931		0.0000	0.00000	0.00000	palmitoleico
5	19.107		0.0000	0.00000	0.00000	eptadecanoico
6	19.781		0.0000	0.00000	0.00000	eptadecenoico
7	21.049	PB	0.0498	28.35135	2.34046	stearico
8	21.500		0.0000	0.00000	0.00000	t-oleico
9	21.685	MM	0.0549	72.32762	5.97078	oleico
10	22.183		0.0000	0.00000	0.00000	t-oleico
11	22.836	MM	0.0550	71.12236	5.87128	t-linoleico
12	23.059		0.0000	0.00000	0.00000	linoleico
13	24.060		0.0000	0.00000	0.00000	t-linolenico
14	24.443		0.0000	0.00000	0.00000	linolenico
15	25.393	MM	0.0625	6.23370	0.51460	arachico
16	26.167	MM	0.0658	9.92042	0.81895	eicosenoico
17	31.267	MF	0.1081	598.56866	49.41296	punicico
18	31.363	FM	0.0651	149.72479	12.36006	behenico
19	32.133	MM	0.0932	156.28687	12.90177	?
20	32.940	BV	0.0896	80.52434	6.64743	?
21	37.892	MM	0.0734	6.33003e-1	0.05226	lignocerico

Figure S19. GC-FID chromatogram of FAMEs in PSO-06 sample.

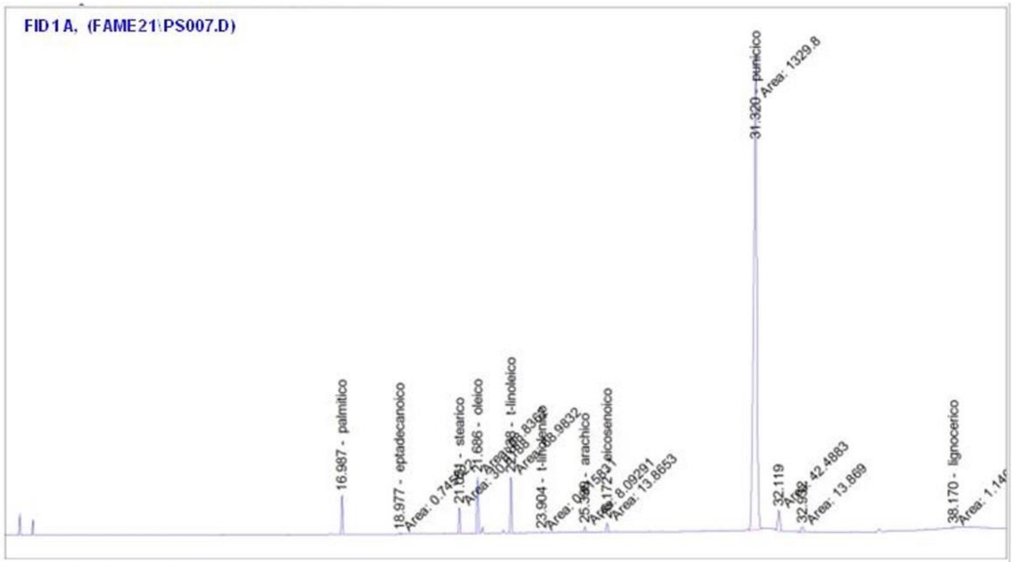

Signal 1: FID1 A,

Peak #	RetTime [min]	Type	Width [min]	Area [pA*s]	Area %	Name
1	13.454		0.0000	0.00000	0.00000	miristico
2	16.987	VV	0.0446	43.48398	2.67948	palmitico
3	17.520		0.0000	0.00000	0.00000	t-palmitoleico
4	17.931		0.0000	0.00000	0.00000	palmitoleico
5	18.977	MM	0.0492	7.45622e-1	0.04595	eptadecanoico
6	19.781		0.0000	0.00000	0.00000	eptadecenoico
7	21.051	MM	0.0531	30.91878	1.90521	stearico
8	21.500		0.0000	0.00000	0.00000	t-oleico
9	21.686	MM	0.0545	68.83620	4.24169	oleico
10	22.183		0.0000	0.00000	0.00000	t-oleico
11	22.838	MM	0.0550	68.98318	4.25074	t-linoleico
12	23.059		0.0000	0.00000	0.00000	linoleico
13	23.904	MM	0.0527	6.15831e-1	0.03795	t-linolenico
14	24.443		0.0000	0.00000	0.00000	linolenico
15	25.399	MM	0.0651	8.09291	0.49868	arachico
16	26.172	MM	0.0673	13.86532	0.85438	eicosenoico
17	31.226		0.0000	0.00000	0.00000	behenico
18	31.320	MM	0.1260	1329.80469	81.94254	punicico
19	32.119	MM	0.0930	42.48827	2.61813	?
20	32.932	MM	0.1186	13.86901	0.85461	?
21	38.170	MM	0.1191	1.14652	0.07065	lignocerico

Figure S20. GC-FID chromatogram of FAMEs in PSO-07 sample.

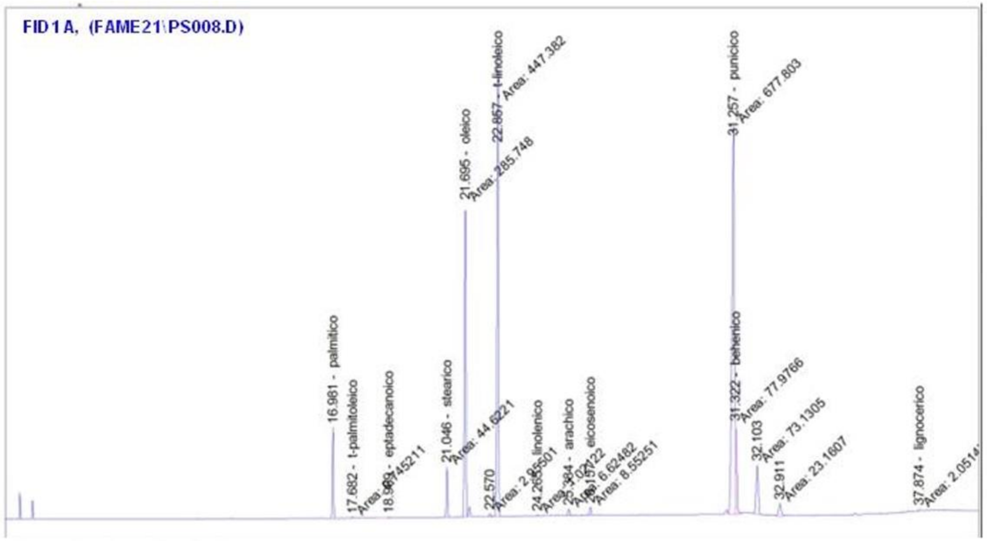

Signal 1: FID1 A,

Peak #	RetTime [min]	Type	Width [min]	Area [pA*s]	Area %	Name
1	13.454		0.0000	0.00000	0.00000	miristico
2	16.981	BB	0.0474	74.52077	4.31443	palmitico
3	17.682	MM	0.0485	7.45211e-1	0.04314	t-palmitoleico
4	17.931		0.0000	0.00000	0.00000	palmitoleico
5	18.969	VP	0.0501	9.53397e-1	0.05520	eptadecanoico
6	19.781		0.0000	0.00000	0.00000	eptadecenoico
7	21.046	MM	0.0535	44.62213	2.58342	stearico
8	21.500		0.0000	0.00000	0.00000	t-oleico
9	21.695	MM	0.0556	285.74780	16.54354	oleico
10	22.183		0.0000	0.00000	0.00000	t-oleico
11	22.570	MM	0.0576	2.95501	0.17108	?
12	22.857	MM	0.0587	447.38214	25.90146	t-linoleico
13	23.059		0.0000	0.00000	0.00000	linoleico
14	24.060		0.0000	0.00000	0.00000	t-linolenico
15	24.265	MM	0.0590	1.02122	0.05912	linolenico
16	25.384	MM	0.0627	6.62482	0.38355	arachico
17	26.157	MM	0.0641	8.55251	0.49515	eicosenoico
18	31.257	MF	0.1063	677.80267	39.24179	punicico
19	31.322	FM	0.0530	77.97662	4.51450	behenico
20	32.103	MM	0.0904	73.13046	4.23393	?
21	32.911	MM	0.1113	23.16067	1.34090	?
22	37.874	MM	0.0755	2.05147	0.11877	lignocerico

Figure S21. GC-FID chromatogram of FAMEs in PSO-08 sample.

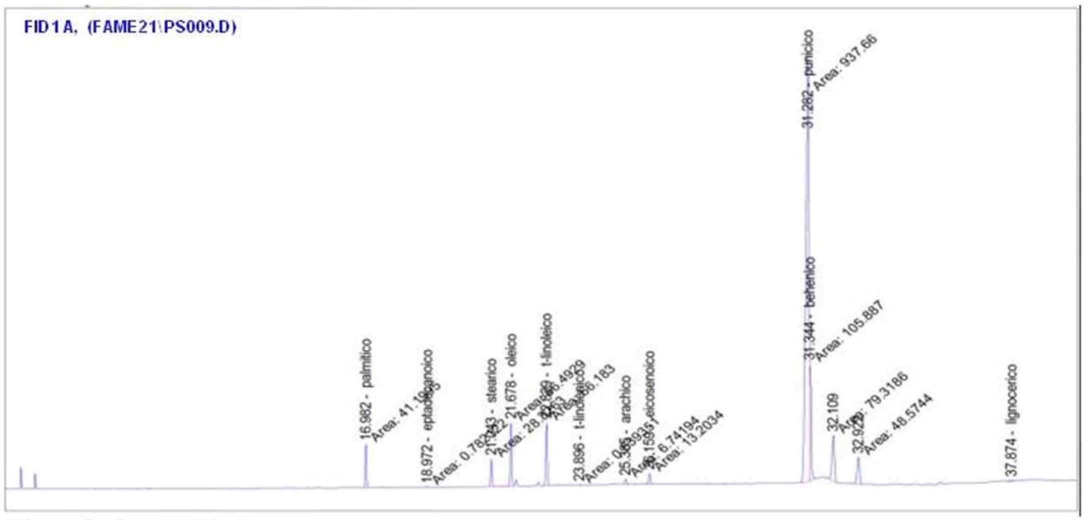

Signal 1: FID1 A,

Peak #	RetTime [min]	Type	Width [min]	Area [pA*s]	Area %	Name
1	13.454		0.0000	0.00000	0.00000	miristico
2	16.982	MM	0.0490	41.19951	2.94168	palmitico
3	17.520		0.0000	0.00000	0.00000	t-palmitoleico
4	17.931		0.0000	0.00000	0.00000	palmitoleico
5	18.972	MM	0.0515	7.82122e-1	0.05584	eptadecanoico
6	19.781		0.0000	0.00000	0.00000	eptadecenoico
7	21.043	MM	0.0531	28.32627	2.02252	stearico
8	21.500		0.0000	0.00000	0.00000	t-oleico
9	21.678	MF	0.0546	66.49287	4.74765	oleico
10	22.183		0.0000	0.00000	0.00000	t-oleico
11	22.829	MM	0.0548	66.18300	4.72553	t-linoleico
12	23.059		0.0000	0.00000	0.00000	linoleico
13	23.896	MM	0.0591	6.39351e-1	0.04565	t-linolenico
14	24.443		0.0000	0.00000	0.00000	linolenico
15	25.385	MM	0.0656	6.74194	0.48138	arachico
16	26.159	MM	0.0652	13.20340	0.94274	eicosenoico
17	31.282	MF	0.1145	937.66046	66.94984	punicico
18	31.344	FM	0.0474	105.88686	7.56042	behenico
19	32.109	MM	0.0904	79.31858	5.66342	?
20	32.922	MM	0.0948	48.57444	3.46826	?
21	37.874	VV	0.1410	5.53284	0.39505	lignocerico

Figure S22. GC-FID chromatogram of FAMEs in PSO-09 sample.

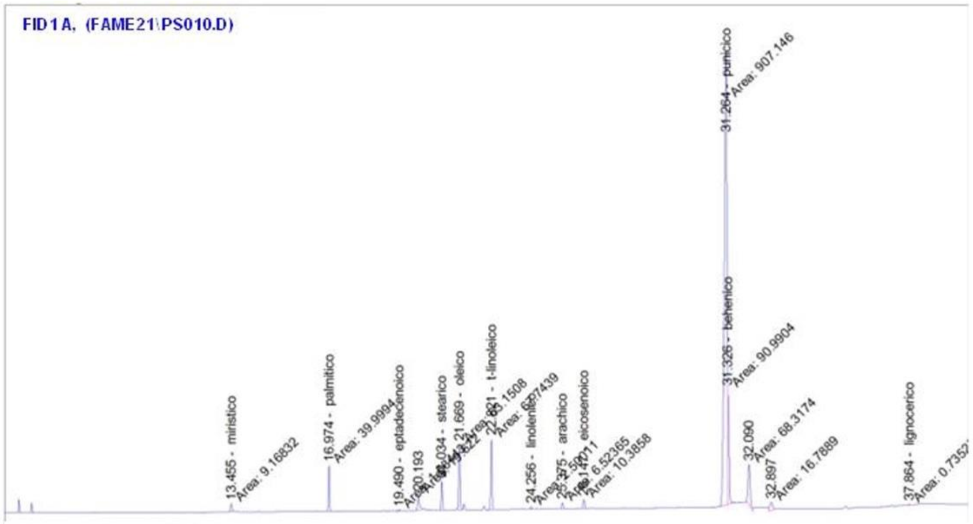

Signal 1: FID1 A,

Peak #	RetTime [min]	Type	Width [min]	Area [pA*s]	Area %	Name
1	13.455	MM	0.0643	9.16832	0.68844	miristico
2	16.974	MM	0.0490	39.99938	3.00352	palmitico
3	17.520		0.0000	0.00000	0.00000	t-palmitoleico
4	17.931		0.0000	0.00000	0.00000	palmitoleico
5	19.107		0.0000	0.00000	0.00000	eptadecanoico
6	19.490	MM	0.0852	1.58443	0.11897	eptadecenoico
7	20.193	MM	0.0732	19.62197	1.47340	?
8	21.034	PV	0.0497	27.09271	2.03437	stearico
9	21.500		0.0000	0.00000	0.00000	t-oleico
10	21.669	FM	0.0548	63.15081	4.74194	oleico
11	22.183		0.0000	0.00000	0.00000	t-oleico
12	22.821	MM	0.0546	67.74392	5.08684	t-linoleico
13	23.059		0.0000	0.00000	0.00000	linoleico
14	24.060		0.0000	0.00000	0.00000	t-linolenico
15	24.256	MM	0.0649	2.50011	0.18773	linolenico
16	25.375	MM	0.0635	6.52365	0.48986	arachico
17	26.147	MM	0.0655	10.38582	0.77986	eicosenoico
18	31.264	MF	0.1138	907.14606	68.11688	punicico
19	31.326	FM	0.0451	90.99041	6.83240	behenico
20	32.090	MM	0.0945	68.31741	5.12990	?
21	32.897	MM	0.1204	16.78894	1.26067	?
22	37.864	MM	0.0771	7.35270e-1	0.05521	lignocerico

Figure S23. GC-FID chromatogram of FAMEs in PSO-10 sample.

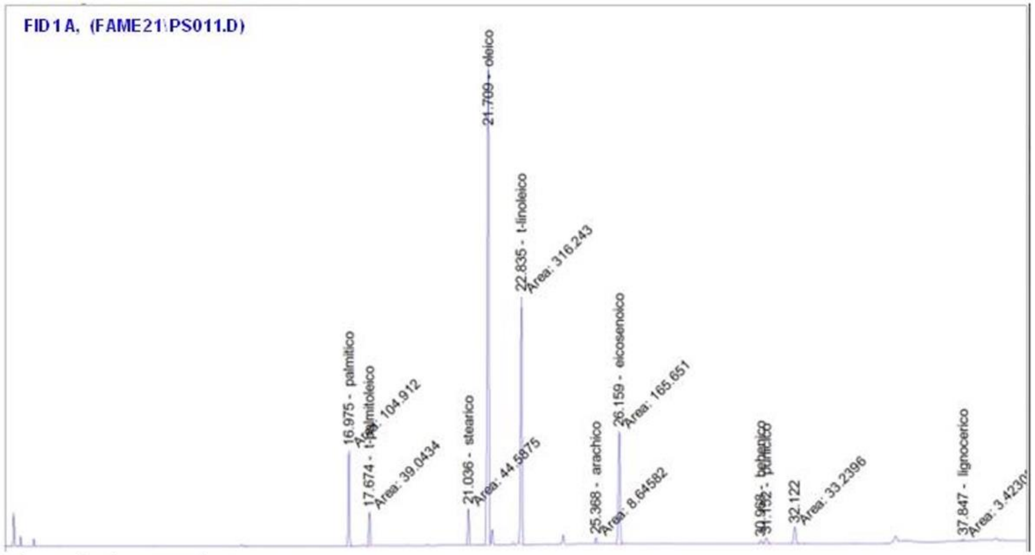

Signal 1: FID1 A,

Peak #	RetTime [min]	Type	Width [min]	Area [pA*s]	Area %	Name
1	13.454		0.0000	0.00000	0.00000	miristico
2	16.975	MM	0.0495	104.91170	7.34178	palmitico
3	17.674	MM	0.0510	39.04345	2.73228	t-palmitoleico
4	17.931		0.0000	0.00000	0.00000	palmitoleico
5	19.107		0.0000	0.00000	0.00000	eptadecanoico
6	19.781		0.0000	0.00000	0.00000	eptadecenoico
7	21.036	MM	0.0546	44.58746	3.12025	stearico
8	21.500		0.0000	0.00000	0.00000	t-oleico
9	21.709	VV	0.0600	693.92670	48.56135	oleico
10	22.183		0.0000	0.00000	0.00000	t-oleico
11	22.835	MM	0.0577	316.24344	22.13088	t-linoleico
12	23.059		0.0000	0.00000	0.00000	linoleico
13	24.060		0.0000	0.00000	0.00000	t-linolenico
14	24.443		0.0000	0.00000	0.00000	linolenico
15	25.368	MM	0.0620	8.64582	0.60504	arachico
16	26.159	MM	0.0664	165.65128	11.59236	eicosenoico
17	30.968	BV	0.0810	6.41822	0.44915	behenico
18	31.152	VB	0.0888	12.87826	0.90123	punicico
19	32.122	MM	0.0903	33.23963	2.32613	?
20	37.847	MM	0.0893	3.42306	0.23955	lignocerico

Figure S24. GC-FID chromatogram of FAMEs in PSO-11 sample.

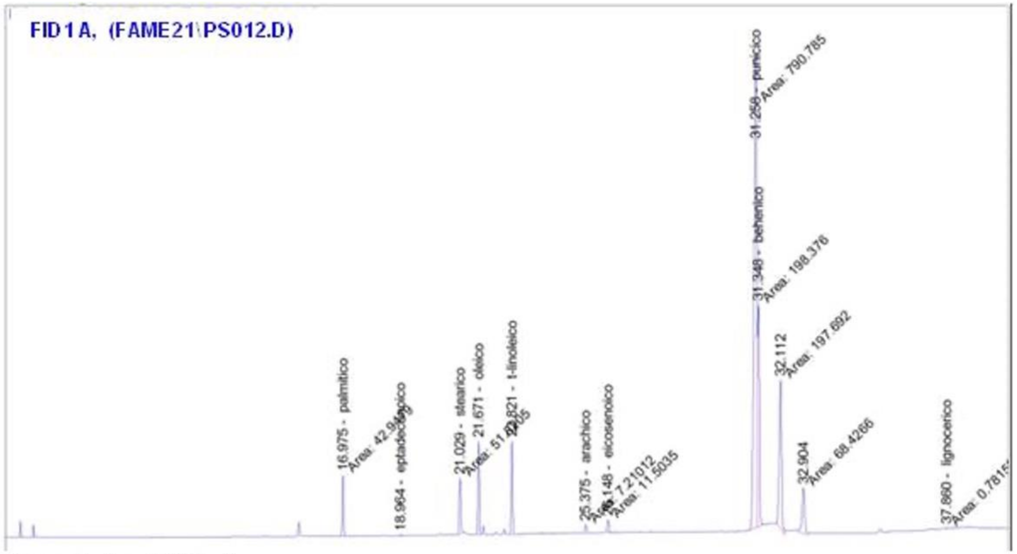

Signal 1: FID1 A,

Peak #	RetTime [min]	Type	Width [min]	Area [pA*s]	Area %	Name
1	13.454		0.0000	0.00000	0.00000	miristico
2	16.975	MM	0.0490	42.94794	2.83792	palmitico
3	17.520		0.0000	0.00000	0.00000	t-palmitoleico
4	17.931		0.0000	0.00000	0.00000	palmitoleico
5	18.964	PV	0.0514	8.55024e-1	0.05650	eptadecanoico
6	19.781		0.0000	0.00000	0.00000	eptadecenoico
7	21.029	MM	0.0657	51.82048	3.42420	stearico
8	21.500		0.0000	0.00000	0.00000	t-oleico
9	21.671	VV	0.0502	71.27010	4.70939	oleico
10	22.183		0.0000	0.00000	0.00000	t-oleico
11	22.821	BP	0.0503	71.69385	4.73739	t-linoleico
12	23.059		0.0000	0.00000	0.00000	linoleico
13	24.060		0.0000	0.00000	0.00000	t-linolenico
14	24.443		0.0000	0.00000	0.00000	linolenico
15	25.375	MM	0.0633	7.21012	0.47643	arachico
16	26.148	MM	0.0638	11.50354	0.76013	eicosenoico
17	31.258	MF	0.1176	790.78522	52.25353	punicico
18	31.348	FM	0.0632	198.37614	13.10831	behenico
19	32.112	MM	0.0938	197.69160	13.06307	?
20	32.904	MM	0.1066	68.42661	4.52150	?
21	37.860	MM	0.0743	7.81527e-1	0.05164	lignocerico

Figure S25. GC-FID chromatogram of FAMEs in PSO-12 sample.

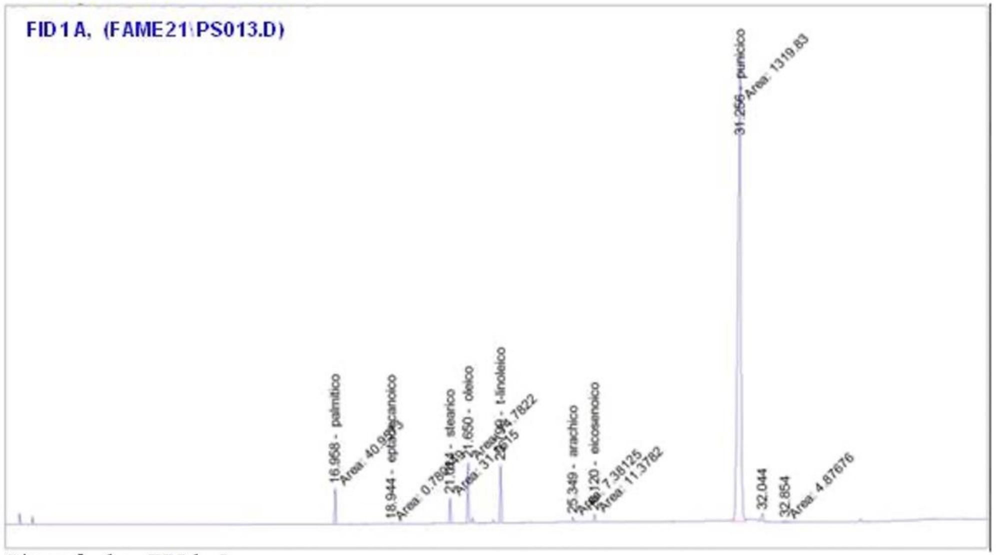

Signal 1: FID1 A,

Peak #	RetTime [min]	Type	Width [min]	Area [pA*s]	Area %	Name
1	13.454		0.0000	0.00000	0.00000	miristico
2	16.958	MM	0.0491	40.95733	2.59109	palmitico
3	17.520		0.0000	0.00000	0.00000	t-palmitoleico
4	17.931		0.0000	0.00000	0.00000	palmitoleico
5	18.944	MM	0.0496	7.80849e-1	0.04940	eptadecanoico
6	19.781		0.0000	0.00000	0.00000	eptadecenoico
7	21.014	MM	0.0524	31.16150	1.97137	stearico
8	21.500		0.0000	0.00000	0.00000	t-oleico
9	21.650	MM	0.0534	74.78225	4.73096	oleico
10	22.183		0.0000	0.00000	0.00000	t-oleico
11	22.799	VB	0.0514	74.11263	4.68860	t-linoleico
12	23.059		0.0000	0.00000	0.00000	linoleico
13	24.060		0.0000	0.00000	0.00000	t-linolenico
14	24.443		0.0000	0.00000	0.00000	linolenico
15	25.349	MM	0.0631	7.38125	0.46696	arachico
16	26.120	MM	0.0637	11.37820	0.71982	eicosenoico
17	31.256	MM	0.1229	1319.82825	83.49647	punicico
18	31.334		0.0000	0.00000	0.00000	behenico
19	32.044	BP	0.0844	15.44037	0.97681	?
20	32.854	MM	0.1259	4.87676	0.30852	?
21	38.096		0.0000	0.00000	0.00000	lignocerico

Figure S26. GC-FID chromatogram of FAMEs in PSO-13 sample.

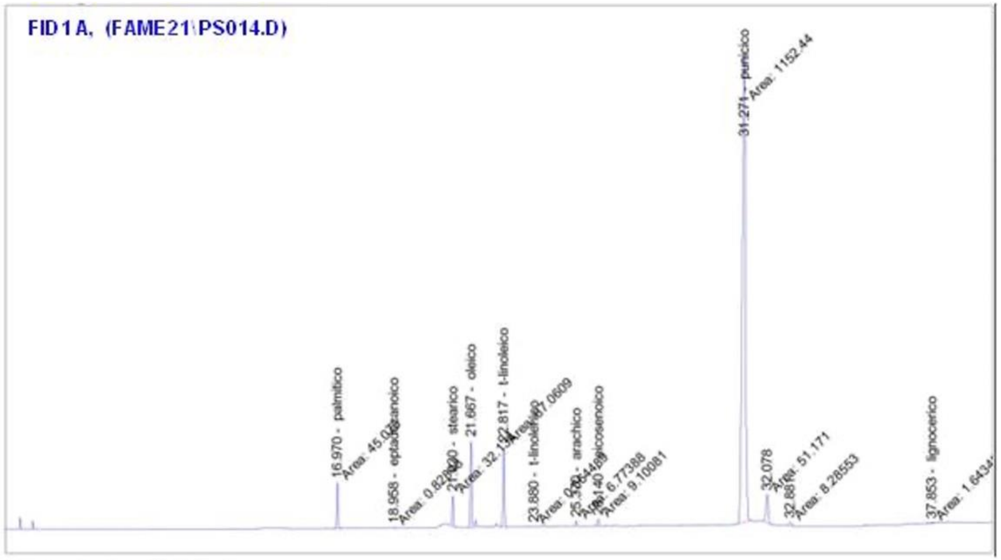

Signal 1: FID1 A,

Peak #	RetTime [min]	Type	Width [min]	Area [pA*s]	Area %	Name
1	13.454		0.0000	0.00000	0.00000	miristico
2	16.970	MM	0.0492	45.07296	3.02970	palmitico
3	17.520		0.0000	0.00000	0.00000	t-palmitoleico
4	17.931		0.0000	0.00000	0.00000	palmitoleico
5	18.958	MM	0.0497	8.28480e-1	0.05569	eptadecanoico
6	19.781		0.0000	0.00000	0.00000	eptadecenoico
7	21.030	MM	0.0527	32.13400	2.15997	stearico
8	21.500		0.0000	0.00000	0.00000	t-oleico
9	21.667	VV	0.0504	92.62521	6.22605	oleico
10	22.183		0.0000	0.00000	0.00000	t-oleico
11	22.817	MM	0.0548	87.06090	5.85203	t-linoleico
12	23.059		0.0000	0.00000	0.00000	linoleico
13	23.880	MM	0.0579	5.64489e-1	0.03794	t-linolenico
14	24.443		0.0000	0.00000	0.00000	linolenico
15	25.370	MM	0.0644	6.77388	0.45532	arachico
16	26.140	MM	0.0651	9.10081	0.61174	eicosenoico
17	31.271	MM	0.1249	1152.44238	77.46454	punicico
18	31.334		0.0000	0.00000	0.00000	behenico
19	32.078	MM	0.0911	51.17101	3.43960	?
20	32.881	MM	0.1064	8.28553	0.55693	?
21	37.853	MM	0.1076	1.64342	0.11047	lignocerico

Figure S27. GC-FID chromatogram of FAMEs in PSO-14 sample.

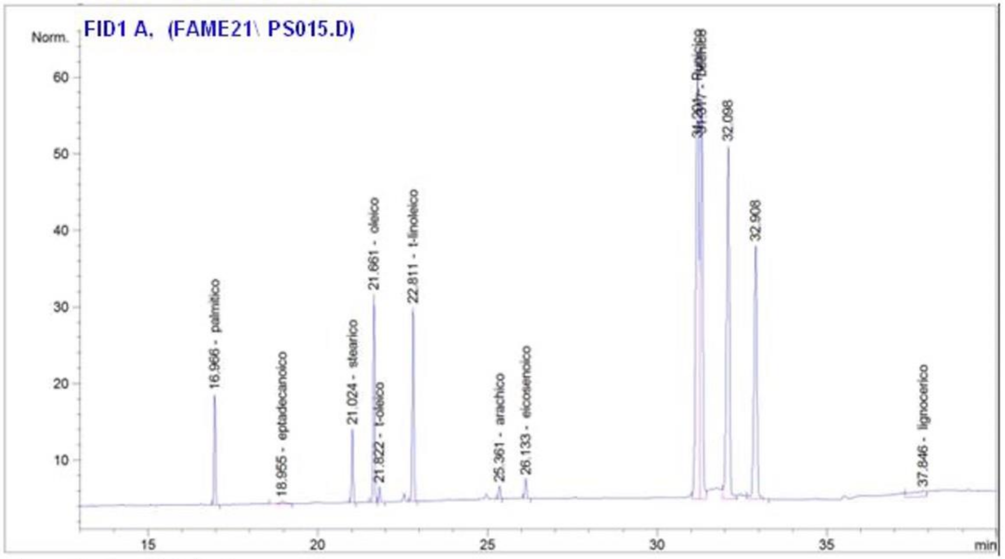

Signal 1: FID1 A,

Peak #	RetTime [min]	Type	Width [min]	Area [pA*s]	Area %	Name
1	13.454		0.0000	0.00000	0.00000	miristico
2	16.966	VB	0.0445	42.90868	3.08902	palmitico
3	17.520		0.0000	0.00000	0.00000	t-palmitoleico
4	17.931		0.0000	0.00000	0.00000	palmitoleico
5	18.955	VV	0.1136	3.20228	0.23053	eptadecanoico
6	19.781		0.0000	0.00000	0.00000	eptadecenoico
7	21.024	BV	0.0524	31.08861	2.23808	stearico
8	21.661	VV	0.0507	88.85754	6.39690	oleico
9	21.822	VB	0.0522	6.73577	0.48491	t-oleico
10	22.183		0.0000	0.00000	0.00000	t-oleico
11	22.811	BP	0.0517	84.95146	6.11570	t-linoleico
12	23.059		0.0000	0.00000	0.00000	linoleico
13	24.060		0.0000	0.00000	0.00000	t-linolenico
14	24.443		0.0000	0.00000	0.00000	linolenico
15	25.361	VV	0.0591	6.39131	0.46011	arachico
16	26.133	VB	0.0620	10.34838	0.74498	eicosenoico
17	31.201	VV	0.0923	362.69617	26.11068	Punicico
18	31.317	VV	0.0764	264.68753	19.05499	beenico
19	32.098	VV	0.0848	266.34842	19.17456	?
20	32.908	VB	0.0898	198.36162	14.28015	?
21	37.846	VV	0.3545	22.49436	1.61938	lignocerico

Figure S28. GC-FID chromatogram of FAMEs in PSO-15 sample.

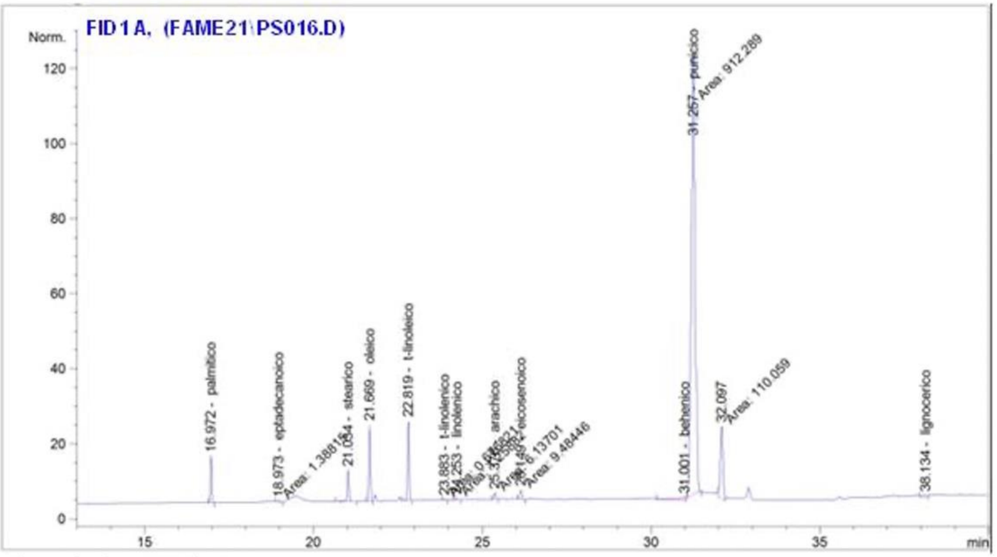

Signal 1: FID1 A,

Peak #	RetTime [min]	Type	Width [min]	Area [pA*s]	Area %	Name
1	13.454		0.0000	0.00000	0.00000	miristico
2	16.972	BB	0.0465	36.32817	2.84449	palmitico
3	17.520		0.0000	0.00000	0.00000	t-palmitoleico
4	17.931		0.0000	0.00000	0.00000	palmitoleico
5	18.973	MM	0.0776	1.38815	0.10869	eptadecanoico
6	19.781		0.0000	0.00000	0.00000	eptadecenoico
7	21.034	VB	0.0551	30.39026	2.37955	stearico
8	21.500		0.0000	0.00000	0.00000	t-oleico
9	21.669	VV	0.0532	66.96437	5.24329	oleico
10	22.183		0.0000	0.00000	0.00000	t-oleico
11	22.819	VB	0.0537	70.51120	5.52101	t-linoleico
12	23.059		0.0000	0.00000	0.00000	linoleico
13	23.883	MM	0.0659	6.76821e-1	0.05299	t-linolenico
14	24.253	MM	0.0611	1.25882	0.09857	linolenico
15	25.378	MM	0.0613	6.13701	0.48053	arachico
16	26.149	MM	0.0654	9.48446	0.74263	eicosenoico
17	31.001	VV	0.3877	24.97685	1.95568	behenico
18	31.257	MM	0.1284	912.28931	71.43199	punicico
19	32.097	MM	0.0948	110.05910	8.61760	?
20	38.134	VV	0.1592	6.67934	0.52299	lignocerico

Figure S29. GC-FID chromatogram of FAMEs in PSO-16 sample.

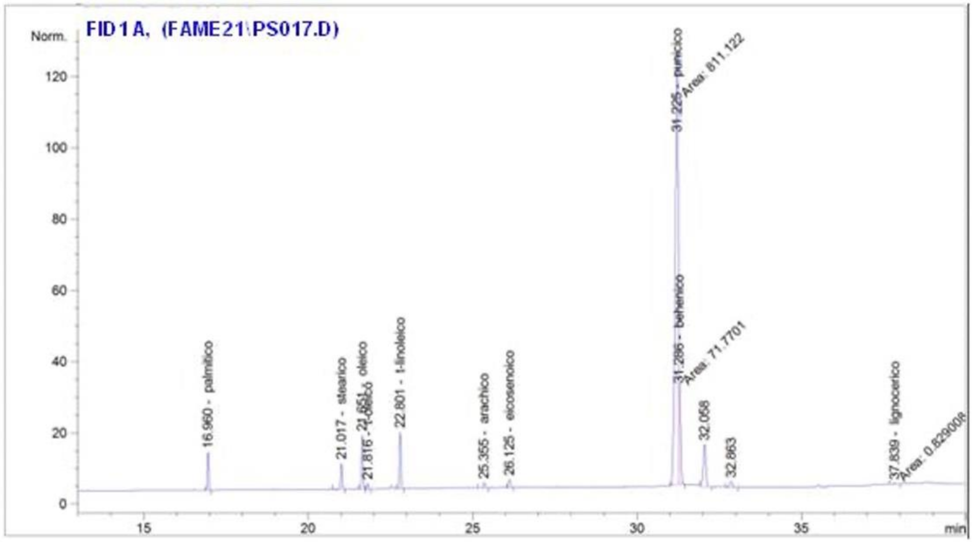

Signal 1: FID1 A,

Peak #	RetTime [min]	Type	Width [min]	Area [pA*s]	Area %	Name
1	13.454		0.0000	0.00000	0.00000	miristico
2	16.960	VB	0.0440	31.35129	2.75237	palmitico
3	17.520		0.0000	0.00000	0.00000	t-palmitoleico
4	17.931		0.0000	0.00000	0.00000	palmitoleico
5	19.107		0.0000	0.00000	0.00000	eptadecanoico
6	19.781		0.0000	0.00000	0.00000	eptadecenoico
7	21.017	BV	0.0502	23.42564	2.05657	stearico
8	21.651	VV	0.0508	49.72750	4.36564	oleico
9	21.816	VP	0.0509	5.17202	0.45406	t-oleico
10	22.183		0.0000	0.00000	0.00000	t-oleico
11	22.801	PB	0.0536	52.59961	4.61779	t-linoleico
12	23.059		0.0000	0.00000	0.00000	linoleico
13	24.060		0.0000	0.00000	0.00000	t-linolenico
14	24.443		0.0000	0.00000	0.00000	linolenico
15	25.355	BV	0.0615	5.85374	0.51391	arachico
16	26.125	VV	0.0627	9.35360	0.82117	eicosenoico
17	31.225	MF	0.1108	811.12158	71.20946	punicico
18	31.286	FM	0.0439	71.77013	6.30080	behenico
19	32.058	VP	0.0894	68.72954	6.03386	?
20	32.863	BP	0.0894	9.13068	0.80159	?
21	37.839	MM	0.1033	8.29008e-1	0.07278	lignocerico

Figure S30. GC-FID chromatogram of FAMEs in PSO-17 sample.

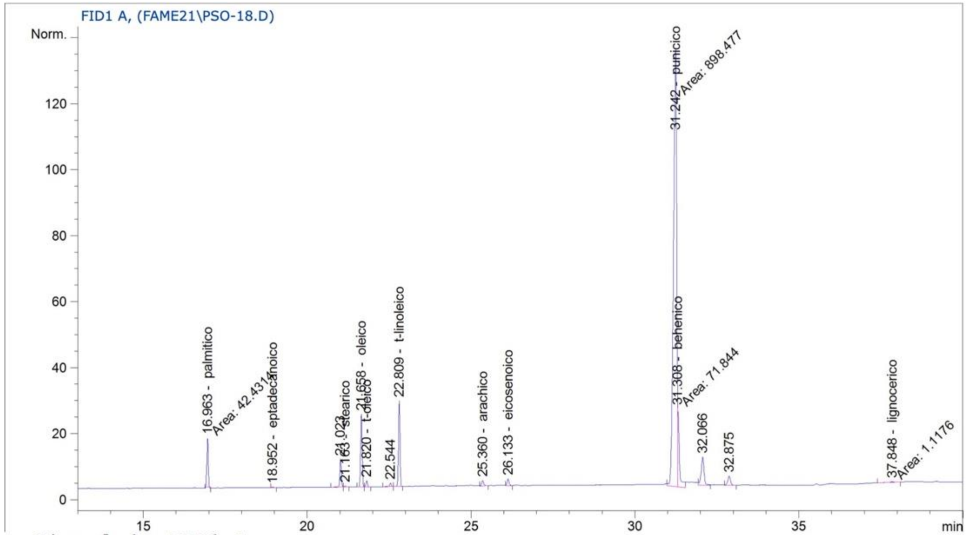

Signal 1: FID1 A,

Peak #	RetTime [min]	Type	Width [min]	Area [pA*s]	Area %	Name
1	13.454		0.0000	0.00000	0.00000	miristico
2	16.962	MM	0.0506	52.75804	3.39672	palmitico
3	17.520		0.0000	0.00000	0.00000	t-palmitoleico
4	17.931		0.0000	0.00000	0.00000	palmitoleico
5	18.948	MM	0.0496	8.27988e-1	0.05331	eptadecanoico
6	19.781		0.0000	0.00000	0.00000	eptadecenoico
7	21.019	VV	0.0501	31.52416	2.02962	?
8	21.158	VP	0.0546	1.13248	0.07291	stearico
9	21.655	VV	0.0504	82.28481	5.29775	oleico
10	21.817	VB	0.0527	6.97757	0.44924	t-oleico
11	22.183		0.0000	0.00000	0.00000	t-oleico
12	22.540	VP	0.0517	3.99488	0.25720	?
13	22.807	BB	0.0542	97.47330	6.27563	t-linoleico
14	23.059		0.0000	0.00000	0.00000	linoleico
15	24.060		0.0000	0.00000	0.00000	t-linolenico
16	24.443		0.0000	0.00000	0.00000	linolenico
17	25.357	VB	0.0619	7.97196	0.51326	arachico
18	26.128	VB	0.0656	10.24386	0.65953	eicosenoico
19	30.970	VV	0.1149	5.75914	0.37079	behenico
20	31.251	VV	0.1097	1163.92432	74.93698	punicico
21	32.060	VV	0.0911	61.09984	3.93379	?
22	32.870	BP	0.0855	18.71712	1.20506	?
23	38.127	VV	0.1737	8.51467	0.54820	lignocerico

Figure S31. GC-FID chromatogram of FAMEs in PSO-18 sample.

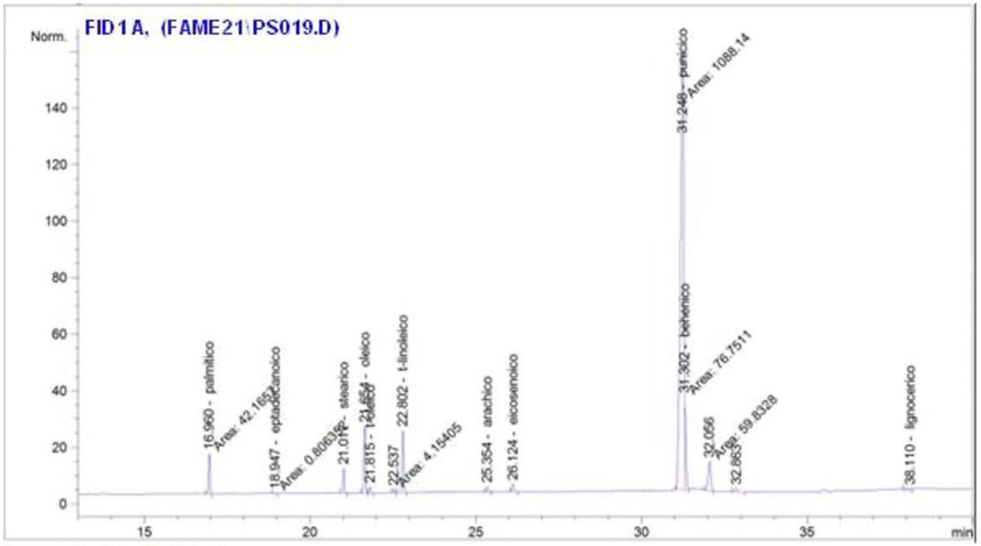

Signal 1: FID1 A,

Peak #	RetTime [min]	Type	Width [min]	Area [pA*s]	Area %	Name
1	13.454		0.0000	0.00000	0.00000	miristico
2	16.960	MM	0.0487	42.16571	2.82820	palmitico
3	17.520		0.0000	0.00000	0.00000	t-palmitoleico
4	17.931		0.0000	0.00000	0.00000	palmitoleico
5	18.947	MM	0.0514	8.06352e-1	0.05408	eptadecanoico
6	19.781		0.0000	0.00000	0.00000	eptadecenoico
7	21.017	VV	0.0502	28.65899	1.92226	stearico
8	21.654	VV	0.0508	77.37626	5.18989	oleico
9	21.815	VP	0.0506	6.99174	0.46896	t-oleico
10	22.183		0.0000	0.00000	0.00000	t-oleico
11	22.537	MM	0.0579	4.15405	0.27863	?
12	22.802	VB	0.0507	72.06013	4.83332	t-linoleico
13	23.059		0.0000	0.00000	0.00000	linoleico
14	24.060		0.0000	0.00000	0.00000	t-linolenico
15	24.443		0.0000	0.00000	0.00000	linolenico
16	25.354	VV	0.0627	7.83386	0.52544	arachico
17	26.124	VB	0.0662	11.82820	0.79336	eicosenoico
18	31.248	MF	0.1163	1088.14099	72.98530	punicico
19	31.302	FM	0.0388	76.75108	5.14795	behenico
20	32.056	MM	0.0942	59.83276	4.01319	?
21	32.863	VB	0.0956	7.64760	0.51295	?
22	38.110	VV	0.1792	6.65658	0.44648	lignocerico

Figure S32. GC-FID chromatogram of FAMEs in PSO-19 sample.

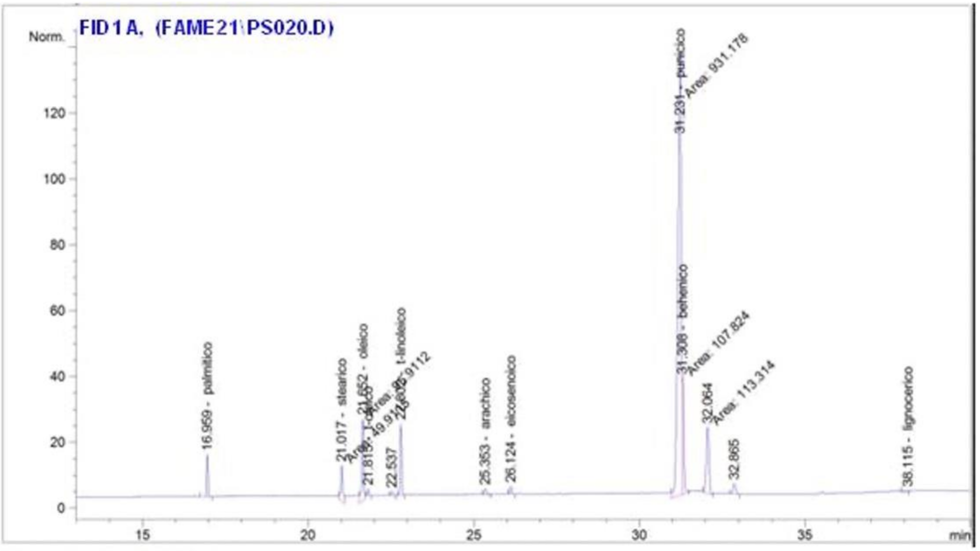

Signal 1: FID1 A,

Peak #	RetTime [min]	Type	Width [min]	Area [pA*s]	Area %	Name
1	13.454		0.0000	0.00000	0.00000	miristico
2	16.959	VP	0.0474	38.16679	2.61582	palmitico
3	17.520		0.0000	0.00000	0.00000	t-palmitoleico
4	17.931		0.0000	0.00000	0.00000	palmitoleico
5	19.107		0.0000	0.00000	0.00000	eptadecanoico
6	19.781		0.0000	0.00000	0.00000	eptadecenoico
7	21.017	MM	0.0757	49.91147	3.42076	stearico
8	21.652	MM	0.0638	95.91119	6.57342	oleico
9	21.815	VP	0.0501	6.12510	0.41979	t-oleico
10	22.183		0.0000	0.00000	0.00000	t-oleico
11	22.537	PP	0.0536	3.77839	0.25896	?
12	22.802	BB	0.0510	71.64050	4.90999	t-linoleico
13	23.059		0.0000	0.00000	0.00000	linoleico
14	24.060		0.0000	0.00000	0.00000	t-linolenico
15	24.443		0.0000	0.00000	0.00000	linolenico
16	25.353	VV	0.0649	7.45058	0.51064	arachico
17	26.124	VB	0.0657	9.74406	0.66782	eicosenoico
18	31.231	MF	0.1146	931.17841	63.81969	punicico
19	31.308	FM	0.0509	107.82378	7.38986	behenico
20	32.064	MM	0.0942	113.31428	7.76616	?
21	32.865	BB	0.0883	17.87079	1.22480	?
22	38.115	VV	0.1599	6.16163	0.42230	lignocerico

Figure S33. GC-FID chromatogram of FAMEs in PSO-20 sample.

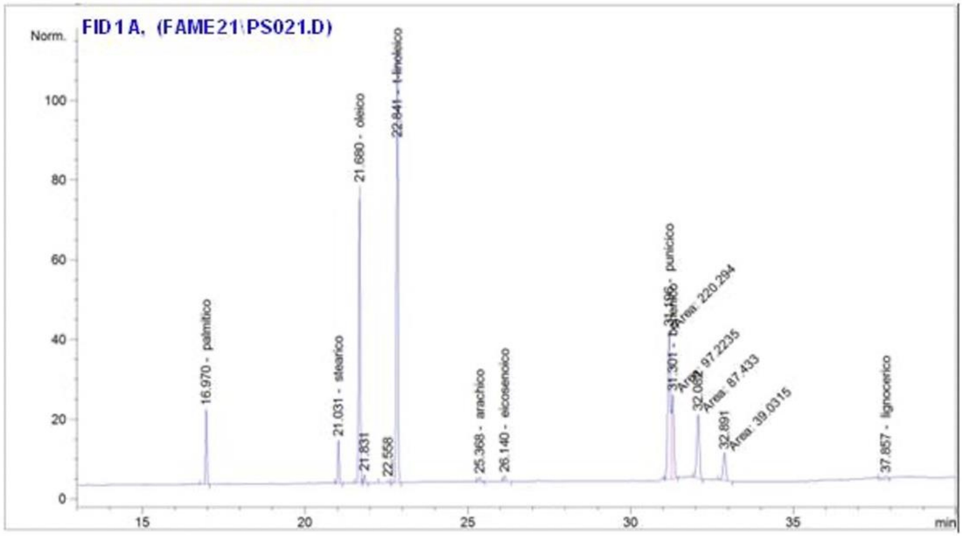

Signal 1: FID1 A,

Peak #	RetTime [min]	Type	Width [min]	Area [pA*s]	Area %	Name
1	13.454		0.0000	0.00000	0.00000	miristico
2	16.970	PB	0.0469	55.86669	4.65884	palmitico
3	17.520		0.0000	0.00000	0.00000	t-palmitoleico
4	17.931		0.0000	0.00000	0.00000	palmitoleico
5	19.107		0.0000	0.00000	0.00000	eptadecanoico
6	19.781		0.0000	0.00000	0.00000	eptadecenoico
7	21.031	VB	0.0501	34.94046	2.91376	stearico
8	21.500		0.0000	0.00000	0.00000	t-oleico
9	21.680	VV	0.0513	248.26915	20.70366	oleico
10	21.831	VV	0.0499	6.65815	0.55524	?
11	22.183		0.0000	0.00000	0.00000	t-oleico
12	22.558	VP	0.0568	2.00021	0.16680	?
13	22.841	VB	0.0559	384.40778	32.05654	t-linoleico
14	23.059		0.0000	0.00000	0.00000	linoleico
15	24.060		0.0000	0.00000	0.00000	t-linolenico
16	24.443		0.0000	0.00000	0.00000	linolenico
17	25.368	VB	0.0694	5.32512	0.44407	arachico
18	26.140	VB	0.0635	6.09487	0.50826	eicosenoico
19	31.196	MF	0.0978	220.29388	18.37075	punicico
20	31.301	FM	0.0765	97.22355	8.10767	behenico
21	32.082	MM	0.0909	87.43296	7.29121	?
22	32.891	MM	0.0935	39.03148	3.25491	?
23	37.857	VV	0.1809	11.61140	0.96830	lignocerico

Figure S34. GC-FID chromatogram of FAMEs in PSO-21 sample.

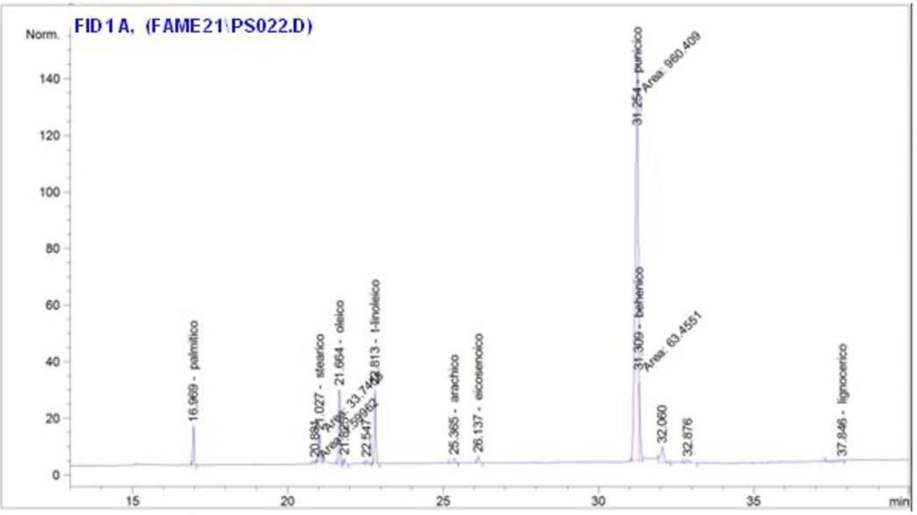

Signal 1: FID1 A,

Peak #	RetTime [min]	Type	Width [min]	Area [pA*s]	Area %	Name
1	13.454		0.0000	0.00000	0.00000	miristico
2	16.969	BB	0.0473	41.36796	3.02591	palmitico
3	17.520		0.0000	0.00000	0.00000	t-palmitoleico
4	17.931		0.0000	0.00000	0.00000	palmitoleico
5	19.107		0.0000	0.00000	0.00000	eptadecanoico
6	19.781		0.0000	0.00000	0.00000	eptadecenoico
7	20.881	MM	0.0673	1.59962	0.11701	?
8	21.027	MM	0.0580	33.74583	2.46838	stearico
9	21.500		0.0000	0.00000	0.00000	t-oleico
10	21.664	VV	0.0473	84.88789	6.20924	oleico
11	21.825	VB	0.0503	5.99419	0.43845	?
12	22.183		0.0000	0.00000	0.00000	t-oleico
13	22.547	VB	0.0578	4.09894	0.29982	?
14	22.813	BB	0.0536	88.20469	6.45185	t-linoleico
15	23.059		0.0000	0.00000	0.00000	linoleico
16	24.060		0.0000	0.00000	0.00000	t-linolenico
17	24.443		0.0000	0.00000	0.00000	linolenico
18	25.365	BV	0.0612	6.61809	0.48409	arachico
19	26.137	VP	0.0614	9.83613	0.71948	eicosenoico
20	31.254	MF	0.1092	960.40857	70.25037	punicico
21	31.309	FM	0.0344	63.45509	4.64151	behenico
22	32.060	VB	0.1098	41.75057	3.05390	?
23	32.876	VB	0.1063	8.04074	0.58815	?
24	37.846	VV	0.3208	17.11417	1.25184	lignocerico

Figure S35. GC-FID chromatogram of FAMEs in PSO-22 sample.

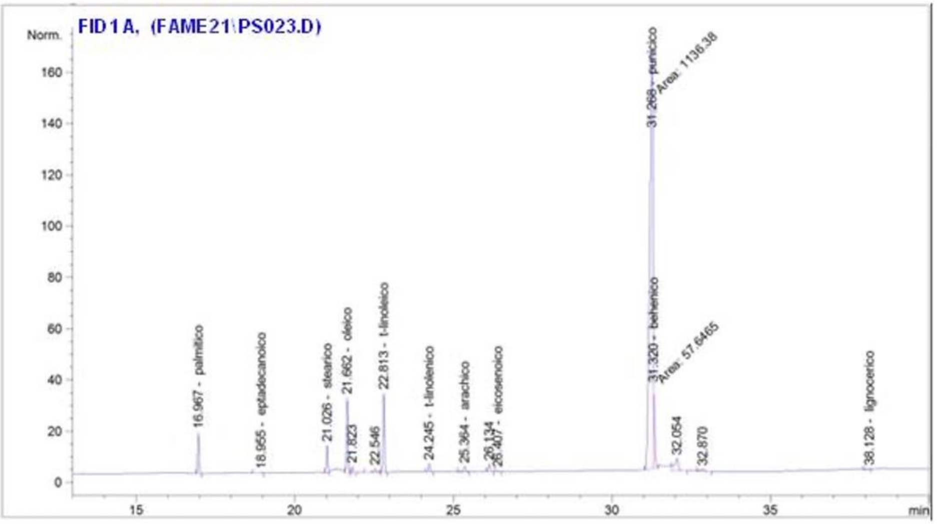

Signal 1: FID1 A,

Peak #	RetTime [min]	Type	Width [min]	Area [pA*s]	Area %	Name
1	13.454		0.0000	0.00000	0.00000	miristico
2	16.967	PB	0.0442	47.03426	2.97916	palmitico
3	17.520		0.0000	0.00000	0.00000	t-palmitoleico
4	17.931		0.0000	0.00000	0.00000	palmitoleico
5	18.955	BP	0.0511	9.35450e-1	0.05925	eptadecanoico
6	19.781		0.0000	0.00000	0.00000	eptadecenoico
7	21.026	VV	0.0532	34.81901	2.20544	stearico
8	21.500		0.0000	0.00000	0.00000	t-oleico
9	21.662	VV	0.0506	95.10838	6.02418	oleico
10	21.823	VB	0.0515	7.15448	0.45317	?
11	22.183		0.0000	0.00000	0.00000	t-oleico
12	22.546	VV	0.0725	7.12044	0.45101	?
13	22.813	VB	0.0511	101.91060	6.45504	t-linoleico
14	23.059		0.0000	0.00000	0.00000	linoleico
15	24.245	VB	0.0590	12.34761	0.78210	t-linolenico
16	24.443		0.0000	0.00000	0.00000	linolenico
17	25.364	BB	0.0699	9.25165	0.58600	arachico
18	26.134	VB	0.0641	12.18571	0.77185	?
19	26.407	BB	0.0849	1.77102	0.11218	eicosenoico
20	31.268	MF	0.1147	1136.37573	71.97827	punicico
21	31.320	FM	0.0300	57.64653	3.65134	behenico
22	32.054	VB	0.1280	43.02905	2.72547	?
23	32.870	VP	0.1099	6.11007	0.38701	?
24	38.128	VV	0.1694	5.97614	0.37853	lignocerico

Figure S36. GC-FID chromatogram of FAMEs in PSO-23 sample.

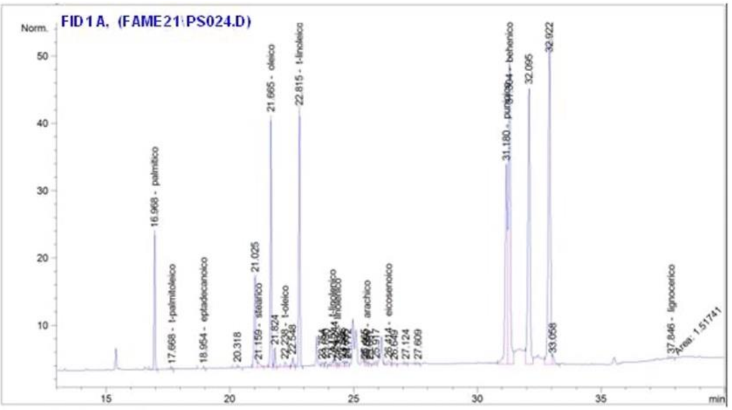

Signal 1: FID1 A,

Peak #	RetTime [min]	Type	Width [min]	Area [pA*s]	Area %	Name
1	13.454		0.0000	0.00000	0.00000	miristico
2	16.968	VB	0.0467	60.52050	4.22344	palmitico
3	17.668	VV	0.0488	1.03228	0.07204	t-palmitoleico
4	17.931		0.0000	0.00000	0.00000	palmitoleico
5	18.954	VB	0.0559	1.41762	0.09893	eptadecanoico
6	19.781		0.0000	0.00000	0.00000	eptadecenoico
7	20.318	PB	0.0641	1.78545	0.12460	?
8	21.025	VV	0.0669	64.00162	4.46637	?
9	21.159	VV	0.0971	4.98575	0.34793	stearico
10	21.500		0.0000	0.00000	0.00000	t-oleico
11	21.665	VV	0.0508	122.81997	8.57102	oleico
12	21.824	VV	0.0503	9.30140	0.64910	?
13	22.238	VV	0.0795	4.14875	0.28952	t-oleico
14	22.548	VV	0.0562	4.81941	0.33632	?
15	22.815	VB	0.0547	132.21677	9.22677	t-linoleico
16	23.059		0.0000	0.00000	0.00000	linoleico
17	23.754	VV	0.0722	2.71645	0.18957	?
18	23.890	VV	0.0671	3.19621	0.22305	?
19	24.151	VV	0.0682	3.24716	0.22660	t-linolenico
20	24.244	VV	0.0604	6.65349	0.46431	?
21	24.362	VV	0.0681	1.36567	0.09530	linolenico
22	24.566	VV	0.0934	5.04689	0.35220	?
23	24.685	VV	0.0663	2.45617	0.17140	?
24	24.772	VV	0.0684	2.77493	0.19365	?
25	25.465	VV	0.0608	2.25478	0.15735	?
26	25.550	VV	0.0634	1.84285	0.12860	arachico
27	25.661	VV	0.0784	1.93072	0.13474	?
28	25.917	VV	0.0753	2.83927	0.19814	?
29	26.414	VV	0.1193	5.04097	0.35179	eicosenoico
30	26.649	VV	0.1245	2.43745	0.17010	?
31	27.124	VV	0.0839	1.74071	0.12148	?
32	27.609	VV	0.0918	1.67455	0.11686	?
33	31.180	VV	0.0915	182.48399	12.73468	punicico
34	31.304	VV	0.0818	252.89340	17.64822	behenico
35	32.095	VV	0.0893	244.51610	17.06360	?
36	32.922	VV	0.0923	290.46475	20.27014	?
37	33.058	VB	0.0786	6.82520	0.47630	?
38	37.846	MM	0.0915	1.51741	0.10589	lignocerico

Figure S37. GC-FID chromatogram of FAMEs in PSO-24 sample.

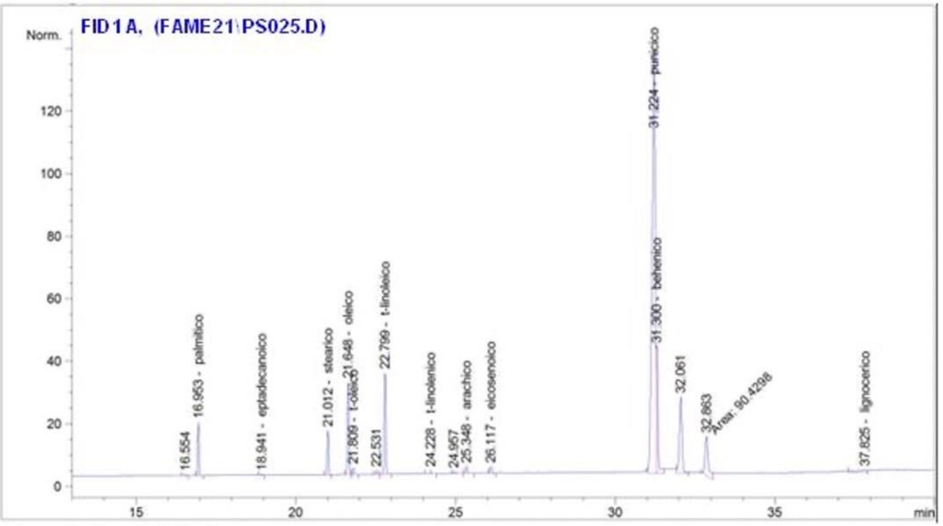

Signal 1: FID1 A,

Peak #	RetTime [min]	Type	Width [min]	Area [pA*s]	Area %	Name
1	13.454		0.0000	0.00000	0.00000	miristico
2	16.554	PV	0.0512	1.12139	0.06752	?
3	16.953	PB	0.0467	50.02279	3.01202	palmitico
4	17.520		0.0000	0.00000	0.00000	t-palmitoleico
5	17.931		0.0000	0.00000	0.00000	palmitoleico
6	18.941	BV	0.0501	1.06079	0.06387	eptadecanoico
7	19.781		0.0000	0.00000	0.00000	eptadecenoico
8	21.012	VV	0.0494	44.54191	2.68200	stearico
9	21.648	VV	0.0528	96.19995	5.79249	oleico
10	21.809	VP	0.0504	6.89609	0.41523	t-oleico
11	22.183		0.0000	0.00000	0.00000	t-oleico
12	22.531	VP	0.0539	3.99358	0.24047	?
13	22.799	VB	0.0537	107.81712	6.49199	t-linoleico
14	23.059		0.0000	0.00000	0.00000	linoleico
15	24.228	VP	0.0592	3.85055	0.23185	t-linolenico
16	24.443		0.0000	0.00000	0.00000	linolenico
17	24.957	VV	0.0871	3.63110	0.21864	?
18	25.348	VB	0.0593	8.16912	0.49189	arachico
19	26.117	VV	0.0653	8.70250	0.52400	eicosenoico
20	31.224	VV	0.1005	938.58673	56.51514	punicico
21	31.300	VV	0.0558	135.19650	8.14059	behenico
22	32.061	VB	0.0910	143.00349	8.61067	?
23	32.863	MM	0.1207	90.42981	5.44505	?
24	37.825	VV	0.2970	17.54705	1.05656	lignocerico

Figure S38. GC-FID chromatogram of FAMEs in PSO-25 sample.

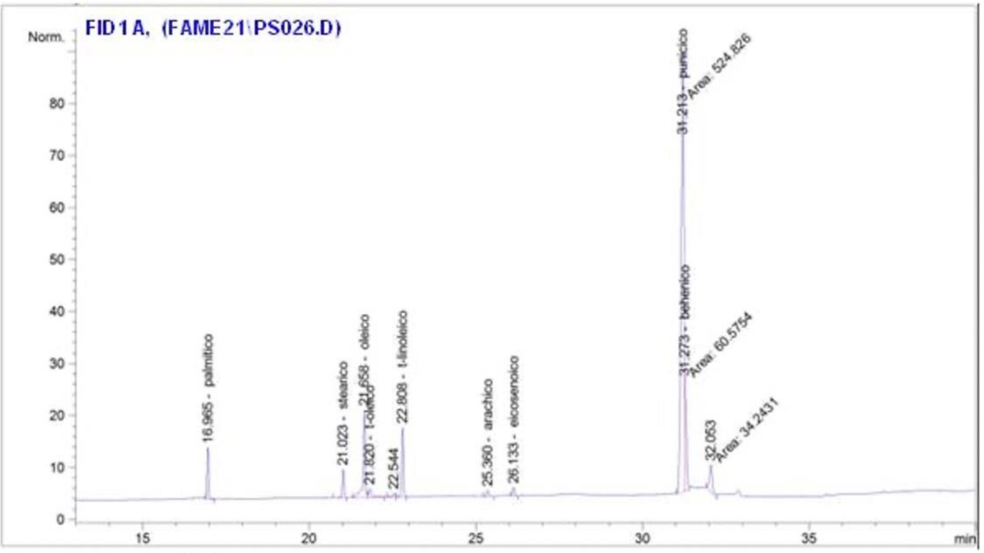

Signal 1: FID1 A,

Peak #	RetTime [min]	Type	Width [min]	Area [pA*s]	Area %	Name
1	13.454		0.0000	0.00000	0.00000	miristico
2	16.965	VV	0.0490	31.46835	3.86447	palmitico
3	17.520		0.0000	0.00000	0.00000	t-palmitoleico
4	17.931		0.0000	0.00000	0.00000	palmitoleico
5	19.107		0.0000	0.00000	0.00000	eptadecanoico
6	19.781		0.0000	0.00000	0.00000	eptadecenoico
7	21.023	PV	0.0520	18.57936	2.28164	stearico
8	21.658	VV	0.0646	71.69086	8.80400	oleico
9	21.820	VV	0.0850	10.54302	1.29474	t-oleico
10	22.183		0.0000	0.00000	0.00000	t-oleico
11	22.544	VV	0.0658	3.53716	0.43438	?
12	22.808	VV	0.0559	46.99254	5.77092	t-linoleico
13	23.059		0.0000	0.00000	0.00000	linoleico
14	24.060		0.0000	0.00000	0.00000	t-linolenico
15	24.443		0.0000	0.00000	0.00000	linolenico
16	25.360	VB	0.0664	5.19890	0.63845	arachico
17	26.133	VB	0.0621	6.64393	0.81591	eicosenoico
18	31.213	MF	0.1031	524.82635	64.45131	punicico
19	31.273	FM	0.0471	60.57536	7.43896	behenico
20	32.053	MM	0.1093	34.24314	4.20523	?
21	38.096		0.0000	0.00000	0.00000	lignocerico

Figure S39. GC-FID chromatogram of FAMEs in PSO-26 sample.

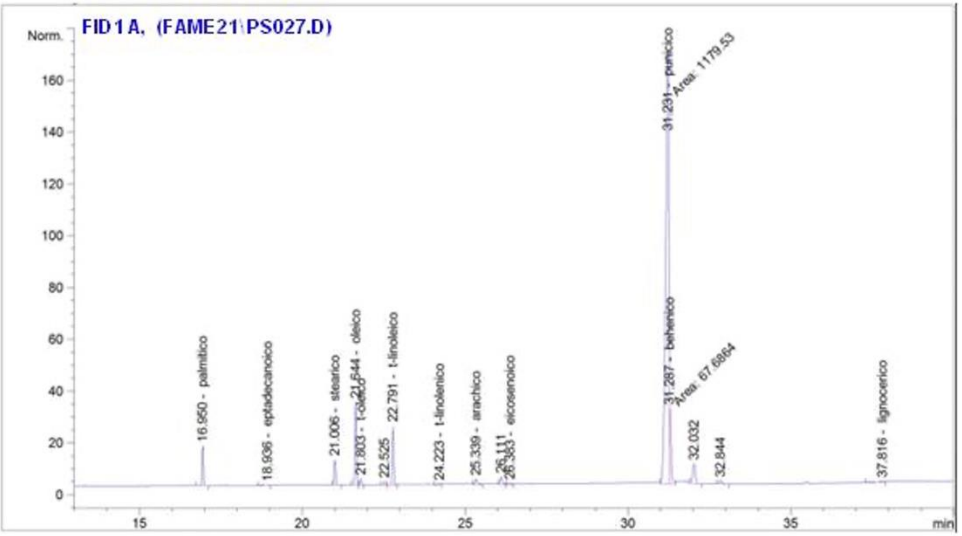

Signal 1: FID1 A,

Peak #	RetTime [min]	Type	Width [min]	Area [pA*s]	Area %	Name
1	13.454		0.0000	0.00000	0.00000	miristico
2	16.950	BB	0.0471	45.97673	2.87116	palmitico
3	17.520		0.0000	0.00000	0.00000	t-palmitoleico
4	17.931		0.0000	0.00000	0.00000	palmitoleico
5	18.936	VB	0.0512	9.17360e-1	0.05729	eptadecanoico
6	19.781		0.0000	0.00000	0.00000	eptadecenoico
7	21.006	BP	0.0498	31.47252	1.96540	stearico
8	21.644	VV	0.0499	102.51798	6.40206	oleico
9	21.803	VV	0.0506	7.67787	0.47947	t-oleico
10	22.183		0.0000	0.00000	0.00000	t-oleico
11	22.525	VV	0.0531	4.06711	0.25398	?
12	22.791	VV	0.0535	75.52543	4.71643	t-linoleico
13	23.059		0.0000	0.00000	0.00000	linoleico
14	24.223	BV	0.1378	2.65164	0.16559	t-linolenico
15	24.443		0.0000	0.00000	0.00000	linolenico
16	25.339	VB	0.0754	10.61598	0.66295	arachico
17	26.111	VB	0.0685	13.56508	0.84711	?
18	26.383	BV	0.1130	3.33466	0.20824	eicosenoico
19	31.231	MF	0.1170	1179.53467	73.65981	punicico
20	31.287	FM	0.0388	67.68643	4.22690	behenico
21	32.032	VV	0.0908	45.69516	2.85358	?
22	32.844	BP	0.0893	6.71837	0.41955	?
23	37.816	VV	0.1708	3.37042	0.21048	lignocerico

Figure S40. GC-FID chromatogram of FAMEs in PSO-27 sample.

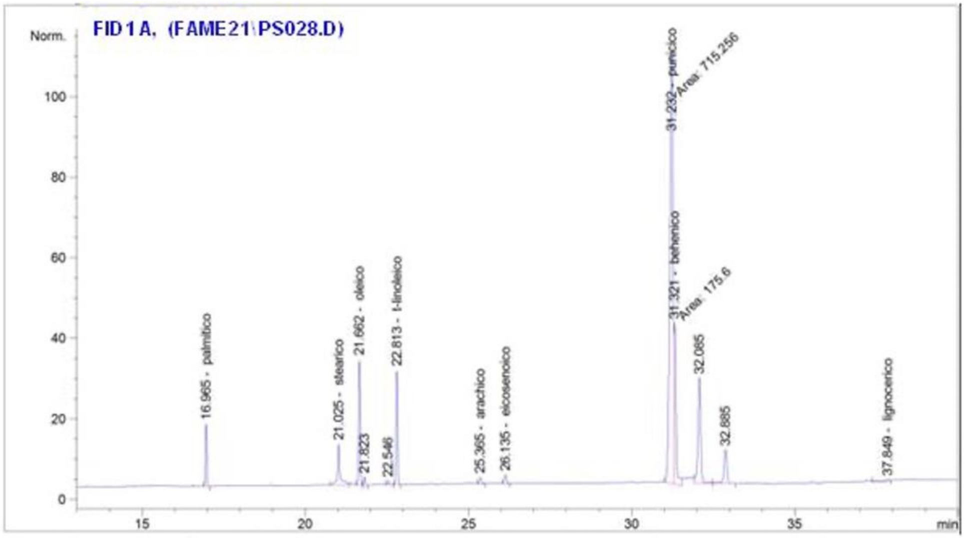

Signal 1: FID1 A,

Peak #	RetTime [min]	Type	Width [min]	Area [pA*s]	Area %	Name
1	13.454		0.0000	0.00000	0.00000	miristico
2	16.965	VB	0.0465	45.23878	3.14103	palmitico
3	17.520		0.0000	0.00000	0.00000	t-palmitoleico
4	17.931		0.0000	0.00000	0.00000	palmitoleico
5	19.107		0.0000	0.00000	0.00000	eptadecanoico
6	19.781		0.0000	0.00000	0.00000	eptadecenoico
7	21.025	VV	0.0772	56.69807	3.93667	stearico
8	21.500		0.0000	0.00000	0.00000	t-oleico
9	21.662	VV	0.0507	102.40443	7.11016	oleico
10	21.823	VB	0.0504	6.32662	0.43927	?
11	22.183		0.0000	0.00000	0.00000	t-oleico
12	22.546	VB	0.0579	3.76109	0.26114	?
13	22.813	BB	0.0507	93.17314	6.46921	t-linoleico
14	23.059		0.0000	0.00000	0.00000	linoleico
15	24.060		0.0000	0.00000	0.00000	t-linolenico
16	24.443		0.0000	0.00000	0.00000	linolenico
17	25.365	VV	0.0655	7.22461	0.50162	arachico
18	26.135	VB	0.0643	9.67528	0.67178	eicosenoico
19	31.232	MF	0.1105	715.25647	49.66179	punicico
20	31.321	FM	0.0730	175.60004	12.19229	behenico
21	32.085	VV	0.0900	157.89554	10.96302	?
22	32.885	VB	0.0987	55.17049	3.83061	?
23	37.849	VV	0.2720	11.83065	0.82143	lignocerico

Figure S41. GC-FID chromatogram of FAMEs in PSO-28 sample.

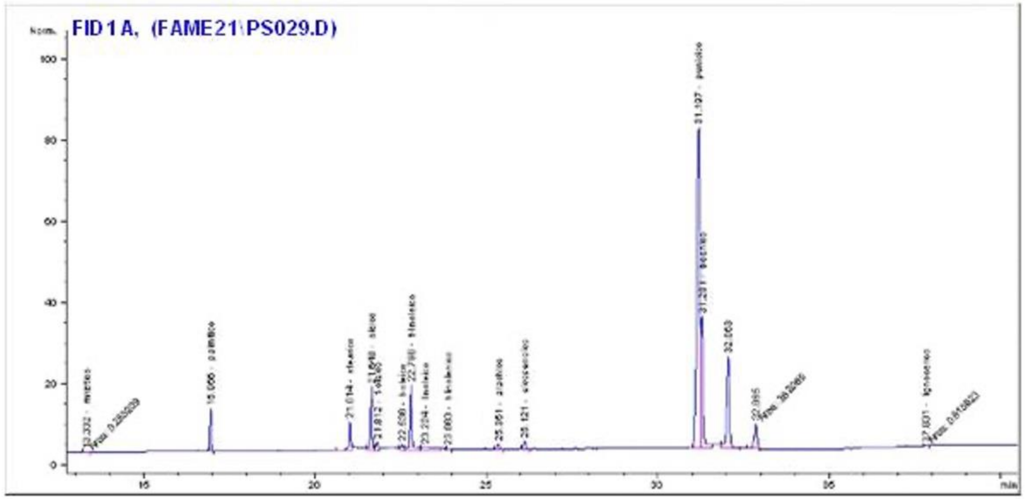

Peak #	RetTime [min]	Type	Width [min]	Area [pA*s]	Area %	Name
1	13.332	MM	0.0640	2.85209e-1	0.02702	miristico
2	16.956	VV	0.0470	31.39370	2.97465	palmitico
3	17.656		0.0000	0.00000	0.00000	t-palmitoleico
4	17.931		0.0000	0.00000	0.00000	palmitoleico
5	19.107		0.0000	0.00000	0.00000	eptadecanoico
6	19.781		0.0000	0.00000	0.00000	eptadecenoico
7	21.014	VV	0.0638	31.39359	2.97464	stearico
8	21.648	VV	0.0541	56.01372	5.30747	oleico
9	21.812	VV	0.0681	8.91848	0.84505	t-oleico
10	22.536	VV	0.0774	7.16220	0.67864	t-oleico
11	22.798	VV	0.0616	67.49591	6.39544	t-linoleico
12	23.224	VV	0.3340	16.89696	1.60104	linoleico
13	23.863	VV	0.1002	2.47198	0.23423	t-linolenico
14	24.443		0.0000	0.00000	0.00000	linolenico
15	25.351	VV	0.0709	5.73683	0.54358	arachico
16	26.121	VV	0.0638	7.89040	0.74764	eicosenoico
17	31.197	VV	0.0997	505.64423	47.91131	punicico
18	31.291	VV	0.0644	143.92046	13.63689	beenico
19	32.063	VB	0.0916	133.32950	12.63337	?
20	32.865	MF	0.0995	36.20649	3.43067	?
21	37.831	MM	0.1012	6.15923e-1	0.05836	lignocerico

Figure S42. GC-FID chromatogram of FAMEs in PSO-29 sample.

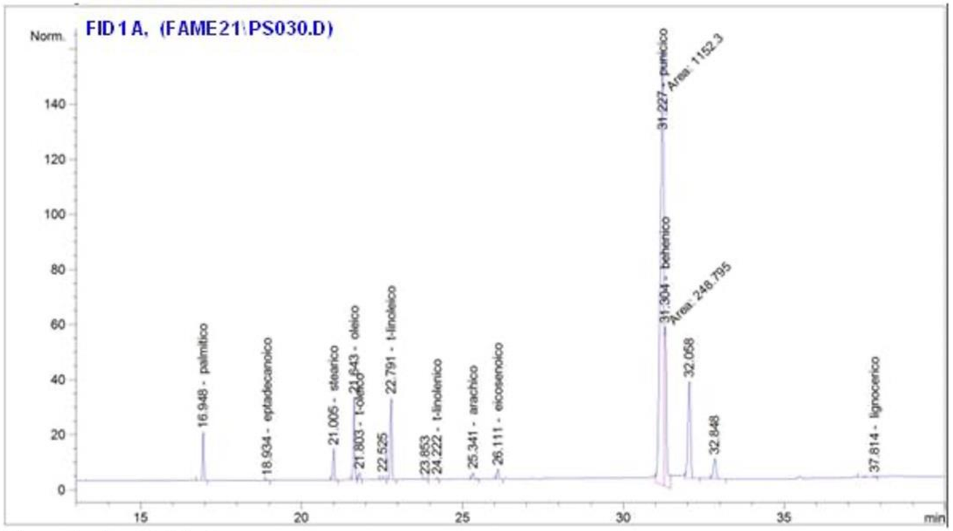

Signal 1: FID1 A,

Peak #	RetTime [min]	Type	Width [min]	Area [pA*s]	Area %	Name
1	13.454		0.0000	0.00000	0.00000	miristico
2	16.948	VB	0.0471	52.56183	2.66154	palmitico
3	17.520		0.0000	0.00000	0.00000	t-palmitoleico
4	17.931		0.0000	0.00000	0.00000	palmitoleico
5	18.934	PB	0.0452	9.68505e-1	0.04904	eptadecanoico
6	19.781		0.0000	0.00000	0.00000	eptadecenoico
7	21.005	VB	0.0498	36.11513	1.82874	stearico
8	21.643	VV	0.0536	99.10519	5.01833	oleico
9	21.803	VV	0.0505	8.59099	0.43502	t-oleico
10	22.183		0.0000	0.00000	0.00000	t-oleico
11	22.525	VV	0.0545	4.59867	0.23286	?
12	22.791	VB	0.0543	100.18019	5.07277	t-linoleico
13	23.059		0.0000	0.00000	0.00000	linoleico
14	23.853	VV	0.0693	1.27988	0.06481	?
15	24.222	VV	0.0781	2.86439	0.14504	t-linolenico
16	24.443		0.0000	0.00000	0.00000	linolenico
17	25.341	VV	0.0642	9.84403	0.49847	arachico
18	26.111	VB	0.0626	14.77086	0.74794	eicosenoico
19	31.227	MF	0.1211	1152.30090	58.34839	punicico
20	31.304	FM	0.0716	248.79466	12.59807	behenico
21	32.058	VB	0.0853	197.55849	10.00365	?
22	32.848	BP	0.0879	41.27054	2.08979	?
23	37.814	VV	0.1692	4.05913	0.20554	lignocerico

Figure S43. GC-FID chromatogram of FAMEs in PSO-30 sample.

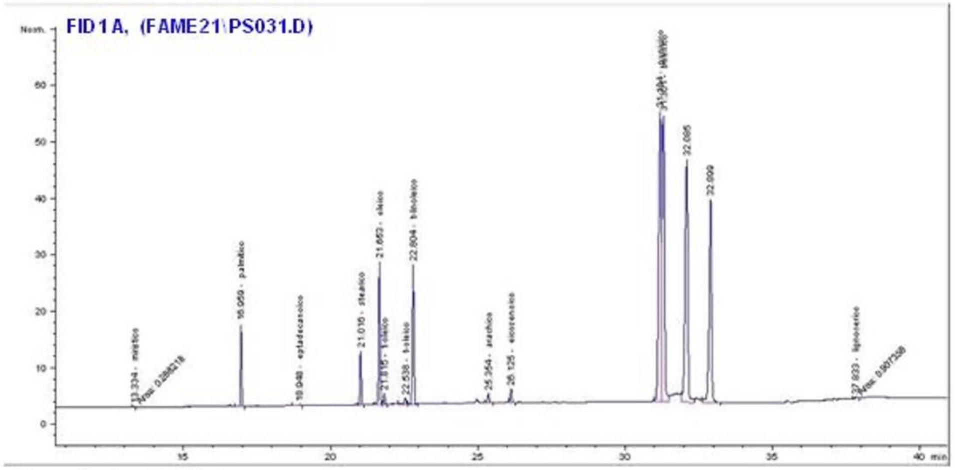

Signal 1: FID1 A,

Peak #	RetTime [min]	Type	Width [min]	Area [pA*s]	Area %	Name
1	13.334	MM	0.0484	2.68218e-1	0.02014	miristico
2	16.959	VB	0.0441	42.71738	3.20825	palmitico
3	17.656		0.0000	0.00000	0.00000	t-palmitoleico
4	17.931		0.0000	0.00000	0.00000	palmitoleico
5	18.948	BP	0.0507	8.41245e-1	0.06318	eptadecanoico
6	19.781		0.0000	0.00000	0.00000	eptadecenoico
7	21.016	VV	0.0524	30.91331	2.32172	stearico
8	21.653	VV	0.0509	83.81708	6.29501	oleico
9	21.815	VB	0.0529	6.89921	0.51816	t-oleico
10	22.538	VV	0.0607	4.72357	0.35476	t-oleico
11	22.804	VB	0.0517	83.13610	6.24387	t-linoleico
12	23.059		0.0000	0.00000	0.00000	linoleico
13	24.060		0.0000	0.00000	0.00000	t-linolenico
14	24.443		0.0000	0.00000	0.00000	linolenico
15	25.354	VV	0.0635	6.71012	0.50396	arachico
16	26.125	VP	0.0620	9.86635	0.74100	eicosenoico
17	31.184	VV	0.0979	322.10785	24.19164	punicico
18	31.301	VV	0.0776	262.61166	19.72322	beenico
19	32.085	VV	0.0901	259.24307	19.47023	?
20	32.899	VP	0.0928	216.72188	16.27671	?
21	37.833	MM	0.0907	9.07356e-1	0.06815	lignocerico

Figure S44. GC-FID chromatogram of FAMEs in PSO-31 sample.

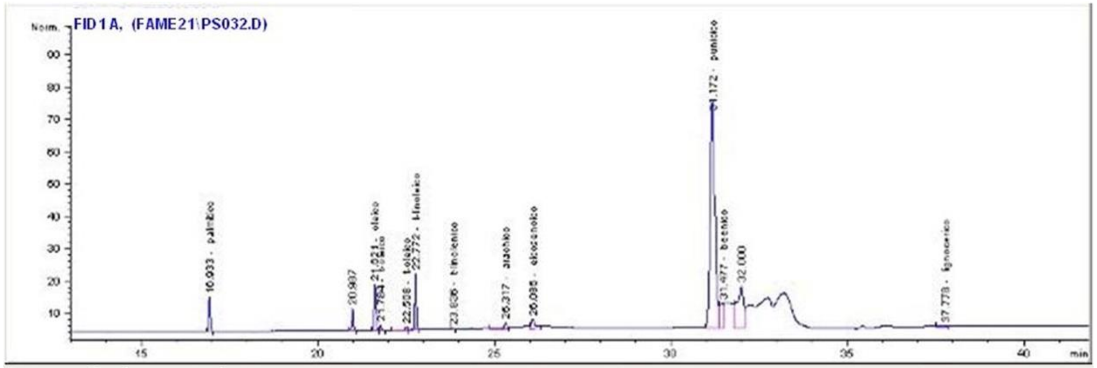

Signal 1: FID1 A,

Peak #	RetTime [min]	Type	Width [min]	Area [pA*s]	Area %	Name
1	13.454		0.0000	0.00000	0.00000	miristico
2	16.933	VB	0.0468	30.65531	2.86548	palmitico
3	17.656		0.0000	0.00000	0.00000	t-palmitoleico
4	17.931		0.0000	0.00000	0.00000	palmitoleico
5	19.107		0.0000	0.00000	0.00000	eptadecanoico
6	19.781		0.0000	0.00000	0.00000	eptadecenoico
7	20.987	VB	0.0495	21.58584	2.01772	?
8	21.318		0.0000	0.00000	0.00000	stearico
9	21.621	VV	0.0503	46.41059	4.33820	oleico
10	21.784	VP	0.0533	5.32578	0.49782	t-oleico
11	22.508	VV	0.0513	3.17204	0.29650	t-oleico
12	22.772	VV	0.0517	58.36521	5.45565	t-linoleico
13	23.059		0.0000	0.00000	0.00000	linoleico
14	23.836	VV	0.0883	1.81766	0.16990	t-linolenico
15	24.443		0.0000	0.00000	0.00000	linolenico
16	25.317	VV	0.1071	14.12401	1.32023	arachico
17	26.086	VV	0.1082	24.07653	2.25054	eicosenoico
18	31.172	VV	0.1290	625.03131	58.42434	punicico
19	31.477	VV	0.1076	65.40681	6.11385	beenico
20	32.000	VV	0.1692	169.12489	15.80883	?
21	37.778	VV	0.1909	4.71717	0.44093	lignocerico

Figure S45. GC-FID chromatogram of FAMEs in PSO-32 sample.
